# Same, Same, but Different—Whole Blood Transcriptomes of ANCA-Associated Vasculitis and Giant Cell Arteritis

**DOI:** 10.3390/ijms27198513

**Published:** 2026-09-24

**Authors:** Jennifer Amsler, Marco Kreuzer, Liubov Petelytska, Sara Andrea Krättli, Oleg Iaremenko, Matthias Brunner, Daniel Guggisberg, Lisa Christ, Matthias Gasser, Fabio Ryser, Adela Sarbu, Luca Seitz, Pascal Seitz, Britta Maurer, Kerstin Klein

**Affiliations:** 1 Department of Rheumatology and Immunology, Inselspital, Bern University Hospital, University of Bern, 3010 Bern, Switzerland; 2 Graduate School for Health Sciences, University of Bern, 3010 Bern, Switzerland; 3 Interfaculty Bioinformatics Unit, University of Bern, 3010 Bern, Switzerland; 4 Department of Internal Medicine with a Course in Cardiology and Rheumatology, Bogomolets National Medical University, 01601 Kyiv, Ukraine; 5 Department for Biomedical Research, University of Bern, 3010 Bern, Switzerland

**Keywords:** transcriptome, giant cell arteritis, ANCA-associated vasculitis, platelets

## Abstract

ANCA-associated small vessel vasculitis (AAV) and giant cell arteritis (GCA) are two clinically distinct forms of vasculitides with a high risk of relapse. We aimed to evaluate and compare the role of immune cells in patients with both vasculitis subtypes with active disease. Whole blood was collected from 12 untreated newly diagnosed or relapsing patients with AAV, 10 untreated newly diagnosed patients with GCA, and 11 age- and sex-matched healthy controls (HC). Transcriptomes were analysed by RNA sequencing. Differentially expressed genes (DEG) were calculated across disease groups. Pathway enrichment analysis was conducted using Overrepresentation Analysis (ORA), and Gene Onthology and Gene Set Enrichment Analysis (GSEA). The platelet receptor component CD42a was assessed by flow cytometry. We identified 104 DEG in AAV compared to HC, and 504 DEG between GCA and HC in whole blood samples. Strikingly, only 19 genes were differentially expressed between AAV and GCA. ORA of DEG, distinguishing both vasculitis subtypes from HC, identified pathways exclusively involved in innate immunity. GSEA of both AAV and GCA versus HC pointed to a striking overlap in up-regulated pathways comprising seven out of ten top signalling pathways, including “IL6 JAK Stat3 signalling”, “TNF signalling” and “complement activation”. We detected increased levels of CD42a on platelets of patients with GCA, in line with an increased expression of platelet-associated genes in GCA compared to AAV. Our results suggest that the rather homogeneous transcriptome of circulating immune cells does not sufficiently explain the clinically distinct phenotypes of AAV and GCA.

## 1. Introduction

ANCA-associated small vessel vasculitis (AAV) and giant cell arteritis (GCA) are two clinically distinct forms of vasculitides with a high risk of relapse. While AAV is an antibody-driven disease targeting autoantigens on neutrophils that attack the vessel walls, GCA is related to the loss of arterial wall immune tolerance, followed by aberrant immunological responses [1,2]. Additionally, in GCA, aging processes, such as immunosenescence with a reduction in regulatory T cells, as well as age-related vascular wall remodelling, are thought to play a role [1]. Beside broad-spectrum immunosuppressive treatments that can be used in both diseases, current treatment strategies fundamentally differ according to today’s pathogenetic understanding. Whereas targeting CD20 on B cells as well as C5a receptor on neutrophils are cornerstone therapies in AAV, targeting IL6 signalling is common in GCA. However, a disturbed B cell homeostasis was reported in patients with polymyalgia rheumatica (PMR) and GCA [3], and efficacy of rituximab has been confirmed in a placebo-controlled clinical trial for the treatment of PMR [4]. This might open the treatment options also towards GCA, since GCA and PMR are thought to be part of one disease spectrum [5]. On the other hand, anti-IL-6 strategies have been successfully used for the treatment of AAV [6]. Together, these results suggest that a significant proportion of the inflammatory response in AAV and GCA are overlapping.

Studies directly comparing the disease activity of AAV and GCA on a transcriptome level are missing, thus neglecting important pathophysiological insights into the similarities and differences of these disease entities. We aimed to evaluate and compare the role of circulating immune cells in patients with active disease of both vasculitis subtypes.

## 2. Results

We compared the transcriptomes of whole blood samples from 11 healthy controls (HC), 12 patients with active AAV, including newly diagnosed and relapsing patients, and 10 patients with newly diagnosed GCA (Figure 1A). PCA of RNA-seq data sets showed a clear separation of the three groups. There were no batch effects stemming from the site of blood collection, and no clustering based on AAV subgroups, CRP-levels or disease status (relapse vs. newly diagnosed) (Supplementary Figure S1). We identified 104 differentially expressed genes (DEG) in transcriptomes of patients with AAV compared to HC, and 504 DEG between GCA and HC (Figure 1B, Supplementary Tables S2 and S3). String network analysis of DEG in AAV vs. HC identified core networks associated with “neutrophil degranulation”, “leukocyte activation”, “antiviral defense” and “immunoglobulin” in AAV (Supplementary Figure S2). Overrepresentation analysis (ORA) of the 37 DEG distinguishing both vasculitis subtypes from HC identified 14 significantly up- or down-regulated pathways exclusively involved in innate immunity (Supplementary Figure S3).

ORA of DEG in AAV compared to HC identified 97 significantly up- or down-regulated pathways, including the activation of myeloid and lymphoid leukocytes (Supplementary Table S4). Nine out of ten top pathways were involved in mucosal immune response and response to bacteria, highlighting the importance of the innate immune system in AAV. In contrast, ORA of DEG in GCA vs. HC identified 230 significantly up- or down-regulated pathways with the top four involved in blood coagulation (Supplementary Table S5). However, multiple pathways, in particular those associated with leukocyte migration and activation, were overlapping with the ORA of DEG in AAV compared to HC.

To analyse this aspect in more detail, we performed a gene set enrichment analysis (GSEA) of both AAV and GCA transcriptomes versus those of HC. This pointed to a striking overlap in up-regulated pathways in AAV and GCA, comprising seven out of ten top signalling pathways (Figure 1C,D; Supplementary Tables S6 and S7), including “IL6 JAK Stat3 signalling” (Supplementary Figure S4), “TNF signalling” and “complement activation”. Interferon alpha and gamma signalling was found to be up-regulated in both diseases compared to HC (Figure 1C), though this was driven by few patients with very high interferon signatures (Supplementary Figure S5B,C) and was more pronounced in AAV than in GCA. This is also reflected by the GSEA of transcriptomes from patients with AAV versus GCA, indicating “interferon signalling” as the top differentiating pathway (Supplementary Figure S5A and Supplementary Table S8).

Strikingly, only 19 genes were differentially expressed in patients with AAV compared to GCA (Supplementary Figure S6). To determine whether the limited number of DEG between AAV and GCA reflected generally small transcriptional differences rather than solely limited statistical power, we examined genome-wide effect sizes and their uncertainty. Among 10,595 genes with a mean normalised count (baseMean) ≥10, the median absolute log2 fold change between AAV and GCA was 0.174. Overall, 66.0% of genes had an estimated absolute log2 fold change <0.25, 92.3% <0.5, and 99.5% <1, indicating that the estimated expression differences were predominantly small. Confidence intervals, demonstrated more limited precision for excluding moderate differences. The 95% confidence interval was entirely contained within ±0.5 log2 fold change for 9.5% of genes, whereas 90.2% of genes had confidence intervals contained within ±1. Thus, although the observed effect-size distribution provided little evidence for widespread large transcriptional differences between AAV and GCA, the available sample size did not allow moderate differences to be excluded for many individual genes. The results were consistent when alternative baseMean thresholds of ≥5 and ≥20 were applied.

Among the DEG between AAV and GCA, only one was associated with a B cell signature (CD24). Furthermore, HLA-DRB5, which has not yet been described as associated with AAV, was increased in AAV compared to GCA.

One of the DEG that increased in GCA, compared to AAV, was glycoprotein IX (GP9), encoding the platelet receptor component CD42a that facilitates the von Willebrand factor (vWF)-dependent adhesion of platelets to blood vessels. GSEA indicted that “platelet activation” was an up-regulated pathway in GCA compared to AAV (Figure 2A, Supplementary Table S5), with several platelet-associated genes being higher expressed in GCA compared to AAV (Figure 2B). Platelet numbers in GCA and AAV were similar in the cohort used for the transcriptome analysis (Supplementary Figure S7). However, we confirmed an increased expression of CD42a on platelets by flow cytometry in GCA compared to HC in a partially overlapping second cohort of patients (Figure 2C). To evaluate whether expression differences of platelet-associated genes solely reflected differences in platelet numbers, we conducted a correlation analysis of platelet counts and expression levels of the 20 platelet-associated genes (Supplementary Table S9). Ten out of 20 genes showed a significant correlation between transcript and platelet counts. We then ran DESeq2 twice with these 20 genes, once with ~group + age + sex (original analysis) and once with ~group + age + sex + platelet-count-scaled. The median absolute log2 fold change decreased from 1.16 before adjustment to 0.72 after adjustment, corresponding to a median attenuation of 24.2%. Despite a partial contribution of platelet counts to the platelet-associated genes, substantial differences in the platelet transcriptional profiles between groups remained.

## 3. Discussion

To our knowledge, this is the first explorative study directly comparing the blood transcriptomes of AAV and GCA patients and healthy individuals. In line with the prominent role of B cells and neutrophils in AAV [2], the top enriched biological processes in the ORA of transcriptomes from AAV compared to HC were “humoral immune response” and “bacterial defense response”. However, when comparing whole blood transcriptomes of untreated AAV and GCA patients, we found a strong overlap of activated signalling pathways, with limited differences in B and neutrophil signatures in the blood of newly diagnosed or relapsing AAV compared to newly diagnosed GCA patients. This is surprising, given the current distinct pathophysiological concepts for AAV and GCA, pointing to B cells and neutrophils in AAV [7], and dendritic cells, T cells and macrophages in GCA as key cell types [1]. Similarities were strongest in pathways involved in the innate immune system, which might highlight a more important role of innate immune cells, such as neutrophils in GCA, than previously expected. Since only samples from patients with GCA and no other subtypes of LVV were included, our findings cannot be generalised towards other subtypes of LVV.

A recent study has compared the blood transcriptomes of 30 AAV and 142 systemic lupus erythematosus (SLE) patients and has found a 40% overlap between the DEG of SLE and AAV [8] with a more homogenous transcriptome in AAV compared to SLE. They found an aberrant type I and II interferon (IFN) signalling in AAV. Interestingly, in our study, IFN alpha and gamma responses were increased in both vasculitis subtypes compared to HC with a slightly higher increase in AAV compared to GCA. In line with our data, a type I IFN signature has recently been described in microscopic polyangiitis (MPA)/MPO-AAV kidney biopsies [9], while IFN gamma has been described as playing a role in the recruitment of macrophages in GCA [10].

Similarly, “IL6 JAK Stat3 signalling” was increased in both diseases in our cohort. Although being thought to play a central role rather in GCA than in AAV, another study has found an activated IL-6 pathway in patients with AAV, which has even led to treatment with an anti-IL-6-receptor inhibitor with some success [6,11].

A whole blood transcriptome analysis of 17 treatment-naïve GCA patients found a gene signature of monocyte activation and B cell inactivation compared to patients with Takayasu arteritis and HC [12]. In our study, an aberrant B cell signature was found in both GCA and AAV compared to HC. Interestingly, only one DEG between AAV and GCA was associated with a B cell gene signature (CD24), which suggests a similar molecular B cell signature in both diseases.

One substantial difference of AAV and GCA patients in our analysis was an increased platelet signature in transcriptomes of patients with GCA compared to patients with AAV. An increased platelet count has long been described in GCA [13]; however, whether platelets play a role in the immune crosstalk between immune cells of the adaptive and innate immune system has not yet been elucidated. Recently, a study has shown that GCA patients have an impaired ability to regulate the clearance of extracellular mitochondria, which might contribute to excessive inflammation and platelet activation [14]. Although older recommendations argued in favour of the preventive use of aspirin in GCA, the new EULAR recommendations of 2018 do not recommend antiplatelet or anticoagulant therapy routinely based on two recent cohort studies and a meta-analysis [15]. Since we did not use platelet activation markers for the flow cytometry analysis, an independent validation of our results regarding the platelet activity in AAV and GCA is needed.

Due to the low number of patients and controls included, enabling the detection of bigger changes in transcriptome profiles of patient groups but potentially missing the detection of subtle differences, the conclusion based on this study might be limited. However, one strength of our explorative study is the inclusion of solely untreated patients. Patient samples were age- and sex-matched with healthy individuals. Due to known differences in disease onset of AAV and GCA [1,2] age-matching among these groups was not feasible. Another limiting factor might be the inclusion of two eosinophilic polyangiitis (EGPA) patients, which were ANCA-negative. Although those patients might have confounded some of our results, they clustered together with other AAV patients in the PCA of the transcriptomes. Furthermore, there was only one up-regulated gene (IL5RA) possibly related to EGPA in AAV compared to GCA. Another limitation of our study is the use of QuantSeq 3′ RNA sequencing, which captures reads mainly at the 3′ end of transcripts. While this approach allows robust gene expression quantification, it limits the ability to assess alternative splicing, transcript isoforms, and other features of full-length transcripts. Our results suggest that the rather homogeneous transcriptomes of circulating immune cells do not sufficiently explain the clinically distinct phenotypes of AAV and GCA. We identified increased platelet-associated genes as a more specific characteristic of disease activity in GCA patients, pointing to the need for future studies to clarify the platelet-immune cell interaction in this context. The absence of pronounced disease-specific transcriptional differences in whole blood should not be interpreted as evidence for an absence of GCA-specific biology but may rather reflect the compartmentalisation of disease-associated molecular signatures within the vascular wall and tissue-specific cellular interactions, consistent with findings from transcriptomic analyses of affected vascular tissues [16].

## 4. Materials and Methods

### 4.1. Patient Samples

Patients who fulfilled the ACR classification criteria of 2022 for AAV [17,18,19] or the ACR/EULAR classification criteria of 2022 for GCA [20] were recruited in the context of the Bern Vasculitis Study (BEVAS) at the Bern University Hospital or at the University Hospital Kiev between October 2023 and October 2025. The study was conducted in accordance with the Declaration of Helsinki and approved by the Ethics Committee of the Canton of Bern (2023-00208) and the Commission on Bioethical Expertise and Ethics of Scientific Research of the Bogomolets National Medical University, Kyiv, Ukraine (2020-137). All patients provided written informed consent prior the inclusion into the study. Clinical characteristics are shown in Supplementary Table S1a. Whole blood was collected from 12 untreated newly diagnosed or relapsing AAV patients, 10 untreated newly diagnosed GCA patients, and 11 age- and sex-matched HC in PAX-gene tubes (Qiagen). AAV diagnoses included 2 patients with EGPA, 5 patients with granulomatosis with polyangiitis (GPA), and 5 patients with MPA. Active disease in patients with AAV was defined as BVAS >1. Relapse was defined as an at least 1-point increase in BVAS in a patient previously in remission.

### 4.2. RNA Sequencing (RNA-Seq) of Whole Blood

Total RNA from whole blood was extracted according to the manufacturer’s protocol (Qiagen PAXgene Blood RNA Kit: Venlo, The Netherlands) and quality was evaluated by Bioanalyser measurements (Agilent Technologies, Santa Clara, CA, USA). Library preparation (Lexogen QuantSeq 3′ mRNA-Seq, Vienna, Austria) and RNA sequencing (Illumina NovaSeq 6000 (Illumina Inc., San Diego, CA, USA)) were performed at the Next Generation Sequencing Platform (NGSP), University of Bern, within one batch. Differential gene expression across disease groups was analysed using DESeq2: v1.51.6 on gene-level count data [21], with normalisation performed using DESeq2 size factors and age, sex, and disease group included in the model (~age + sex + group). Pairwise contrasts were evaluated between AAV, GCA, and HC, and genes with an absolute fold change >1.5 and a Benjamini–Hochberg adjusted *p* value < 0.05 were considered differentially expressed. Pathway enrichment was assessed using the clusterProfiler R package, v4.21.1, applying both ORA and GSEA. ClusterProfiler v4.10.0 [22] was used to identify gene ontology terms containing unusually many DEG. Gene set enrichment analysis (GSEA) [23] was run in ClusterProfiler v4.10.0 [22] using genesets from KEGG [24] and MSigDb [25]. An interactive Shiny application v1.6.0 [26] was set up to facilitate the exploration and visualisation of the RNA-seq results. All analyses were run in R version 4.3.2 (31 October 2023). To assess transcriptomic similarity independently of statistical significance thresholds, the genome-wide distribution of effect sizes and their uncertainty was assessed using genes with a DESeq2 mean normalised count (baseMean) ≥10. Robustness to this threshold was evaluated using baseMean cutoffs of ≥5 and ≥20. For each gene, the absolute log2 fold change was calculated, and the proportions of genes with absolute log2 fold changes <0.25, <0.5, and <1 were determined. Approximate 95% confidence intervals for the log2 fold-change estimates were calculated from the DESeq2 standard errors as log2 fold change ±1.96 × SE. To account for the precision of the effect-size estimates, we additionally determined the proportion of genes for which the complete 95% confidence interval was contained within prespecified bounds of ±0.25, ±0.5, and ±1 log2 fold change. These analyses were used to distinguish small estimated expression differences from sufficient statistical precision to exclude effects exceeding a given magnitude.

### 4.3. Flowcytometry Analysis

Whole blood of 20 HC, 10 patients with GCA and 9 patients with AAV without glucocorticoid treatment was collected in EDTA tubes and processed within two hours. This cohort included the 11 HC, 5 patients with AAV and 9 patients with GCA that were included in the RNAseq analysis. Clinical characteristics of patients and controls used for flow cytometry analysis are displayed in Supplementary Table S1b. After lysis of red blood cells in RBC Lysis buffer (Biolegend, Ref 420301) and blocking of cells (10% FBS in PBS), platelets were labelled with BUV 805 mouse anti-human CD42a antibodies (BD Biosciences, 748570). Cells were fixed with 1% PFA in PBS and analysed using the Cytek Aurora 5L full spectrum instrument (Cytek Biosciences, Fremont, CA, USA). Platelets were gated and mean fluorescence intensities (MFI) of CD42a-positive platelets were compared between groups. Data are shown as mean ± S.E.M. Data were analysed using the Prism GraphPad 10 software. Comparisons were made using a one-way ANOVA with Tukey‘s test for multiple comparisons. *p*-values < 0.05 were considered significant.

## Figures and Tables

**Figure 1:**
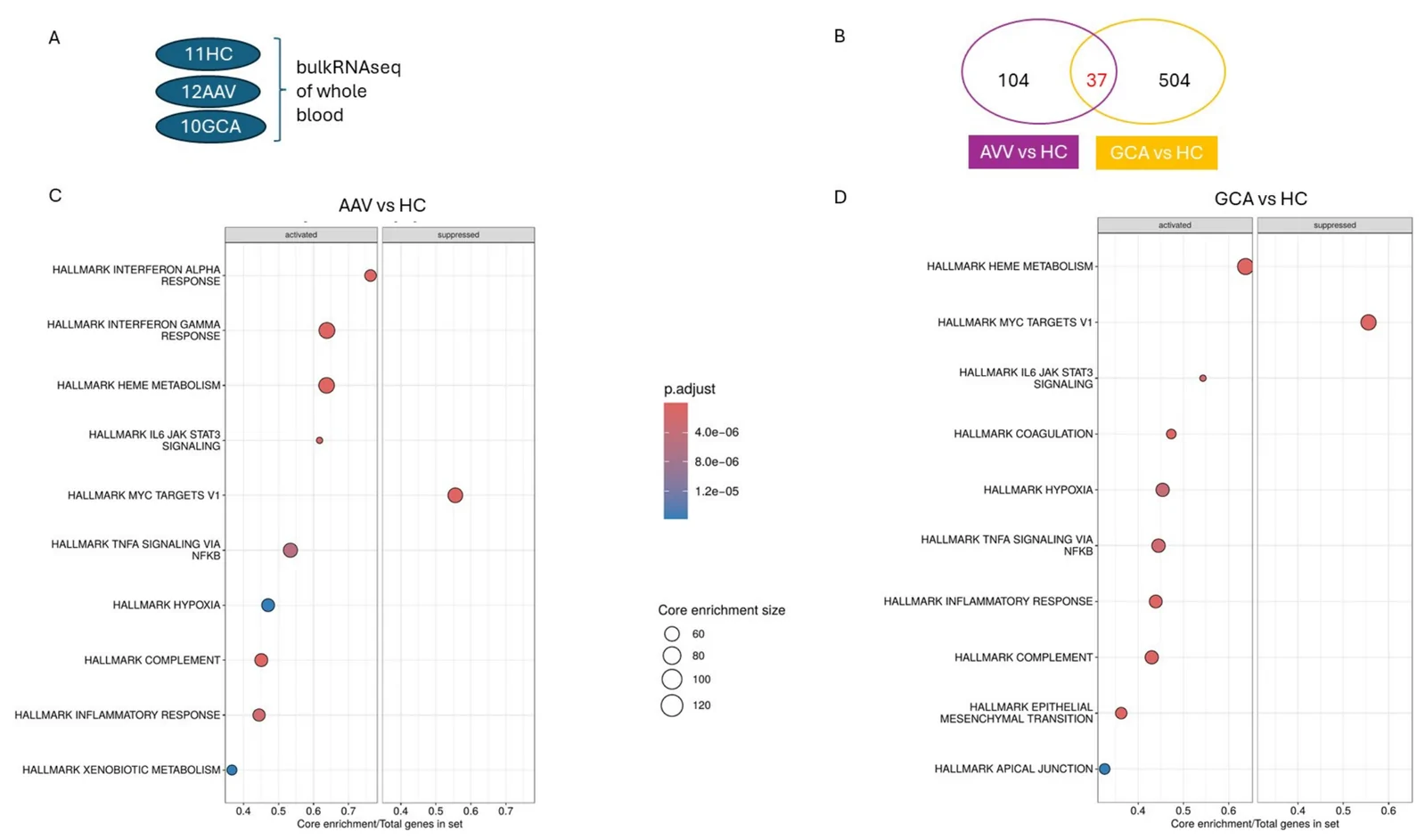
Similarities of whole blood transcriptomes of patients with AAV and GCA. (**A**) Outline of samples used in our study. (**B**) Venn diagram of DEG (log2 FC > 1.5; padj < 0.05) between AAV and HC, GCA and HC, and their overlapping DEG. Dot plots of enriched gene sets (MSigDB) in whole blood of patients with (**C**) AAV compared to HC, and (**D**) GCA compared to HC. AAV: ANCA-associated vasculitis, DEG: differentially expressed genes, FC: fold change, HC: healthy control, GCA: giant cell arteritis.

**Figure 2:**
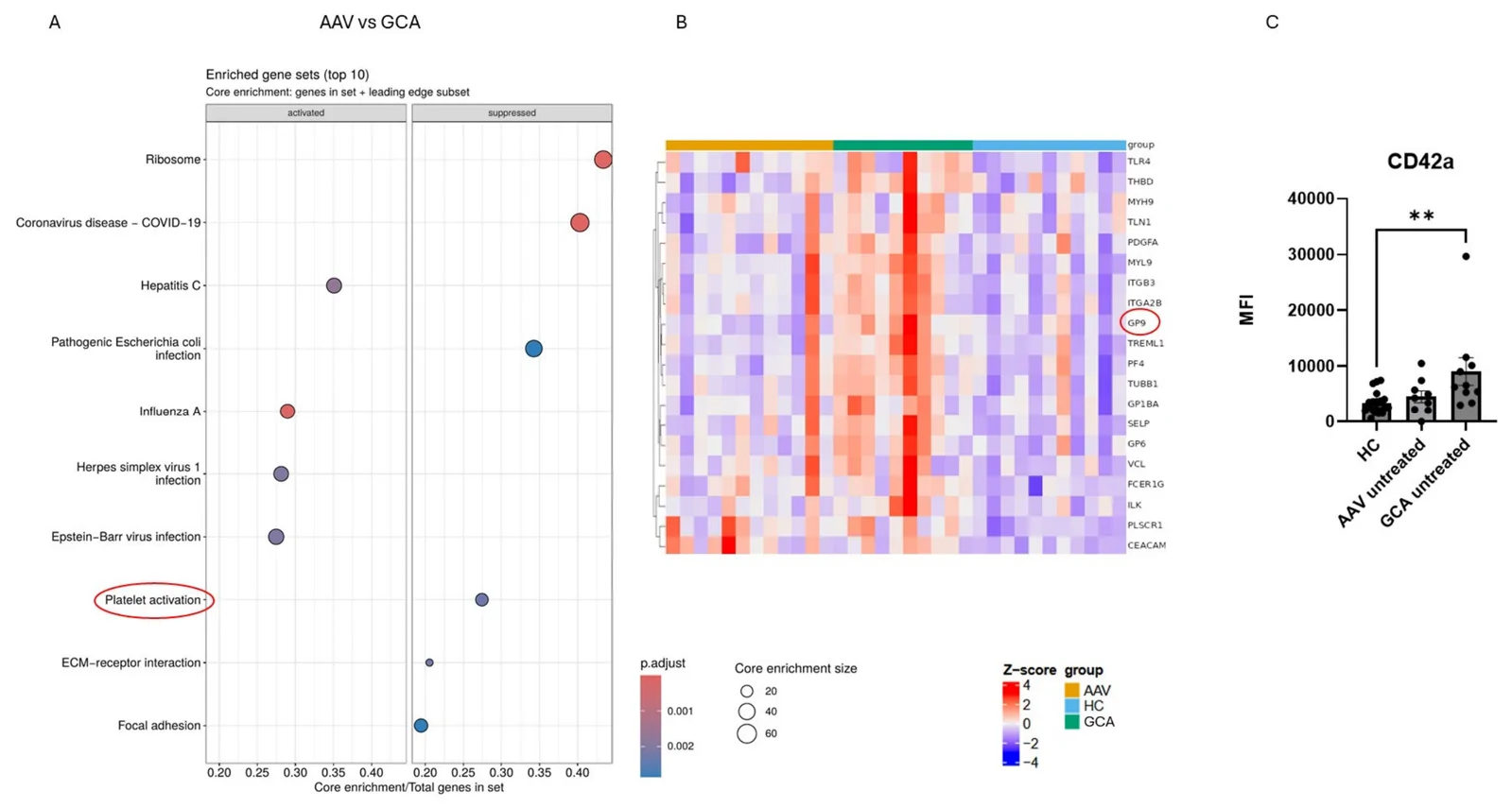
Platelet-associated genes distinguish GCA from AAV. (**A**) Dot plots of enriched gene sets (KEGG) in whole blood of patients with AAV compared to GCA. (**B**) Heatmap of DEG involved in “platelet activation” in whole blood of patients with AAV, GCA and HC. (**C**) MFI of CD42a on peripheral blood platelets of HC, glucocorticoid-naive patients with GCA and AAV. One-way ANOVA for multiple comparisons between HC, AAV and GCA (** <0.01). AAV: ANCA-associated vasculitis, DEG: differentially expressed genes, HC: healthy control, GCA: giant cell arteritis, MFI: mean fluorescence intensity.

## Data Availability

The RNA sequencing data sets generated in this study have been deposited on NCBI’s Gene Expression Omnibus [27] and are accessible through the GEO Series accession number GSE325135 upon publication (https://www.ncbi.nlm.nih.gov/geo/query/acc.cgi?acc=GSE325135, accessed on 18 August 2026).

## Supplementary Materials

The following supporting information can be downloaded at https://www.mdpi.com/article/10.3390/ijms27198513/s1.

## Abbreviations

The following abbreviations are used in this manuscript:

AAV	ANCA-associated vasculitis
DEG	Differentially expressed genes
EGPA	Eosinophilic granulomatosis with polyangiitis
GCA	Giant cell arteritis
GPA	Granulomatosis with polyangiitis
GSEA	Gene set enrichment analysis
HC	Healthy controls
IFN	Interferon
MPA	Microscopic polyangiitis
ORA	Overrepresentation analysis
PMR	Polymyalgia rheumatica

## References

[1] Pugh D., Karabayas M., Basu N., Cid M.C., Goel R., Goodyear C.S., Grayson P.C., McAdoo S.P., Mason J.C., Owen C. (2022). Large-vessel vasculitis. Nat. Rev. Dis. Primers.

[2] Kitching A.R., Anders H.J., Basu N., Brouwer E., Gordon J., Jayne D.R., Kullman J., Lyons P.A., Merkel P.A., Savage C.O. (2020). ANCA-associated vasculitis. Nat. Rev. Dis. Primers.

[3] Van Der Geest K.S., Abdulahad W.H., Chalan P., Rutgers A., Horst G., Huitema M.G., Roffel M.P., Roozendaal C., Kluin P.M., Bos N.A. (2014). Disturbed B cell homeostasis in newly diagnosed giant cell arteritis and polymyalgia rheumatica. Arthritis Rheumatol..

[4] Bolhuis T.E., Marsman D.E., Broeder A.A.D., Broeder N.D., van der Maas A. (2023). 1-year results of treatment with rituximab in polymyalgia rheumatica: An extension study of a randomised double-blind placebo-controlled trial. Lancet Rheumatol..

[5] Dejaco C., Duftner C., Buttgereit F., Matteson E.L., Dasgupta B. (2017). The spectrum of giant cell arteritis and polymyalgia rheumatica: Revisiting the concept of the disease. Rheumatology.

[6] Sakai R., Kondo T., Kurasawa T., Nishi E., Okuyama A., Chino K., Shibata A., Okada Y., Takei H., Nagasawa H. (2017). Current clinical evidence of tocilizumab for the treatment of ANCA-associated vasculitis: A prospective case series for microscopic polyangiitis in a combination with corticosteroids and literature review. Clin. Rheumatol..

[7] Amsler J., Everts-Graber J., Martin K.R., Roccabianca A., Lopes C., Tourneur L., Mocek J., Karras A., Naccache J.-M., Bonnotte B. (2024). Dysregulation of neutrophil oxidant production and interleukin-1-related cytokines in granulomatosis with polyangiitis. Rheumatology.

[8] Banos A., Thomas K., Garantziotis P., Filia A., Malissovas N., Pieta A., Nikolakis D., Panagiotopoulos A.G., Chalkia A., Petras D. (2023). The genomic landscape of ANCA-associated vasculitis: Distinct transcriptional signatures, molecular endotypes and comparison with systemic lupus erythematosus. Front. Immunol..

[9] Vieira M., Régnier P., Maciejewski-Duval A., Le Joncour A., Darasse-Jèze G., Rosenzwajg M., Klatzmann D., Cacoub P., Saadoun D. (2022). Interferon signature in giant cell arteritis aortitis. J. Autoimmun..

[10] Corbera-Bellalta M., Planas-Rigol E., Lozano E., Terrades-García N., Alba M.A., Prieto-González S., García-Martínez A., Albero R., Enjuanes A., Espígol-Frigolé G. (2016). Blocking interferon γ reduces expression of chemokines CXCL9, CXCL10 and CXCL11 and decreases macrophage infiltration in ex vivo cultured arteries from patients with giant cell arteritis. Ann. Rheum. Dis..

[11] Berti A., Cavalli G., Campochiaro C., Guglielmi B., Baldissera E., Cappio S., Sabbadini M.G., Doglioni C., Dagna L. (2015). Interleukin-6 in ANCA-associated vasculitis: Rationale for successful treatment with tocilizumab. Semin. Arthritis Rheum..

[12] Matsumoto K., Suzuki K., Yoshida H., Magi M., Matsumoto Y., Noguchi-Sasaki M., Yoshimoto K., Takeuchi T., Kaneko Y. (2023). Distinct gene signatures of monocytes and B cells in patients with giant cell arteritis: A longitudinal transcriptome analysis. Arthritis Res. Ther..

[13] Foroozan R., Danesh-Meyer H., Savino P.J., Gamble G., Mekari-Sabbagh O.N., Sergott R.C. (2002). Thrombocytosis in patients with biopsy-proven giant cell arteritis. Ophthalmology.

[14] Michailidou D., Grayson P.C., Hermanson P., Chapa J.A.G., Cuthbertson D., Khalidi N.A., Koening C.L., Langford C.A., McAlear C.A., Moreland L.W. (2023). Mitochondrial-mediated inflammation and platelet activation in giant cell arteritis. Clin. Immunol..

[15] Hellmich B., Agueda A., Monti S., Buttgereit F., de Boysson H., Brouwer E., Cassie R., Cid M.C., Dasgupta B., Dejaco C. (2020). 2018 Update of the EULAR recommendations for the management of large vessel vasculitis. Ann. Rheum. Dis..

[16] Lindquist I., Friedman M. (2026). Network analysis on transcriptome studies of large- and small-vessel vasculitis highlights SYK and CD247 as highly connected hub genes in giant cell arteritis. Ann. Rheum. Dis..

[17] Robson J.C., Grayson P.C., Ponte C., Suppiah R., Craven A., Judge A., Khalid S., Hutchings A., Watts R.A., Merkel P.A. (2022). 2022 American College of Rheumatology/European Alliance of Associations for Rheumatology classification criteria for granulomatosis with polyangiitis. Ann. Rheum. Dis..

[18] Suppiah R., Robson J.C., Grayson P.C., Ponte C., Craven A., Khalid S., Judge A., Hutchings A., Merkel P.A., ALuqmani R. (2022). 2022 American College of Rheumatology/European Alliance of Associations for Rheumatology classification criteria for microscopic polyangiitis. Ann. Rheum. Dis..

[19] Grayson P.C., Ponte C., Suppiah R., Robson J.C., Craven A., Judge A., Khalid S., Hutchings A., Luqmani R.A., Watts R.A. (2022). 2022 American College of Rheumatology/European Alliance of Associations for Rheumatology Classification Criteria for Eosinophilic Granulomatosis with Polyangiitis. Ann. Rheum. Dis..

[20] Ponte C., Grayson P.C., Robson J.C., Suppiah R., Gribbons K.B., Judge A., Craven A., Khalid S., Hutchings A., Watts R.A. (2022). 2022 American College of Rheumatology/EULAR Classification Criteria for Giant Cell Arteritis. Arthritis Rheumatol..

[21] Love M.I., Huber W., Anders S. (2014). Moderated Estimation of Fold Change and Dispersion for RNA-Seq Data with DESeq2. Genome Biol..

[22] Wu T., Hu E., Xu S., Chen M., Guo P., Dai Z., Feng T., Zhou L., Tang W., Zhan L. (2021). clusterProfiler 4.0: A universal enrichment tool for interpreting omics data. Innovation.

[23] Subramanian A., Tamayo P., Mootha V.K., Mukherjee S., Ebert B.L., Gillette M.A., Paulovich A., Pomeroy S.L., Golub T.R., Lander E.S. (2005). Gene set enrichment analysis: A knowledge-based approach for interpreting genome-wide expression profiles. Proc. Natl. Acad. Sci. USA.

[24] Kanehisa M., Furumichi M., Sato Y., Kawashima M., Ishiguro-Watanabe M. (2023). KEGG for taxonomy-based analysis of pathways and genomes. Nucleic Acids Res..

[25] Liberzon A., Birger C., Thorvaldsdóttir H., Ghandi M., Mesirov J.P., Tamayo P. (2015). The Molecular Signatures Database (MSigDB) hallmark gene set collection. Cell Syst..

[26] Chang W., Cheng J., Allaire J., Sievert C., Schloerke B., Xie Y., Allen L., McPherson J., Dipert A., Borges B. (2021). shiny: Web Application Framework for R.

[27] Edgar R., Domrachev M., Lash A.E. (2002). Gene Expression Omnibus: NCBI gene expression and hybridization array data repository. Nucleic Acids Res..

