# Supplementary material for: Same, Same, but Different—Whole Blood Transcriptomes of ANCA-Associated Vasculitis and Giant Cell Arteritis
Source: Int J Mol Sci. 2026 Sep 24;27(19):8513. doi: 10.3390/ijms27198513 (PMC13664966; doi:10.3390/ijms27198513)
Supplement: Supplementary file 1 [file ijms-27-08513-s001.zip › Supplementary Figures 15.9.26.pdf]

Supplementary figures

Same, same, but different – whole blood transcriptomes of ANCA-associated vasculitis and giant cell arteritis

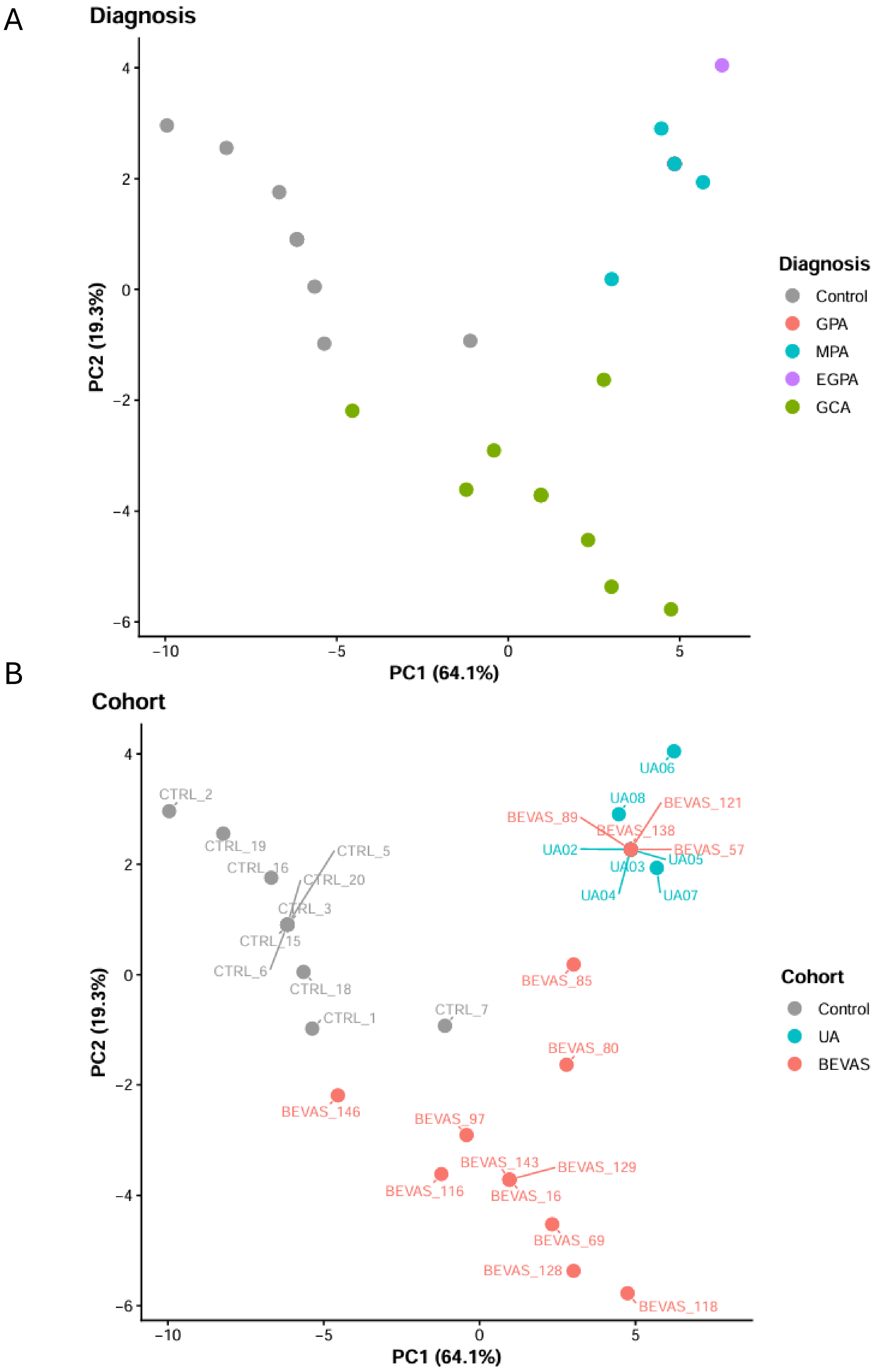

Supplementary Figure S1

C

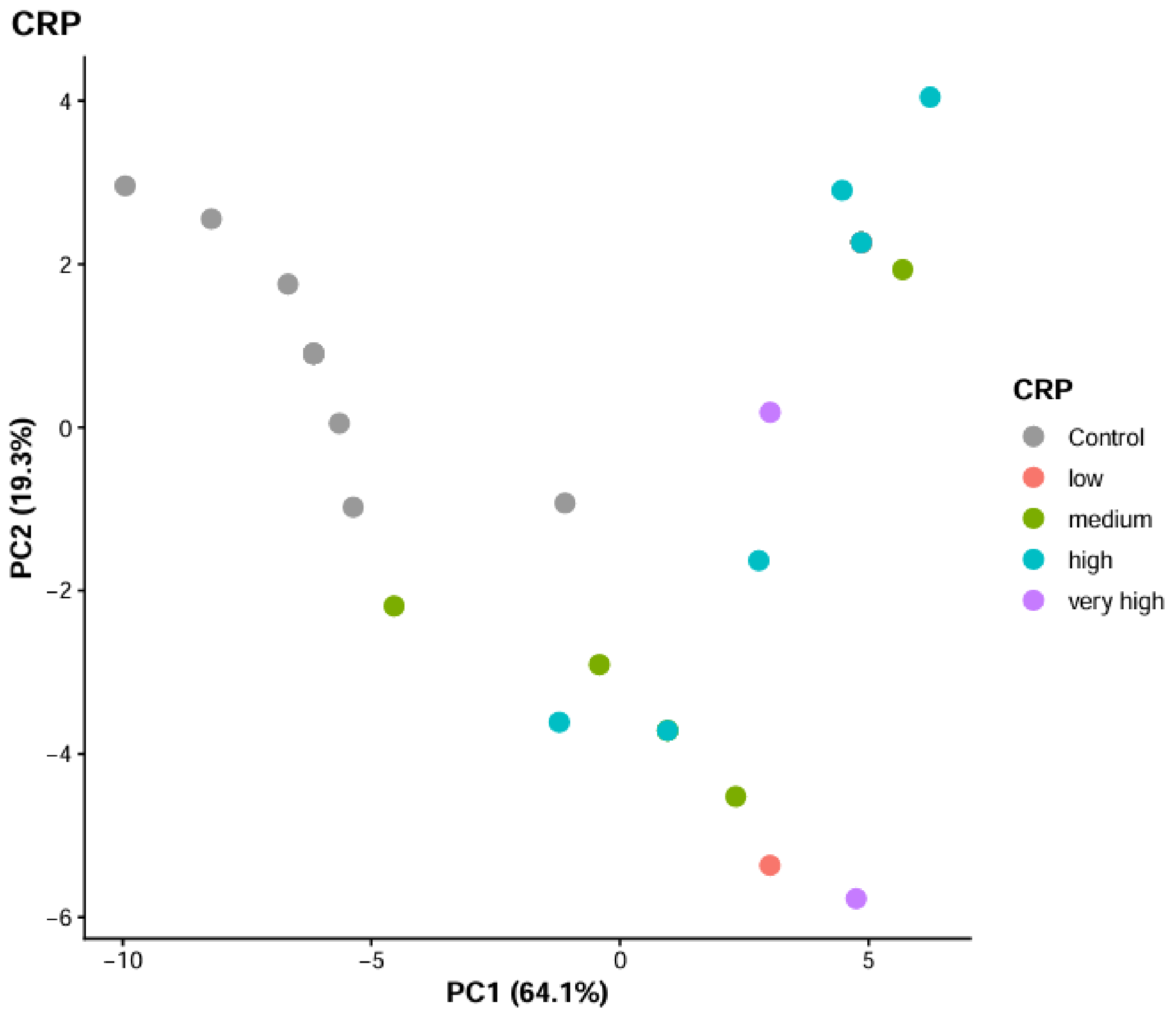

D

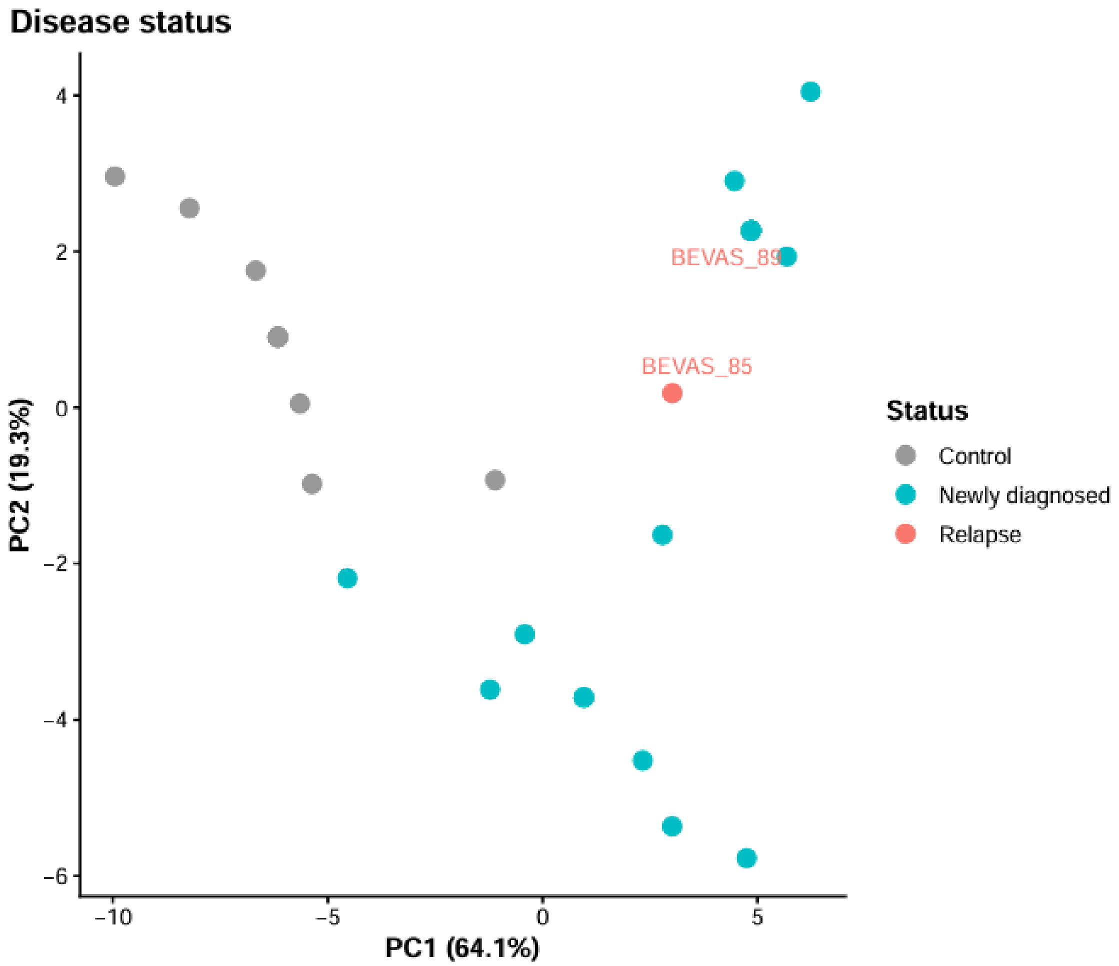

Supplementary Figure S1 following

Figure legend Supplementary Figure S1: Principal component analysis of transcriptomes in whole blood samples from 11 controls, 12 patients with ANCA-associated vasculitis and 10 patients with giant cell arteritis (GCA). Due to an overlap of several dots, more than one sample might be displayed in one dot. (A) Diagnosis: GPA: granulomatosis with polyangiitis, MPA: microscopic polyangiitis, EGPA: eosinophilic granulomatosis with polyangiitis. (B) Sample collection sites: UA: Kyiv, Ukraine; BEVAS: Bern, Switzerland. (C) CRP-levels defined as following: low <10mg/L, medium 11-49 mg/L, high 50-100mg/L, very high >100mg/L. (D) Disease status.

Supplementary Figure S1 following

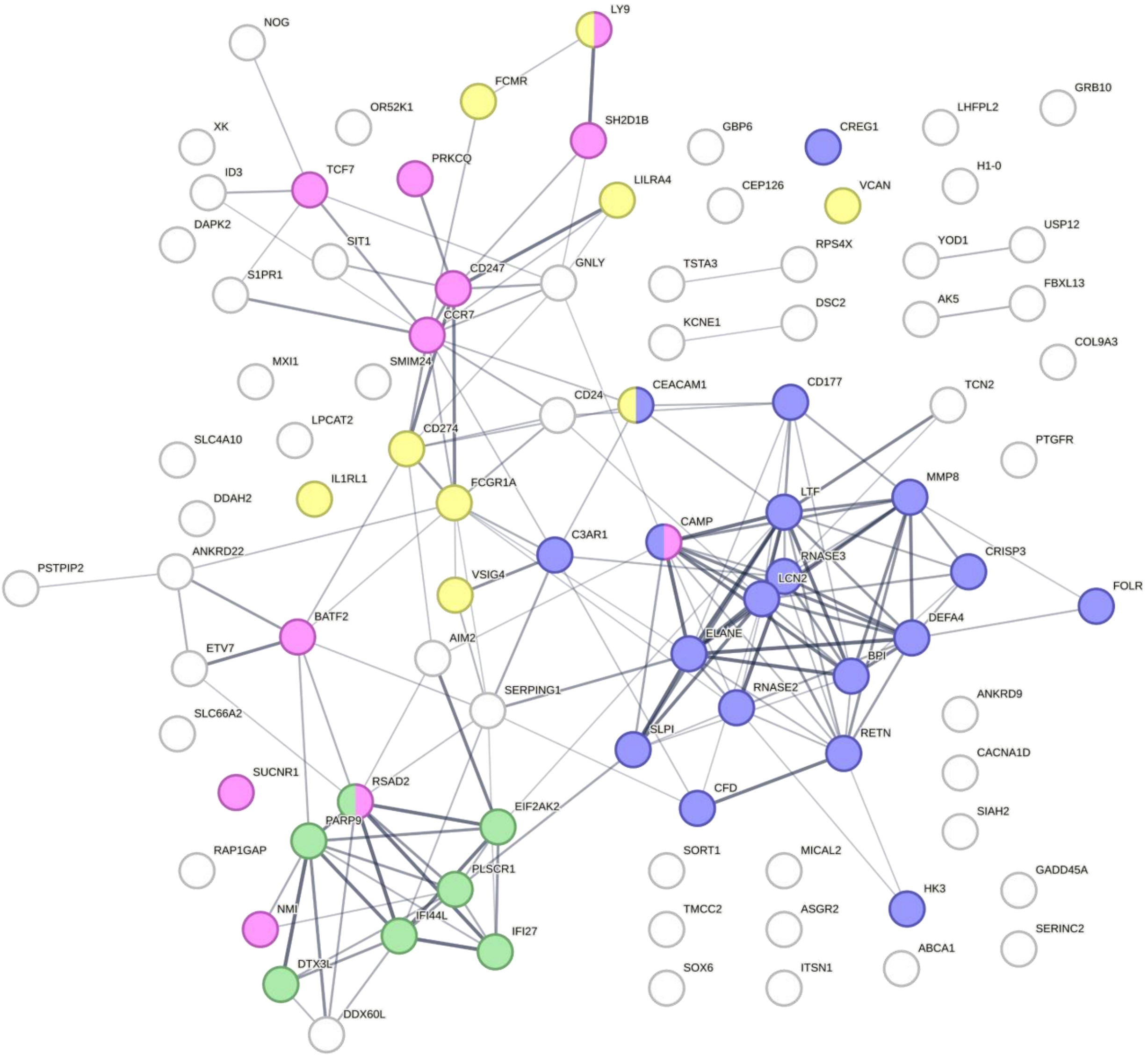

Figure legend Supplementary Figure S2: String network analysis of differentially expressed genes between AAV and HC. Colors indicating genes involved in pathways: blue: neutrophil degranulation, green: antiviral defense, yellow: immunoglobulin, pink: leukocyte activation.

Supplementary Figure S2

A

ASGR2
BPI
CAMP
CD177
CD247
CEACAM1
CFD
CREG1
CRISP3
DAPK2
DDX60L
DSC2
FBXL13
FCGR1A
FOLR3
GADD45A
GFUS
GNLY
GRB10
ID3
KCNE1
LCN2
LTF
MICAL2
MIR223HG
MMP8
PLSCR1
RETN
SH2D1B
SIAH2
SLC66A2
SMIM24
SORT1
TCN2
VCAN
VSIG4
YOD1

B

Description	p.adjust
myeloid leukocyte activation	0.00504863
defense response to bacterium	0.03719005
regulation of macrophage activation	0.04912807
humoral immune response	0.04912807
positive regulation of reactive oxygen species metabolic process	0.04912807
cell killing	0.04912807
antimicrobial humoral immune response mediated by antimicrobial peptide	0.04912807
complement activation, alternative pathway	0.04912807
regulation of response to biotic stimulus	0.04912807
negative regulation of macrophage activation	0.04912807
defense response to Gram-negative bacterium	0.04912807
negative regulation of cytokine production	0.04912807
positive regulation of myeloid leukocyte mediated immunity	0.04912807
regulation of immune effector process	0.04962165

Supplementary Figure S3

Figure legend of Supplementary Figure S3: (A) 37 differentially expressed genes (DEG) distinguishing both vasculitis subtypes from HC. (B) List of significantly dysregulated pathways resulting from pathway enrichment analysis using overrepresentation analysis (ORA) of 37 DEG.

IL6 JAK Stat3 signaling

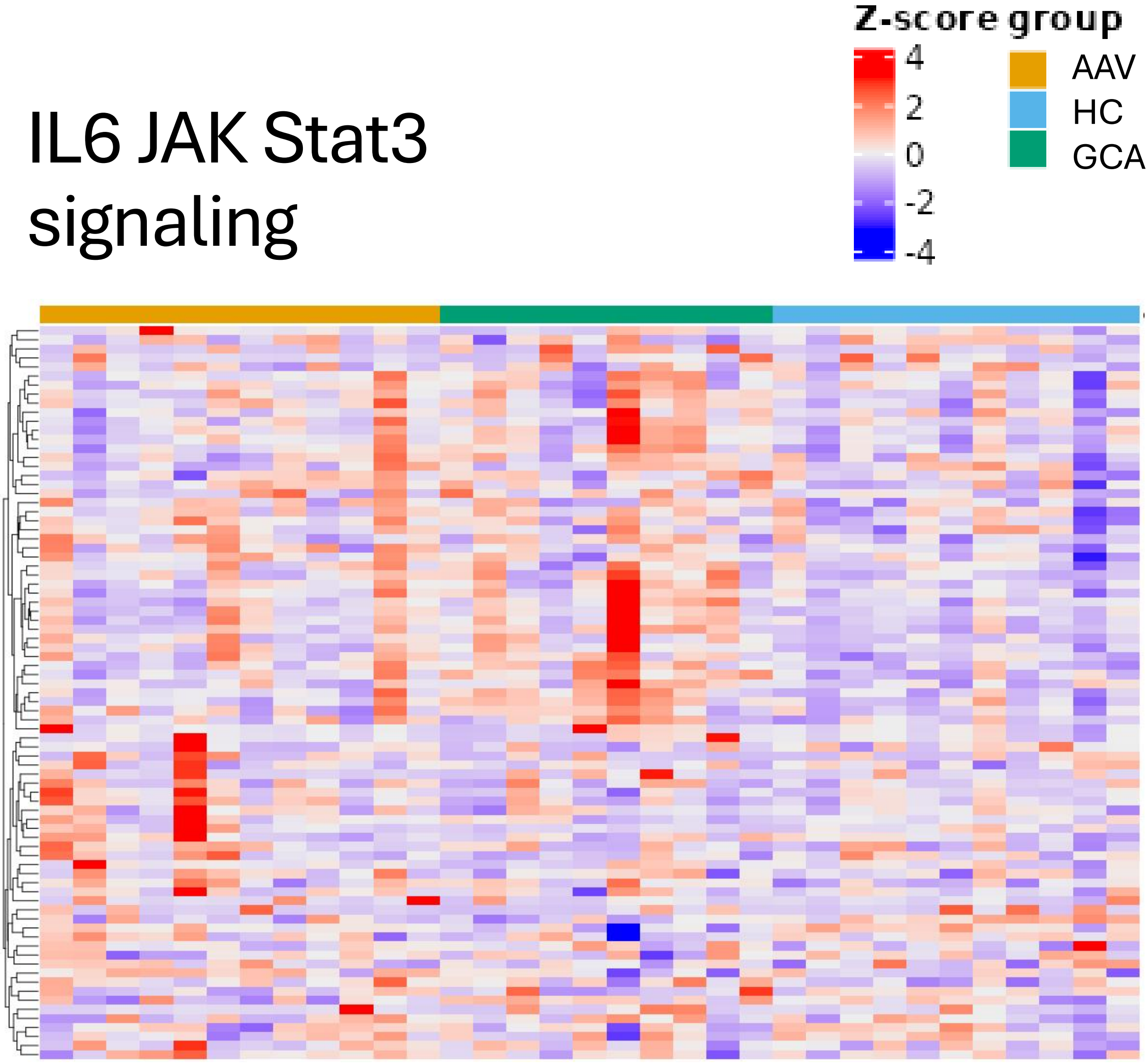

Figure legend Supplementary Figure S4: Heatmap of differentially expressed genes (DEG) involved in “IL6 JAK Stat3 signaling” in the peripheral blood of patients with ANCA-associated vasculitis (AAV), giant cell arteritis (GCA) and healthy controls (HC).

Supplementary Figure S4

A AAV vs. GCA

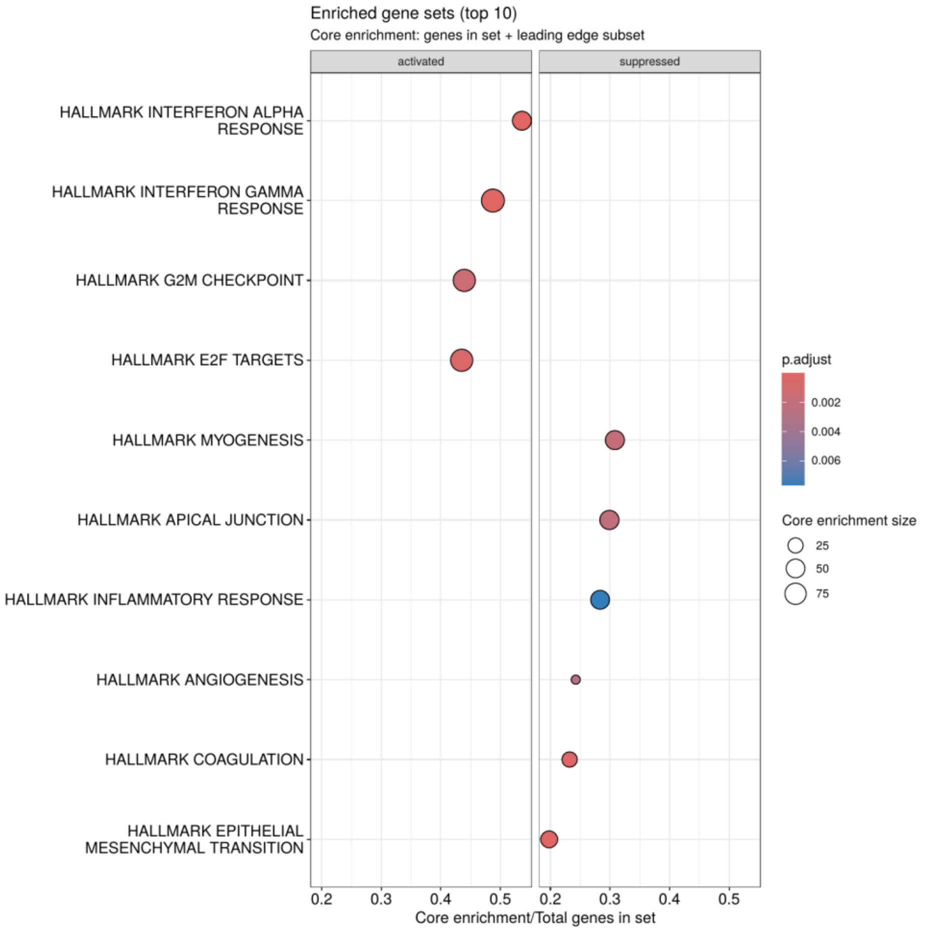

B IFNalpha response

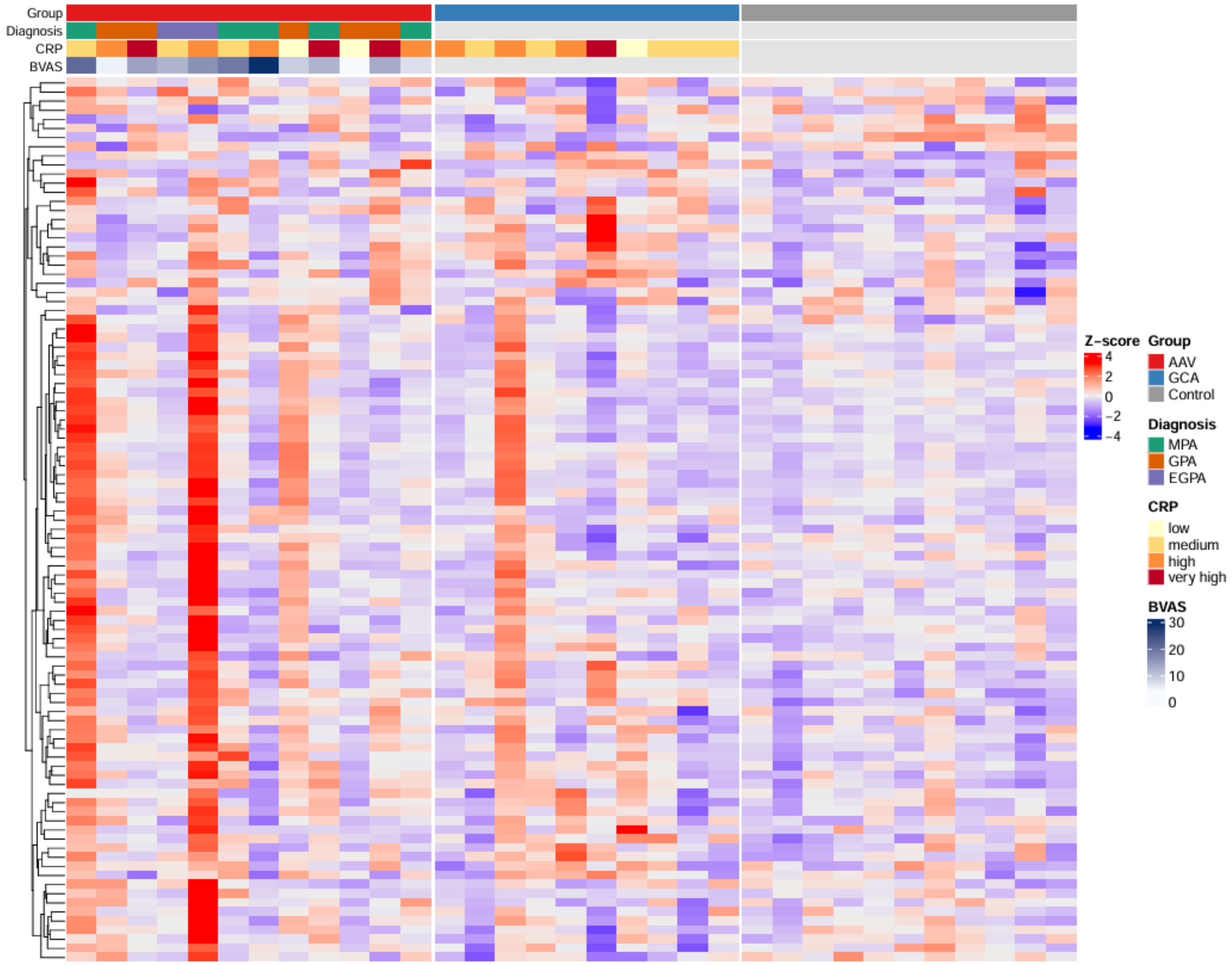

Supplementary Figure S5

C IFNgamma response

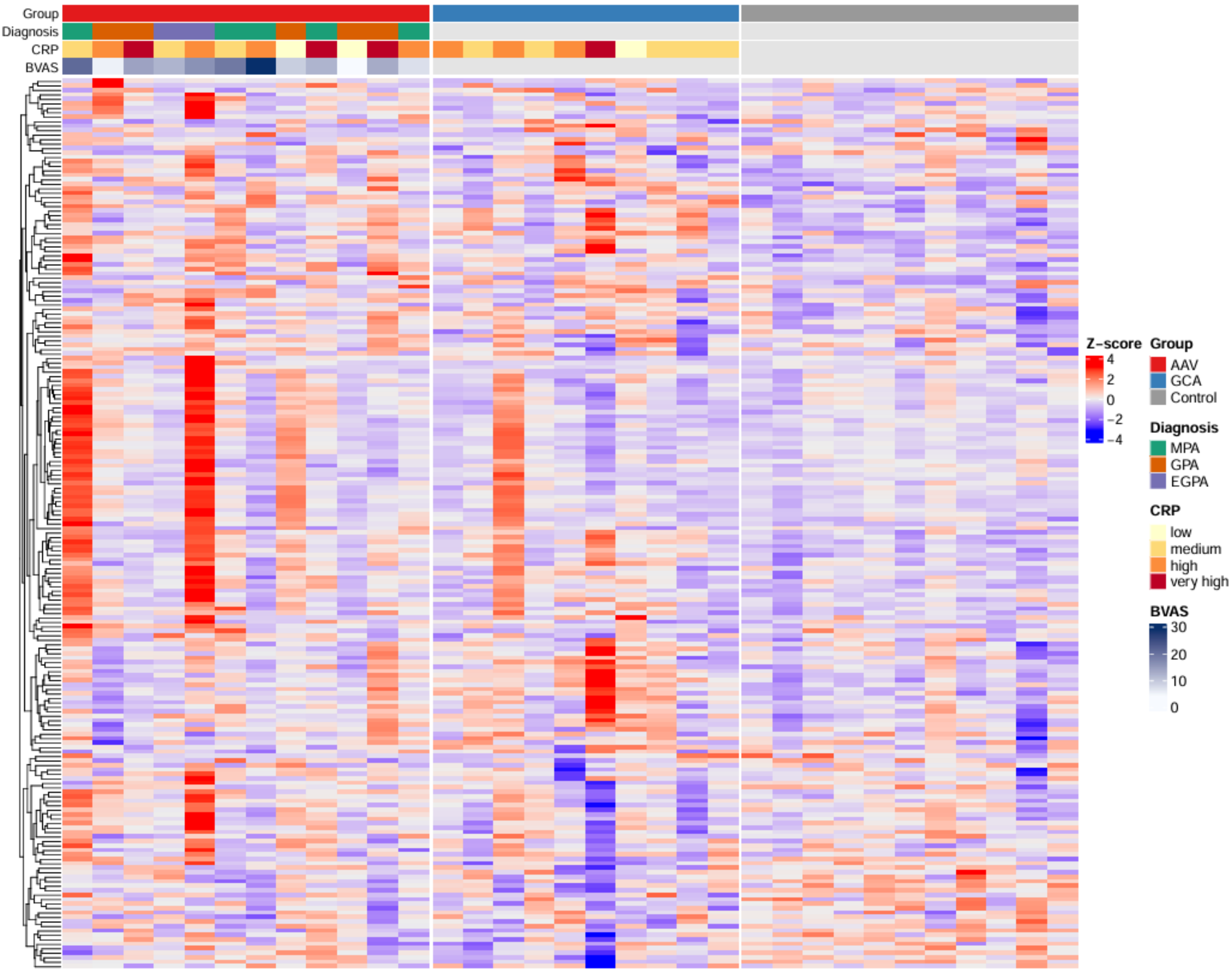

Figure legend Supplementary Figure S5:

(A) Dot plot of enriched gene sets (MSigDB) in whole blood of patients with ANCA-associated vasculitis (AAV) compared to giant cell arteritis (GCA). (B and C) Heatmaps of DEG involved in (B) " IFNalpha response" and (C) "IFNgamma response" in the peripheral blood of patients with AAV, GCA and healthy controls. CRP: C-reactive protein, CRP-levels are defined as following: low <10mg/L, medium 11-49 mg/L, high 50-100mg/L, very high >100mg/L. BVAS: Bermingham Vasculitis Activity Score.

Supplementary Figure S5 following

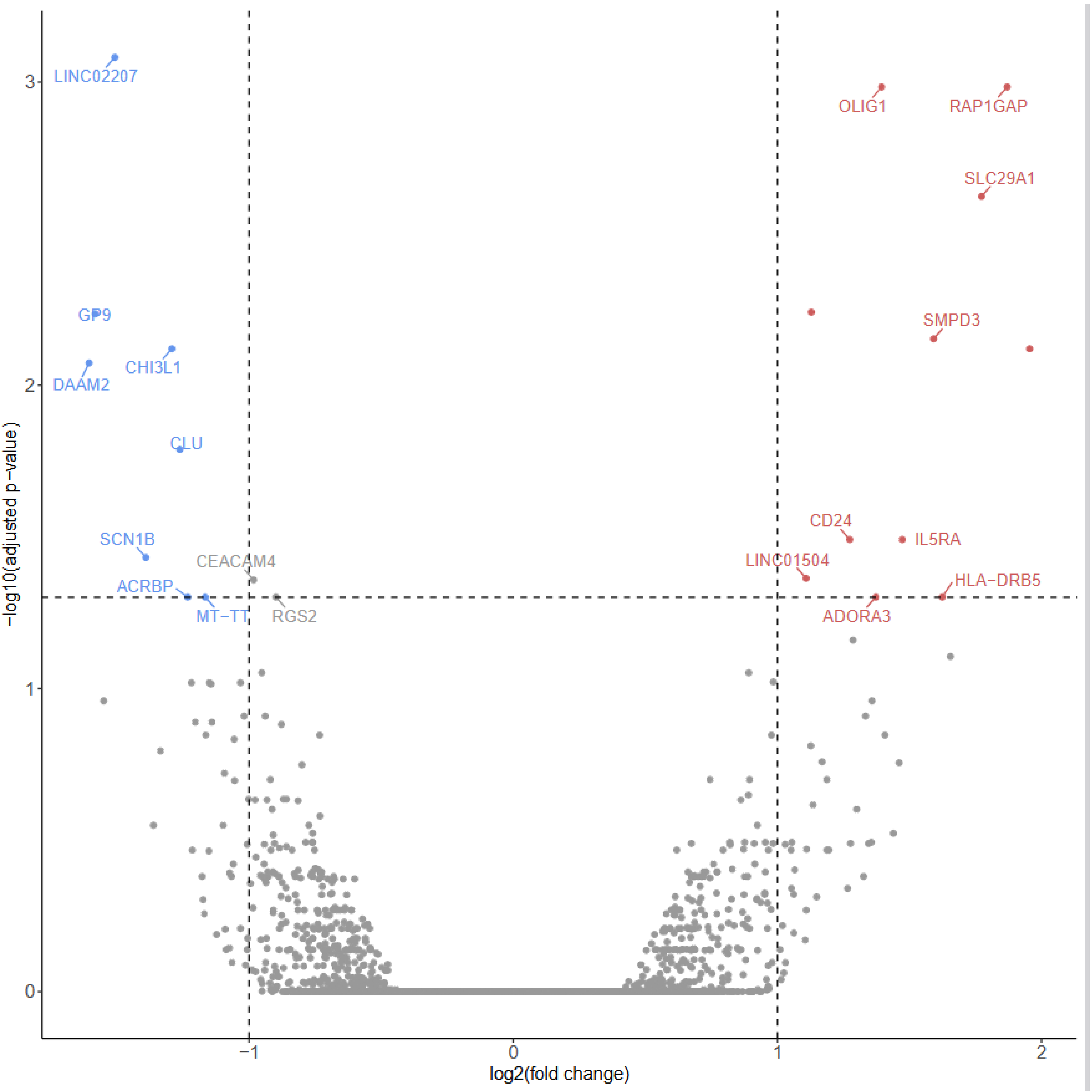

Figure legend of Supplementary Figure S6: Volcano plot showing 19 labeled differentially expressed genes (DEG) between ANCA-associated vasculitis and giant cell arteritis.

Supplementary Figure S6

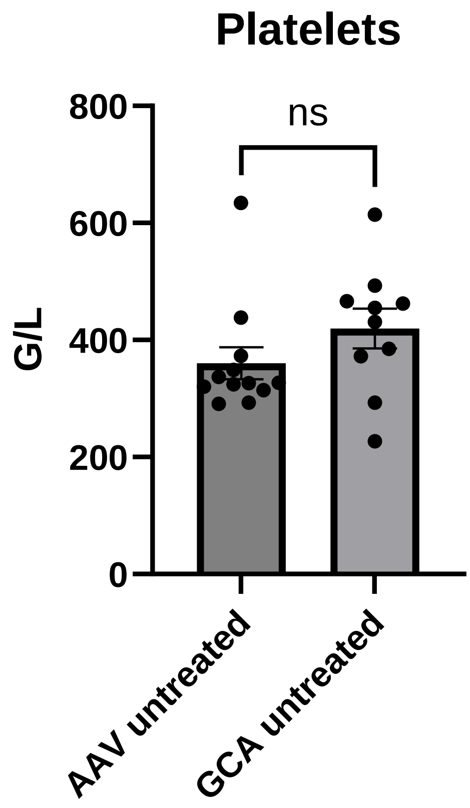

Figure legend Supplementary Figure S7: Dot plot of platelet counts in the peripheral blood of untreated giant cell arteritis (GCA) and ANCA-associated vasculitis (AAV) patients. Unpaired t-test between untreated AAV and LVV patients. Ns, not significant.

Supplementary Figure S7
