# Supplementary material for: Same, Same, but Different—Whole Blood Transcriptomes of ANCA-Associated Vasculitis and Giant Cell Arteritis
Source: Int J Mol Sci. 2026 Sep 24;27(19):8513. doi: 10.3390/ijms27198513 (PMC13664966; doi:10.3390/ijms27198513)
Supplement: Supplementary file 1 [file ijms-27-08513-s001.zip › Supplementary Table S1.pdf]

	HC	AAV	GCA	Difference between AAV and GCA (P-value)
n (%)	11 (100)	12 (100)	10 (100)	
Women n (%)	7 (63.6)	8 (66.7)	7 (70)	
Age (years, mean, min, max)	62.9 (26.0; 83.0)	51.4 (28.0;76.0)	71.5 (51.0; 86.0)	0.0011
Newly diagnosed		10	10	
Relapse		2	0	
**AVV-subtypes**				
MPA		6		
GPA		4		
EGPA		2		
Anti-MPO subtype		6		
Anti-PR3 subtype		4		
ANCA-negative		2		
**Clinical manifestations** of n patients according to BVAS (%):		**12 (100)**		
- General symptoms (fever, weight loss)		7		
- Cutaneous		1		
- Mouth ulcers		1		
- Ophthalmologic		0		
- Musculoskeletal		5		
- Ear, nose, throat		5		
- Pulmonary (Wheeze, nodules, infiltrate etc)		8		
- Cardiovascular		2		
- Gastrointestinal		2		
- Renal		5		
- Neurological		1		
BVAS score		12.5 (7.5;18.3)		
**Clinical manifestations** of n GCA patients (%):			**10 (100)**	
- General symptoms (fever, weight loss)			6	
- Cranial symptoms			8	
- Visual symptoms			4	
- Polymyalgia			4	
- Positive imaging (ultrasound, MRI or PET/ CT)			10	

**Clinical subtypes of GCA**				
- cGCA only			5	
- cGCA+LV-GCA			1	
- cGCA+LV-GCA+PMR			2	
- LV-GCA only			0	
- LV-GCA+PMR			2	
- PMR only			0	
**Immunosuppressive treatment**		0	0	
**Laboratory data**				
Anti-PR3 titers (IU/mL) Ref: <5IU/mL		46.9 (23.7 ; 89.6)		
Anti-MPO titers (IU/mL) Ref: <6 IU/mL		103.5 (63.7; 421.5)		
CRP (mg/L), Ref: <5mg/L		8.3 (4.9; 10.5)	23.0 (13.5; 72.0)	0.0012
ESR (µmol/L), Ref: 0-20mm/h		40.0 (32.5; 49.0)	58.0 (26.75; 93.0)	0.0889
Creatinine (µmol/L), Ref: 45-84 µmol/L		83.0 (66.0; 357.3)	69.0 (64.0; 99.3)	0.2892
Leukocytes (G/L), Ref: 3-10.5G/L		9.9 (7.45; 10.93)	8.19 (5.95;10.93)	0.4858
Neutrophils (n/mm^3), Ref: 1600-7400/mm^3		6.5 (5.5; 7.5)	6.1 (4.1; 9.0)	0.6744
Platelets (G/L) Ref: 150-450G/L		332.0 (231.0, 367.0)	443.0 (352.3; 472.8)	0.1104

Supplementary Table S1a: Patient characteristics of untreated vasculitis patients and healthy controls used for bulkRNAseq of whole blood.

	HC	AAV	GCA	Difference between AAV and GCA (P-value)
n (%)	20 (100)	9 (100)	10 (100)	
Women n (%)				
Age (years, mean, min, max)	56.6 (23.0 ; 83.0)	52.3 (28.0; 77.0)	71.1 (51.0; 86.0)	0.02
Newly diagnosed		3	10	
Relapse		6		

**AVV-subtypes**				
MPA		4		
GPA		5		
EGPA		0		
Anti-MPO subtype		4		
Anti-PR3 subtype		5		
ANCA-negative		0		
**Clinical manifestations** of n patients according to BVAS (%):		**9 (100)**		
- General symptoms (fever, weight loss)		4		
- Cutaneous		0		
- Mouth ulcers		0		
- Ophthalmologic		0		
- Musculoskeletal		2		
- Ear, nose, throat		3		
- Pulmonary (Wheeze, nodules, infiltrate etc)		0		
- Cardiovascular		0		
- Gastrointestinal		2		
- Renal		3		
- Neurological		0		
BVAS score		7.0 (6.0 ;12)		
**Clinical manifestations** of n GCA patients (%):			**10 (100)**	
- General symptoms (fever, weight loss)			6	
- Cranial symptoms			8	
- Visual symptoms			3	
- Polymyalgia			3	
- Positive imaging (ultrasound, MRI or PET/CT)			10	
**Clinical subtypes of GCA**				
- cGCA only			5	
- cGCA+LV-GCA			2	
- cGCA+LV-GCA+PMR			1	
- LV-GCA only			0	
- LV-GCA+PMR			2	
- PMR only			0	
**Glucocorticoid treatment**		0	0	
**Other immunosuppressive treatment**		4	0	
- Rituximab		4	0	
**Laboratory data**				

Anti-PR3 titers (IU/mL) Ref: <5IU/mL		28.7 (18.2; 97.8)		
Anti-MPO titers (IU/mL) Ref: <6 IU/mL		269.3 (108.8; 850.0)		
CRP (mg/L), Ref: <5mg/L		4.0 (1.5; 8.5)	23 (12.75; 72)	0.005
ESR (µmol/L), Ref: 0-20mm/h		21 (9.5; 42.5)	63 (25.5; 97)	0.04
Creatinine (µmol/L), Ref: 45-84 µmol/L		83 (67; 111)	73 (64; 99)	0.46
Leukocytes (G/L), Ref: 3-10.5G/L		7.3 (6.6; 10.4)	9.6 (7.1; 11.9)	0.36
Neutrophils (n/mm^3), Ref: 1600-7400/mm^3		5.5(1.0; 8.7)	7.1(4.8; 9.0)	0.4
Platelets (G/L) Ref: 150-450G/L		293 (216; 343)	445 (381; 472)	0.01

Supplementary Table S1b: Patient characteristics of GC-naïve vasculitis patients and healthy controls used for flow cytometry analysis.

Legend: AAV: ANCA-associated vasculitis, ANCA: anti-neutrophil cytoplasmic antibodies, anti-PR3: Proteinase 3 ANCA, anti-MPO: Myeloperoxidase ANCA, BVAS: Birmingham vasculitis activity score, cGCA: cranial giant cell arteriitis, CRP: C-reactive protein, EGPA: eosinophilic granulomatosis with polyangiitis, GPA: granulomatosis with polyangiitis, GCA: giant cell arteritis, HC: healthy controls, LV-GCA: large vessel giant cell arteriitis, MPA: microscopic polyangiitis, MRI: magnetic resonance imaging, PET/ CT: positron emission tomography/ computer tomography, PMR: polymyalgia rheumatica, Ref: Reference values; If not otherwise indicated values are displayed as median and range (25th percentile; 75th percentile).
