# Supplementary material for: Same, Same, but Different—Whole Blood Transcriptomes of ANCA-Associated Vasculitis and Giant Cell Arteritis
Source: Int J Mol Sci. 2026 Sep 24;27(19):8513. doi: 10.3390/ijms27198513 (PMC13664966; doi:10.3390/ijms27198513)
Supplement: Supplementary file 1 [file ijms-27-08513-s001.zip › Supplementary Table S2 AAV.HC.DEGResults.pdf]

Supplementary Table S2: Differentially expressed genes (DEG) from the whole blood transcriptome of ANCA-associated vasculitis (AAV) compared to healthy controls (HC)

ensembl	entrezgene	gene_biotype	chromosome	gene	log2FC	lfcSE	stat	pvalue	padj
ENSG00000110203	2352	protein_coding	11	FOLR3	1.79357744	0.26725135	6.71120068	1.9303E-11	2.4399E-07
ENSG00000118113	4317	protein_coding	11	MMP8	2.26844936	0.36775673	6.16834221	6.901E-10	4.3614E-06
ENSG00000076864	5909	protein_coding	1	RAP1GAP	1.9158661	0.3445701	5.56016357	2.6952E-08	0.00011356
ENSG00000169385	6036	protein_coding	14	RNASE2	1.48663995	0.28724993	5.1754232	2.2739E-07	0.00071857
ENSG00000168062	116071	protein_coding	11	BATF2	1.60755079	0.3177541	5.05910328	4.2123E-07	0.0008874
ENSG00000169397	6037	protein_coding	14	RNASE3	1.93212897	0.38154321	5.06398467	4.1058E-07	0.0008874
ENSG00000010030	51513	protein_coding	6	ETV7	1.78662231	0.36255381	4.92788172	8.3126E-07	0.00150102
ENSG00000150337	2209	protein_coding	1	FCGR1A	1.48431345	0.30519286	4.86352621	1.1531E-06	0.00182194
ENSG00000152766	118932	protein_coding	10	ANKRD22	1.5595518	0.32819782	4.75186526	2.0155E-06	0.00283064
ENSG00000189060	3005	protein_coding	22	H1-0	1.20567673	0.25891136	4.65671616	3.2129E-06	0.00369195
ENSG00000197561	1991	protein_coding	19	ELANE	1.82486853	0.39034019	4.67507207	2.9385E-06	0.00369195
ENSG00000134321	91543	protein_coding	2	RSAD2	1.8068504	0.39687941	4.55264331	5.2976E-06	0.00478298
ENSG00000134755	1824	protein_coding	18	DSC2	1.250628	0.27442404	4.55728289	5.182E-06	0.00478298
ENSG00000171860	719	protein_coding	12	C3AR1	1.14340264	0.24986223	4.5761324	4.7365E-06	0.00478298
ENSG00000115523	10578	protein_coding	2	GNLY	-1.19093795	0.26308924	-4.52674514	5.9899E-06	0.0050475
ENSG00000204936	57126	protein_coding	19	CD177	1.85190904	0.41239557	4.49061334	7.1018E-06	0.00528042
ENSG00000274536	115891964	lncRNA	X	MIR223HG	1.01220882	0.2253345	4.49202772	7.0548E-06	0.00528042
ENSG00000164047	820	protein_coding	3	CAMP	1.32222414	0.29611638	4.46521783	7.9987E-06	0.00561689
ENSG00000279814		protein_coding	10		1.46465136	0.33043212	4.43253323	9.3132E-06	0.00619575
ENSG00000079385	634	protein_coding	19	CEACAM1	1.15815323	0.26243275	4.41314289	1.0188E-05	0.00643886
ENSG00000104522	7264	protein_coding	8	GFUS	1.17747681	0.27023751	4.35719238	1.3174E-05	0.00792958
ENSG00000149131	710	protein_coding	11	SERPING1	1.36956627	0.31970146	4.28389114	1.8365E-05	0.01055169
ENSG00000238113					1.10077161	0.25758143	4.27348979	1.9244E-05	0.01057567
ENSG00000225067		pseudogene	19	RPL23AP2	-1.12447868	0.26769055	-4.20066632	2.6613E-05	0.01401621
ENSG00000012223	4057	protein_coding	3	LTF	1.61387811	0.388539	4.15370948	3.2713E-05	0.01531446
ENSG00000115602	9173	protein_coding	2	IL1RL1	1.42360158	0.3420668	4.16176488	3.158E-05	0.01531446
ENSG00000198821	919	protein_coding	1	CD247	-0.92778574	0.22313378	-4.15797985	3.2107E-05	0.01531446
ENSG00000180509	3753	protein_coding	21	KCNE1	1.2328635	0.30009767	4.10820752	3.9874E-05	0.01800034

ENSG00000161944	433	protein_coding	17	ASGR2	1.0321892	0.25208954	4.09453394	4.2302E-05	0.01809302
ENSG00000163568	9447	protein_coding	1	AIM2	1.11682469	0.27350088	4.08344099	4.4374E-05	0.01809302
ENSG00000243273	124909446	lncRNA	3		1.74786424	0.42788376	4.0849044	4.4095E-05	0.01809302
ENSG00000237943	439949	lncRNA	10	PRKCQ-AS1	-0.96016264	0.2363339	-4.06273763	4.8501E-05	0.01915771
ENSG00000186854					-0.96098927	0.23830827	-4.03254688	5.5176E-05	0.02113393
ENSG00000110693	55553	protein_coding	11	SOX6	1.22015763	0.30378845	4.01647147	5.9076E-05	0.02196238
ENSG00000144290	57282	protein_coding	2	SLC4A10	-1.41767783	0.35424362	-4.00198554	6.2813E-05	0.02205439
ENSG00000270972		lncRNA	11		1.28102965	0.31957173	4.0085825	6.1084E-05	0.02205439
ENSG00000038427	1462	protein_coding	5	VCAN	0.87969512	0.22125755	3.97588738	7.0117E-05	0.02387714
ENSG00000148346	3934	protein_coding	9	LCN2	1.51599384	0.38183369	3.97029879	7.1783E-05	0.02387714
ENSG00000047597	7504	protein_coding	X	XK	1.21376744	0.30820267	3.938212	8.2091E-05	0.02429213
ENSG00000092758	1299	protein_coding	20	COL9A3	1.13429191	0.28688122	3.95387301	7.6896E-05	0.02429213
ENSG00000096006	10321	protein_coding	6	CRISP3	1.42264544	0.36102495	3.94057378	8.1287E-05	0.02429213
ENSG00000156381	122416	protein_coding	14	ANKRD9	1.24360691	0.3145532	3.95356624	7.6995E-05	0.02429213
ENSG00000180667	55432	protein_coding	1	YOD1	1.06680702	0.27099609	3.93661399	8.2639E-05	0.02429213
ENSG00000181381	91351	protein_coding	4	DDX60L	1.01231852	0.2590485	3.90783396	9.3127E-05	0.02675292
ENSG00000122224	4063	protein_coding	1	LY9	-1.00300889	0.25702898	-3.90231829	9.5276E-05	0.0267619
ENSG00000205726	6453	protein_coding	21	ITSN1	1.03280831	0.26526932	3.89343299	9.8836E-05	0.02715828
ENSG00000137959	10964	protein_coding	1	IFI44L	1.55946684	0.40302136	3.86943969	0.00010909	0.02813967
ENSG00000157388	776	protein_coding	3	CACNA1D	1.32005093	0.34066975	3.8748698	0.00010668	0.02813967
ENSG00000289066		lncRNA	15		1.34061613	0.34643362	3.86976334	0.00010894	0.02813967
ENSG00000104918	56729	protein_coding	19	RETN	1.16774297	0.30382826	3.8434311	0.00012133	0.02949157
ENSG00000119950	4601	protein_coding	10	MXI1	1.01507943	0.26405006	3.84426891	0.00012091	0.02949157
ENSG00000138496	83666	protein_coding	3	PARP9	0.9484558	0.24643648	3.84868263	0.00011875	0.02949157
ENSG00000110318	57562	protein_coding	11	CEP126	-1.05615302	0.27598683	-3.826824	0.00012981	0.03068919
ENSG00000122490	80148	protein_coding	18	SLC66A2	0.88907833	0.23247729	3.82436631	0.00013111	0.03068919
ENSG00000272398	100133941	protein_coding	6	CD24	1.09032097	0.28694088	3.79981048	0.00014481	0.03327923
ENSG00000152229	9050	protein_coding	18	PSTPIP2	1.01079987	0.26640612	3.79420668	0.00014812	0.03343196
ENSG00000133069	9911	protein_coding	1	TMCC2	1.12578935	0.29711488	3.78907085	0.00015121	0.03353188
ENSG00000152484	219333	protein_coding	13	USP12	0.92535333	0.24473144	3.78109712	0.00015614	0.03402748
ENSG00000163840	151636	protein_coding	3	DTX3L	0.86878171	0.23059363	3.7675876	0.00016483	0.03531331

ENSG00000124107	6590	protein_coding	20	SLPI	1.1723955	0.3119797	3.75792243	0.00017133	0.03564012
ENSG00000239961	23547	protein_coding	19	LILRA4	-1.25964837	0.33528491	-3.75694916	0.000172	0.03564012
ENSG00000165949	3429	protein_coding	14	IFI27	1.59075416	0.42520795	3.74112043	0.0001832	0.03722087
ENSG00000181788	6478	protein_coding	3	SIAH2	1.04418834	0.27934671	3.73796546	0.00018552	0.03722087
ENSG00000154027	26289	protein_coding	1	AK5	-1.23206034	0.33055407	-3.72725807	0.00019357	0.03774019
ENSG00000196778	390036	protein_coding	11	OR52K1	1.13918589	0.30568991	3.72660616	0.00019408	0.03774019
ENSG00000101425	671	protein_coding	20	BPI	1.23522336	0.33365098	3.70214219	0.00021379	0.03783202
ENSG00000106070	2887	protein_coding	7	GRB10	1.22311454	0.33014521	3.7047775	0.00021158	0.03783202
ENSG00000111716	3945	protein_coding	12	LDHB	-0.82878006	0.22281562	-3.71957785	0.00019956	0.03783202
ENSG00000117318	3399	protein_coding	1	ID3	-0.96106989	0.25987459	-3.69820643	0.00021713	0.03783202
ENSG00000162894	9214	protein_coding	1	FCMR	-0.83796919	0.22545003	-3.71687322	0.0002017	0.03783202
ENSG00000183691	9241	protein_coding	17	NOG	-0.99874506	0.27017816	-3.69661662	0.00021849	0.03783202
ENSG00000198574	117157	protein_coding	1	SH2D1B	-1.03163847	0.27866817	-3.70203194	0.00021388	0.03783202
ENSG00000198829	56670	protein_coding	3	SUCNR1	1.26460142	0.3405681	3.71321161	0.00020465	0.03783202
ENSG00000134243	6272	protein_coding	1	SORT1	0.99277497	0.26952009	3.68349158	0.00023006	0.03877293
ENSG00000282988		protein_coding	6		1.00081611	0.27155787	3.68546167	0.00022829	0.03877293
ENSG00000170989	1901	protein_coding	1	S1PR1	-0.86846403	0.23627269	-3.67568514	0.00023721	0.03945209
ENSG00000137078	27240	protein_coding	9	SIT1	-0.85687101	0.23429886	-3.65717111	0.00025501	0.04186206
ENSG00000095932	284422	protein_coding	19	SMIM24	1.07631648	0.29531365	3.64465537	0.00026775	0.04199039
ENSG00000120217	29126	protein_coding	9	CD274	1.04853022	0.28709119	3.65225496	0.00025995	0.04199039
ENSG00000160883	3101	protein_coding	5	HK3	0.87357269	0.2393486	3.64979234	0.00026245	0.04199039
ENSG00000188313	5359	protein_coding	3	PLSCR1	1.01077091	0.27742691	3.64337727	0.00026908	0.04199039
ENSG00000123609	9111	protein_coding	2	NMI	0.81342546	0.22381151	3.63442188	0.0002786	0.04275633
ENSG00000161040	222235	protein_coding	7	FBXL13	1.05089602	0.28930884	3.6324366	0.00028076	0.04275633
ENSG00000133816	9645	protein_coding	11	MICAL2	0.86598866	0.23931846	3.61856193	0.00029624	0.04417986
ENSG00000183347	163351	protein_coding	1	GBP6	1.37307662	0.37953155	3.61781943	0.0002971	0.04417986
ENSG00000055332	5610	protein_coding	2	EIF2AK2	1.01591881	0.28155238	3.60827642	0.00030824	0.04478308
ENSG00000168528	347735	protein_coding	1	SERINC2	1.26914807	0.35148493	3.6108179	0.00030523	0.04478308
ENSG00000087253	54947	protein_coding	16	LPCAT2	0.87730803	0.24388121	3.59727596	0.00032157	0.04566978
ENSG00000122420	5737	protein_coding	1	PTGFR	1.34845656	0.37474996	3.59828343	0.00032032	0.04566978
ENSG00000035664	23604	protein_coding	15	DAPK2	0.86009299	0.24090295	3.57028834	0.00035659	0.04684552

ENSG00000065675	5588	protein_coding	10	PRKCQ	-0.81568865	0.2280646	-3.5765684	0.00034813	0.04684552
ENSG00000081059	6932	protein_coding	5	TCF7	-0.88607591	0.24744645	-3.58087947	0.00034244	0.04684552
ENSG00000145685	10184	protein_coding	5	LHFPL2	0.95594236	0.26742066	3.57467659	0.00035066	0.04684552
ENSG00000155659	11326	protein_coding	X	VSIG4	1.35363338	0.37911287	3.57052869	0.00035626	0.04684552
ENSG00000165029	19	protein_coding	9	ABCA1	1.04403276	0.29237833	3.57082807	0.00035585	0.04684552
ENSG00000185339	6948	protein_coding	22	TCN2	1.11666711	0.31211676	3.5777223	0.0003466	0.04684552
ENSG00000213722	23564	protein_coding	6	DDAH2	0.82344867	0.23077674	3.56816152	0.00035949	0.04684552
ENSG00000143162	8804	protein_coding	1	CREG1	0.87241735	0.24538444	3.5553083	0.00037754	0.04869446
ENSG00000116717	1647	protein_coding	1	GADD45A	1.04859417	0.29606994	3.54171107	0.00039754	0.04926386
ENSG00000126353	1236	protein_coding	17	CCR7	-0.88298542	0.24898983	-3.54627106	0.00039072	0.04926386
ENSG00000164821	1669	protein_coding	8	DEFA4	1.38111921	0.3899391	3.54188439	0.00039728	0.04926386
ENSG00000198034	6191	protein_coding	X	RPS4X	-0.73182154	0.20635635	-3.54639707	0.00039054	0.04926386
ENSG00000197766	1675	protein_coding	19	CFD	0.99937217	0.28274953	3.53447859	0.00040858	0.04965825
ENSG00000211772		protein_coding	7	TRBC2	-0.8107615	0.22926244	-3.53639042	0.00040563	0.04965825
