# Supplementary material for: Same, Same, but Different—Whole Blood Transcriptomes of ANCA-Associated Vasculitis and Giant Cell Arteritis
Source: Int J Mol Sci. 2026 Sep 24;27(19):8513. doi: 10.3390/ijms27198513 (PMC13664966; doi:10.3390/ijms27198513)
Supplement: Supplementary file 1 [file ijms-27-08513-s001.zip › Supplementary Table S3 GCA.HC.DEGResults.pdf]

Supplementary Table S3: Differentially expressed genes (DEG) from the whole blood transcriptome of giant cell arteritis (GCA) compared to healthy controls (HC)

ensembl	entrezgene	gene_biotype	chromosome	gene	log2FC	lfcSE	stat	pvalue	padj
ENSG00000258476	101927129	lncRNA	15	LINC02207	1.97989327	0.28220105	7.01589612	2.2848E-12	2.2512E-08
ENSG00000146122	23500	protein_coding	6	DAAM2	2.37999812	0.36042577	6.60329621	4.0211E-11	1.981E-07
ENSG00000120885	1191	protein_coding	8	CLU	1.83407185	0.29074549	6.30816954	2.8235E-10	9.2735E-07
ENSG00000101335	10398	protein_coding	20	MYL9	2.00146258	0.32082957	6.23839805	4.4207E-10	1.0889E-06
ENSG00000259207	3690	protein_coding	17	ITGB3	1.78324171	0.30059612	5.93235096	2.9863E-09	5.8848E-06
ENSG00000159674	10417	protein_coding	4	SPON2	-1.36149766	0.23147783	-5.88176269	4.0592E-09	6.6659E-06
ENSG00000103196	83716	protein_coding	16	CRISPLD2	1.35917547	0.23945088	5.6762184	1.377E-08	1.9383E-05
ENSG00000005961	3674	protein_coding	17	ITGA2B	1.74497976	0.30871357	5.65242315	1.582E-08	1.9484E-05
ENSG00000143297	83416	protein_coding	1	FCRL5	-1.51327995	0.26947817	-5.61559375	1.9589E-08	2.1445E-05
ENSG00000166523	26253	protein_coding	12	CLEC4E	1.31010823	0.24409592	5.3671862	7.9974E-08	6.9727E-05
ENSG00000167460	7171	protein_coding	19	TPM4	1.09107935	0.20369837	5.35634808	8.4921E-08	6.9727E-05
ENSG00000182885	222487	protein_coding	16	ADGRG3	1.46890529	0.27320312	5.3766051	7.5903E-08	6.9727E-05
ENSG00000100385	3560	protein_coding	22	IL2RB	-1.26976626	0.23790069	-5.33737955	9.43E-08	7.1472E-05
ENSG00000204420	80739	protein_coding	6	MPIG6B	1.5506782	0.29615592	5.23601959	1.6408E-07	0.00011547
ENSG00000137801	7057	protein_coding	15	THBS1	1.63689253	0.31416575	5.21028325	1.8855E-07	0.00012385
ENSG00000127507	30817	protein_coding	19	ADGRE2	1.32005034	0.25966306	5.08370484	3.7014E-07	0.00022794
ENSG00000105352	1089	protein_coding	19	CEACAM4	1.21389527	0.24143947	5.02774158	4.9629E-07	0.0002677
ENSG00000115523	10578	protein_coding	2	GNLY	-1.39935073	0.27773421	-5.03845289	4.6931E-07	0.0002677
ENSG00000204936	57126	protein_coding	19	CD177	2.12768733	0.42382665	5.02018292	5.1622E-07	0.0002677
ENSG00000113140	6678	protein_coding	5	SPARC	1.4157631	0.28542281	4.96023105	7.0409E-07	0.00034687
ENSG00000289927		lncRNA	21		1.57111837	0.31758904	4.947017	7.5359E-07	0.00035358
ENSG00000005249	5577	protein_coding	7	PRKAR2B	1.37257702	0.27984624	4.90475419	9.3544E-07	0.00040074
ENSG00000161911	340205	protein_coding	6	TREML1	1.51715352	0.30885353	4.9122104	9.0055E-07	0.00040074
ENSG00000103569	366	protein_coding	15	AQP9	1.1784453	0.24391665	4.83134424	1.3561E-06	0.00055675
ENSG00000254470	91056	protein_coding	11	AP5B1	1.17165718	0.24346415	4.81244242	1.491E-06	0.00058762
ENSG00000157168	3084	protein_coding	8	NRG1	1.48511554	0.30937491	4.8003749	1.5837E-06	0.00060016
ENSG00000118113	4317	protein_coding	11	MMP8	1.83278494	0.38432538	4.76883663	1.8529E-06	0.0006323
ENSG00000159339	23569	protein_coding	1	PADI4	1.19127263	0.24984959	4.76795915	1.861E-06	0.0006323

ENSG00000197249	5265	protein_coding	14	SERPINA1	1.16163518	0.24352477	4.7700904	1.8414E-06	0.0006323
ENSG00000155659	11326	protein_coding	X	VSIG4	1.85514314	0.3903259	4.75280569	2.0061E-06	0.00065888
ENSG00000135636	8291	protein_coding	2	DYSF	1.36434204	0.2878469	4.73981844	2.1391E-06	0.00067989
ENSG00000122786	800	protein_coding	7	CALD1	1.51475884	0.32134653	4.71378623	2.4316E-06	0.00074869
ENSG00000115590	7850	protein_coding	2	IL1R2	1.38422667	0.29531382	4.68730739	2.7682E-06	0.00080222
ENSG00000198223	1438	protein_coding	X	CSF2RA	1.13539191	0.24205574	4.69062165	2.7238E-06	0.00080222
ENSG00000143595	89872	protein_coding	1	AQP10	1.60614273	0.34373855	4.67257081	2.9745E-06	0.00083737
ENSG00000142046	641649	protein_coding	19	TMEM91	1.13514276	0.24607903	4.61291957	3.9705E-06	0.00108671
ENSG00000160991	80228	protein_coding	7	ORAI2	1.01756259	0.22215536	4.58040972	4.6407E-06	0.00123579
ENSG00000008438	8993	protein_coding	19	PGLYRP1	1.40265559	0.3071134	4.56722363	4.9423E-06	0.00127769
ENSG00000182022	51363	protein_coding	10	CHST15	1.09265581	0.23949171	4.56239516	5.0573E-06	0.00127769
ENSG00000124491	2162	protein_coding	6	F13A1	1.25969293	0.2765599	4.55486478	5.2419E-06	0.00129122
ENSG00000132704	79368	protein_coding	1	FCRL2	-1.37884516	0.30415407	-4.53337735	5.8048E-06	0.0013737
ENSG00000288882		lncRNA	7		1.47993463	0.32658565	4.53153601	5.8556E-06	0.0013737
ENSG00000088726	55287	protein_coding	3	TMEM40	1.39596572	0.30918277	4.51501781	6.3311E-06	0.00145072
ENSG00000018280	6556	protein_coding	2	SLC11A1	1.22033676	0.27278236	4.47366443	7.689E-06	0.00163196
ENSG00000064601	5476	protein_coding	20	CTSA	1.11799772	0.25127813	4.449244	8.6173E-06	0.00163196
ENSG00000101162	81027	protein_coding	20	TUBB1	1.24187252	0.27878906	4.45452391	8.408E-06	0.00163196
ENSG00000142405	91662	protein_coding	19	NLRP12	1.05279123	0.23695213	4.44305448	8.8691E-06	0.00163196
ENSG00000162614	91624	protein_coding	1	NEXN	1.38625975	0.31008817	4.47053409	7.8025E-06	0.00163196
ENSG00000163736	5473	protein_coding	4	PPBP	1.25366946	0.28173689	4.44978813	8.5955E-06	0.00163196
ENSG00000166963	4130	protein_coding	15	MAP1A	1.48249854	0.33176031	4.46858316	7.8739E-06	0.00163196
ENSG00000169704	2815	protein_coding	3	GP9	1.50195599	0.33786177	4.44547476	8.7698E-06	0.00163196
ENSG00000184730	55911	protein_coding	16	APOBR	1.03881447	0.2339017	4.44124368	8.944E-06	0.00163196
ENSG00000196923	9260	protein_coding	5	PDLIM7	1.13984971	0.25502208	4.46961179	7.8362E-06	0.00163196
ENSG00000198574	117157	protein_coding	1	SH2D1B	-1.32357989	0.29713537	-4.45446766	8.4102E-06	0.00163196
ENSG00000167434	762	protein_coding	17	CA4	1.43569383	0.32357803	4.43693235	9.125E-06	0.0016347
ENSG00000128383					1.18446308	0.26769623	4.42465361	9.6597E-06	0.00164099
ENSG00000141574	6398	protein_coding	17	SECTM1	1.06435449	0.24046974	4.42614737	9.5931E-06	0.00164099
ENSG00000168916	57507	protein_coding	5	ZNF608	1.57277853	0.35527418	4.42694303	9.5578E-06	0.00164099
ENSG00000197081	3482	protein_coding	6	IGF2R	1.0304823	0.2334028	4.41503833	1.0099E-05	0.00168657

ENSG00000005379	9256	protein_coding	17	TSPOAP1	-1.31325305	0.29920895	-4.38908341	1.1383E-05	0.00186575
ENSG00000150045	51348	protein_coding	12	KLRF1	-1.20835232	0.27550855	-4.38589763	1.1551E-05	0.00186575
ENSG00000203710	1378	protein_coding	1	CR1	1.20832418	0.27623626	4.3742418	1.2186E-05	0.00193652
ENSG00000117115	11240	protein_coding	1	PADI2	1.14826067	0.26302077	4.36566538	1.2674E-05	0.00198212
ENSG00000061918	2983	protein_coding	4	GUCY1B1	1.25874995	0.28965487	4.34568882	1.3884E-05	0.00213747
ENSG00000204054	124900275	lncRNA	9	LINC00963	1.12149511	0.25837756	4.34052836	1.4214E-05	0.00215463
ENSG00000047648	395	protein_coding	X	ARHGAP6	1.3425113	0.31012815	4.3288921	1.4986E-05	0.00223725
ENSG00000137076	7094	protein_coding	9	TLN1	1.01929574	0.23674755	4.30541206	1.6668E-05	0.00245112
ENSG00000197324	26020	protein_coding	14	LRP10	0.99434442	0.23228215	4.28076119	1.8626E-05	0.00269878
ENSG00000269404	6689	protein_coding	19	SPIB	-1.19875241	0.28026382	-4.27722859	1.8923E-05	0.00270221
ENSG00000060982	586	protein_coding	12	BCAT1	1.30560867	0.30732275	4.24833069	2.1537E-05	0.00303148
ENSG00000255197	127138868	lncRNA	11	SLC39A13-AS1	1.03345436	0.2439605	4.23615445	2.2738E-05	0.00315546
ENSG00000110203	2352	protein_coding	11	FOLR3	1.19134058	0.28157003	4.23106314	2.3259E-05	0.00318292
ENSG00000095932	284422	protein_coding	19	SMIM24	1.30735361	0.31106371	4.20284833	2.6358E-05	0.0034366
ENSG00000125735	8740	protein_coding	19	TNFSF14	0.96234148	0.22899567	4.20244409	2.6405E-05	0.0034366
ENSG00000168497	8436	protein_coding	2	CAVIN2	1.13779252	0.27078787	4.20178545	2.6482E-05	0.0034366
ENSG00000289310		lncRNA	12		1.20203818	0.28609307	4.20156347	2.6508E-05	0.0034366
ENSG00000211829		protein_coding	14	TRDC	-1.27712304	0.30425387	-4.19755732	2.6981E-05	0.00345251
ENSG00000144645	114884	protein_coding	3	OSBPL10	-1.17785033	0.28081424	-4.19441099	2.7358E-05	0.0034559
ENSG00000183019	199675	protein_coding	19	MCEMP1	1.32084164	0.31577249	4.18289019	2.8783E-05	0.00358981
ENSG00000111644	84519	protein_coding	12	ACRBP	1.29615542	0.31034475	4.17650184	2.9603E-05	0.00360092
ENSG00000122490	80148	protein_coding	18	SLC66A2	0.99735421	0.23867214	4.17876261	2.931E-05	0.00360092
ENSG00000057704	57458	protein_coding	12	TMCC3	0.97186262	0.23290689	4.1727517	3.0094E-05	0.00361609
ENSG00000288819					1.21454173	0.29126743	4.16985085	3.048E-05	0.0036183
ENSG00000187699	84281	protein_coding	2	C2orf88	1.17505998	0.28258284	4.15828497	3.2065E-05	0.0037611
ENSG00000128245	7533	protein_coding	22	YWHAH	1.03000686	0.24809638	4.15164005	3.301E-05	0.00382435
ENSG00000133818	22800	protein_coding	11	RRAS2	-1.31189986	0.31660027	-4.14371042	3.4173E-05	0.00382435
ENSG00000148346	3934	protein_coding	9	LCN2	1.64182009	0.3964569	4.14123228	3.4544E-05	0.00382435
ENSG00000163993	6286	protein_coding	4	S100P	1.14274487	0.27578324	4.14363429	3.4184E-05	0.00382435
ENSG00000198734	2153	protein_coding	1	F5	1.14654312	0.27668377	4.14387563	3.4148E-05	0.00382435
ENSG00000198821	919	protein_coding	1	CD247	-0.93687917	0.22715242	-4.12445167	3.7162E-05	0.0040684

ENSG00000102265	7076	protein_coding	X	TIMP1	1.03419998	0.25119448	4.11712863	3.8362E-05	0.0041412
ENSG00000116991	57568	protein_coding	1	SIPA1L2	1.25195057	0.30421847	4.115301	3.8667E-05	0.0041412
ENSG00000257335	8972	protein_coding	7	MGAM	1.2087055	0.29406572	4.11032432	3.951E-05	0.00418598
ENSG00000184500	5627	protein_coding	3	PROS1	1.47852261	0.36074294	4.09854897	4.1575E-05	0.00435784
ENSG00000124588	4835	protein_coding	6	NQO2	1.09163391	0.26665345	4.09383011	4.2431E-05	0.00440072
ENSG00000110080	6484	protein_coding	11	ST3GAL4	1.25917506	0.308591	4.08040111	4.4958E-05	0.00461429
ENSG00000073861	30009	protein_coding	17	TBX21	-1.09537117	0.26926902	-4.06794354	4.743E-05	0.0048178
ENSG00000133816	9645	protein_coding	11	MICAL2	1.00344444	0.24770075	4.05103507	5.0992E-05	0.00512673
ENSG00000134463	79746	protein_coding	10	ECHDC3	1.41495688	0.35004036	4.04226778	5.2937E-05	0.0051634
ENSG00000151948	144423	protein_coding	12	GLT1D1	1.05672098	0.26156338	4.04001882	5.3447E-05	0.0051634
ENSG00000158825	978	protein_coding	1	CDA	1.13423341	0.28074145	4.04013519	5.342E-05	0.0051634
ENSG00000274536	115891964	lncRNA	X	MIR223HG	0.92801265	0.2297064	4.03999477	5.3452E-05	0.0051634
ENSG00000118689	2309	protein_coding	6	FOXO3	1.06846515	0.26486218	4.03404198	5.4826E-05	0.00523918
ENSG00000125810	22918	protein_coding	20	CD93	0.89544551	0.22233072	4.02753834	5.6364E-05	0.00523918
ENSG00000142657	5226	protein_coding	1	PGD	1.06598747	0.26458073	4.02896871	5.6022E-05	0.00523918
ENSG00000164047	820	protein_coding	3	CAMP	1.26082598	0.3129402	4.02896783	5.6022E-05	0.00523918
ENSG00000150337	2209	protein_coding	1	FCGR1A	1.29866276	0.32263116	4.02522421	5.6921E-05	0.00524152
ENSG00000177455	930	protein_coding	16	CD19	-1.06364295	0.26519711	-4.01076364	6.0523E-05	0.00552157
ENSG00000035664	23604	protein_coding	15	DAPK2	0.99695774	0.24934986	3.99822863	6.3818E-05	0.00564489
ENSG00000169902	8460	protein_coding	7	TPST1	1.29547663	0.32383489	4.00042323	6.3229E-05	0.00564489
ENSG00000268500	8778	protein_coding	19	SIGLEC5	1.08506745	0.2716175	3.99483636	6.4739E-05	0.00564489
ENSG00000272669		lncRNA	22		1.23839621	0.30990295	3.99607758	6.4401E-05	0.00564489
ENSG00000288869	107985770	lncRNA	2	SMANTIS	1.20938665	0.30212426	4.00294454	6.2559E-05	0.00564489
ENSG00000012223	4057	protein_coding	3	LTF	1.60735614	0.40263305	3.99211181	6.5487E-05	0.00564831
ENSG00000204577					0.96564283	0.24198336	3.9905341	6.5925E-05	0.00564831
ENSG00000012232	2137	protein_coding	8	EXTL3	0.97469553	0.24472208	3.98286716	6.8089E-05	0.00568542
ENSG00000186431	2204	protein_coding	19	FCAR	1.23667055	0.31046734	3.98325496	6.7978E-05	0.00568542
ENSG00000282988		protein_coding	6		1.13971619	0.28599289	3.98512075	6.7446E-05	0.00568542
ENSG00000197766	1675	protein_coding	19	CFD	1.18815111	0.2990789	3.97270116	7.1062E-05	0.00588383
ENSG00000096063	6732	protein_coding	6	SRPK1	1.04164089	0.26267384	3.96552955	7.3233E-05	0.00601305
ENSG00000021355	1992	protein_coding	6	SERPINB1	0.91982879	0.23226495	3.96025658	7.4869E-05	0.00609659

ENSG00000102010	660	protein_coding	X	BMX	1.23466398	0.3125311	3.95053152	7.7978E-05	0.00629767
ENSG00000003436	7035	protein_coding	2	TFPI	1.35983558	0.34506682	3.9407891	8.1214E-05	0.0065057
ENSG00000149218	23052	protein_coding	11	ENDOD1	1.04177843	0.2645249	3.93830001	8.2061E-05	0.00652054
ENSG00000169756	3987	protein_coding	2	LIMS1	0.89296519	0.22731422	3.92832967	8.5538E-05	0.00674244
ENSG00000133048	1116	protein_coding	1	CHI3L1	1.10728407	0.28331986	3.90824722	9.2968E-05	0.00724566
ENSG00000138772	306	protein_coding	4	ANXA3	1.30062017	0.33288242	3.90714583	9.3393E-05	0.00724566
ENSG00000156738	931	protein_coding	11	MS4A1	-1.02195052	0.26172934	-3.90460817	9.4378E-05	0.00726491
ENSG00000198478	83699	protein_coding	6	SH3BGRL2	1.2062202	0.30984861	3.89293406	9.9039E-05	0.00756459
ENSG00000163754	2992	protein_coding	3	GYG1	1.09566615	0.28165064	3.89016032	0.00010018	0.00759272
ENSG00000104805	4924	protein_coding	19	NUCB1	0.94740754	0.24368884	3.88777568	0.00010117	0.00760915
ENSG00000146376	93663	protein_coding	6	ARHGAP18	0.93264729	0.24064494	3.87561572	0.00010636	0.00793879
ENSG00000163803	151056	protein_coding	2	PLB1	1.1421997	0.29524868	3.86860226	0.00010946	0.00810917
ENSG00000160856	115352	protein_coding	1	FCRL3	-1.00322228	0.26020373	-3.85552621	0.00011548	0.0084913
ENSG00000197405	728	protein_coding	19	C5AR1	0.93020951	0.24179396	3.84711641	0.00011952	0.00868184
ENSG00000278828	8357	protein_coding	6	H3C10	1.17692536	0.3059759	3.84646422	0.00011983	0.00868184
ENSG00000106070	2887	protein_coding	7	GRB10	1.33240912	0.34661793	3.84402826	0.00012103	0.00870452
ENSG00000165115	55582	protein_coding	9	KIF27	0.98230636	0.25622231	3.833805	0.00012618	0.0090088
ENSG00000170345	2353	protein_coding	14	FOS	1.08772323	0.28433861	3.82545028	0.00013053	0.00925284
ENSG00000146094	79930	protein_coding	5	DOK3	1.05208763	0.27517113	3.82339394	0.00013163	0.00926374
ENSG00000104522	7264	protein_coding	8	GFUS	1.08645563	0.28477597	3.81512398	0.00013611	0.00951161
ENSG00000173210	22885	protein_coding	5	ABLIM3	1.25001063	0.32805987	3.81031254	0.00013879	0.00956572
ENSG00000287644		lncRNA	17		1.03767472	0.27233824	3.81024243	0.00013883	0.00956572
ENSG00000140416	7168	protein_coding	15	TPM1	1.05295966	0.2765481	3.80751007	0.00014037	0.00960483
ENSG00000213903	1241	protein_coding	14	LTB4R	0.9566568	0.25137089	3.8057581	0.00014137	0.00960637
ENSG00000185052	57419	protein_coding	20	SLC24A3	1.28565563	0.33926893	3.78948832	0.00015096	0.01018759
ENSG00000065534	4638	protein_coding	3	MYLK	1.16537521	0.30768031	3.78761708	0.0001521	0.01019477
ENSG00000007237	8522	protein_coding	17	GAS7	1.09501275	0.28961477	3.78092854	0.00015624	0.01040187
ENSG00000230257	58160	lncRNA	7	NFE4	1.06748238	0.28274885	3.77537298	0.00015977	0.01056509
ENSG00000188559	57186	protein_coding	20	RALGAPA2	1.01665342	0.26946444	3.77286669	0.00016138	0.01060068
ENSG00000113742	80315	protein_coding	5	CPEB4	0.90216997	0.23977361	3.76259073	0.00016816	0.01062117
ENSG00000134539	3824	protein_coding	12	KLRD1	-0.90368475	0.24015465	-3.76292844	0.00016794	0.01062117

ENSG00000163737	5196	protein_coding	4	PF4	1.17650675	0.31212949	3.76929056	0.00016371	0.01062117
ENSG00000166681	27018	protein_coding	X	BEX3	1.04412617	0.27707327	3.76841177	0.00016429	0.01062117
ENSG00000196735	3117	protein_coding	6	HLA-DQA1	-1.04704225	0.27814023	-3.76444008	0.00016692	0.01062117
ENSG00000250069		lncRNA	5		1.17630167	0.31239275	3.76545759	0.00016624	0.01062117
ENSG00000013016	30845	protein_coding	2	EHD3	1.20798937	0.32221505	3.74901592	0.00017753	0.01114141
ENSG00000116717	1647	protein_coding	1	GADD45A	1.16626889	0.31268714	3.72982689	0.00019161	0.01190025
ENSG00000275302	6351	protein_coding	17	CCL4	-1.0237145	0.27450818	-3.72926778	0.00019204	0.01190025
ENSG00000138185	953	protein_coding	10	ENTPD1	0.9395035	0.25242199	3.72195579	0.00019769	0.01212414
ENSG00000183023	6546	protein_coding	2	SLC8A1	1.07124816	0.28786058	3.72141325	0.00019811	0.01212414
ENSG00000111052	8825	protein_coding	12	LIN7A	0.97273633	0.26166761	3.7174503	0.00020124	0.01221659
ENSG00000280138		protein_coding	12		0.86444019	0.23260304	3.71637526	0.0002021	0.01221659
ENSG00000288932		lncRNA	2		0.97618323	0.26306344	3.71082817	0.00020658	0.01241131
ENSG00000140968	3394	protein_coding	16	IRF8	-0.85115079	0.22958635	-3.70732313	0.00020946	0.0124421
ENSG00000152213	115761	protein_coding	13	ARL11	0.85751604	0.23131525	3.7071314	0.00020962	0.0124421
ENSG00000251230	731424	lncRNA	4	MIR3945HG	1.20674739	0.32679993	3.69261831	0.00022196	0.01309546
ENSG00000181381	91351	protein_coding	4	DDX60L	1.00500396	0.27238688	3.68961953	0.00022459	0.01317192
ENSG00000038427	1462	protein_coding	5	VCAN	0.82711111	0.22426469	3.68810231	0.00022593	0.01317228
ENSG00000117318	3399	protein_coding	1	ID3	-1.01800047	0.27617923	-3.6860138	0.00022779	0.01320267
ENSG00000187116	353514	protein_coding	19	LILRA5	0.8640467	0.23460913	3.68292014	0.00023058	0.01328584
ENSG00000139132	121512	protein_coding	12	FGD4	0.97605005	0.26590135	3.67072249	0.00024187	0.01383123
ENSG00000196092	5079	protein_coding	9	PAX5	-0.95889483	0.26130172	-3.66968437	0.00024285	0.01383123
ENSG00000113749	3274	protein_coding	5	HRH2	1.0235343	0.27980986	3.65796364	0.00025423	0.01430955
ENSG00000116741	5997	protein_coding	1	RGS2	0.82098307	0.22439283	3.65868669	0.00025351	0.01430955
ENSG00000177575	9332	protein_coding	12	CD163	1.10159892	0.30126506	3.65657714	0.00025561	0.01430955
ENSG00000082014	6604	protein_coding	7	SMARCD3	1.03849733	0.28467483	3.64801252	0.00026428	0.01468225
ENSG00000119471	84263	protein_coding	9	HSDL2	0.83536071	0.22931981	3.64277606	0.00026971	0.01468225
ENSG00000127948	5447	protein_coding	7	POR	1.03526849	0.2841914	3.64285653	0.00026963	0.01468225
ENSG00000163191	6282	protein_coding	1	S100A11	0.89859216	0.24655988	3.64451904	0.00026789	0.01468225
ENSG00000273076		lncRNA	22		1.06937859	0.29353376	3.64311954	0.00026935	0.01468225
ENSG00000163735	6374	protein_coding	4	CXCL5	1.04911809	0.28812967	3.64113181	0.00027144	0.01469516
ENSG00000049323	4052	protein_coding	2	LTBP1	1.22560751	0.33673683	3.63965975	0.000273	0.01469866

ENSG00000033327	9846	protein_coding	11	GAB2	0.90264751	0.24826451	3.63582987	0.00027709	0.01474138
ENSG00000090376	11213	protein_coding	12	IRAK3	1.03366235	0.28424508	3.636518	0.00027635	0.01474138
ENSG00000125505	79143	protein_coding	19	MBOAT7	0.80398718	0.22119632	3.63472216	0.00027828	0.01474138
ENSG00000196209	284759	protein_coding	20	SIRPB2	0.96039962	0.2643929	3.63247136	0.00028072	0.01479108
ENSG00000030419	22807	protein_coding	2	IKZF2	-0.94844824	0.26122313	-3.63079734	0.00028255	0.01480817
ENSG00000066336	6688	protein_coding	11	SPI1	0.90745013	0.25022377	3.62655452	0.00028723	0.01489505
ENSG00000095585	29760	protein_coding	10	BLNK	-1.01033446	0.27853017	-3.62737886	0.00028631	0.01489505
ENSG00000113916	604	protein_coding	3	BCL6	0.96894198	0.26859863	3.60739736	0.00030928	0.0154689
ENSG00000127920	2791	protein_coding	7	GNG11	0.9720703	0.26909293	3.61239623	0.00030338	0.0154689
ENSG00000154930	84532	protein_coding	20	ACSS1	-0.78592064	0.21772928	-3.60962315	0.00030664	0.0154689
ENSG00000184557	9021	protein_coding	17	SOCS3	1.1867578	0.32864025	3.61111516	0.00030488	0.0154689
ENSG00000196549	4311	protein_coding	3	MME	0.89407468	0.24748014	3.61271285	0.00030301	0.0154689
ENSG00000198736	51734	protein_coding	16	MSRB1	0.86688852	0.23978434	3.61528419	0.00030002	0.0154689
ENSG00000289353		lncRNA	16		0.87704164	0.24307025	3.60818172	0.00030835	0.0154689
ENSG00000159335	5763	protein_coding	12	PTMS	1.04084534	0.28863963	3.6060375	0.00031091	0.01547161
ENSG00000100461	55147	protein_coding	14	RBM23	0.74399138	0.20667781	3.59976416	0.00031851	0.01553584
ENSG00000106780	1955	protein_coding	9	MEGF9	0.84520266	0.23461206	3.60255426	0.00031511	0.01553584
ENSG00000143162	8804	protein_coding	1	CREG1	0.91797165	0.25500631	3.59979977	0.00031846	0.01553584
ENSG00000205309	56953	protein_coding	17	NT5M	1.13964717	0.31648969	3.60089831	0.00031712	0.01553584
ENSG00000115594	3554	protein_coding	2	IL1R1	1.16485921	0.32378471	3.59763502	0.00032112	0.01558637
ENSG00000170458	929	protein_coding	5	CD14	0.76767076	0.21348218	3.59594781	0.00032321	0.01561085
ENSG00000158869	2207	protein_coding	1	FCER1G	0.87689005	0.24409498	3.5924133	0.00032763	0.015747
ENSG00000163221	6283	protein_coding	1	S100A12	1.00841882	0.28089808	3.58998119	0.0003307	0.0158175
ENSG00000264400		short_ncRNA	19	RN7SL491P	1.16640265	0.32515087	3.58726601	0.00033416	0.01590585
ENSG00000166527	338339	protein_coding	12	CLEC4D	1.10904464	0.30938123	3.58471851	0.00033744	0.01590821
ENSG00000185340	10634	protein_coding	22	GAS2L1	1.08201599	0.30176969	3.58556882	0.00033634	0.01590821
ENSG00000276900	138842261	lncRNA	12	AMN1-AS1	0.90953683	0.25437544	3.57556862	0.00034947	0.01637168
ENSG00000291236		lncRNA	2		0.93094611	0.26042454	3.57472505	0.0003506	0.01637168
ENSG00000172965	541471	lncRNA	2	MIR4435-2HG	0.88354266	0.24732552	3.57238772	0.00035374	0.01644062
ENSG00000288827		lncRNA	19		0.82513864	0.23147509	3.56469739	0.00036428	0.01685077
ENSG00000010327	23166	protein_coding	3	STAB1	0.97679979	0.27461836	3.55693547	0.00037521	0.01727527

ENSG00000107738	64115	protein_coding	10	VSIR	0.79890534	0.22509889	3.54913055	0.00038651	0.01771273
ENSG00000204304	5089	protein_coding	6	PBX2	1.05952631	0.29943211	3.53845255	0.00040248	0.01835941
ENSG00000040608	65078	protein_coding	22	RTN4R	-1.10894526	0.31396597	-3.5320556	0.00041234	0.01872264
ENSG00000134243	6272	protein_coding	1	SORT1	1.00312758	0.28416162	3.53013045	0.00041535	0.01877228
ENSG00000188313	5359	protein_coding	3	PLSCR1	1.03472156	0.29321128	3.52892816	0.00041725	0.01877228
ENSG00000140526	11057	protein_coding	15	ABHD2	0.74384178	0.21113724	3.52302497	0.00042665	0.01910815
ENSG00000088826	54498	protein_coding	20	SMOX	1.21919697	0.34633361	3.52029644	0.00043106	0.01918015
ENSG00000107679	59338	protein_coding	10	PLEKHA1	-0.79138949	0.2248503	-3.51962837	0.00043215	0.01918015
ENSG00000116815	965	protein_coding	1	CD58	0.79919096	0.22783845	3.50770884	0.00045198	0.01996922
ENSG00000124762	1026	protein_coding	6	CDKN1A	1.04198093	0.29715413	3.5065336	0.00045398	0.01996922
ENSG00000144677	10217	protein_coding	3	CTDSPL	1.1236873	0.32078693	3.50290856	0.00046021	0.02015299
ENSG00000137441	83888	protein_coding	4	FGFBP2	-1.00242542	0.28653606	-3.49842682	0.00046801	0.02033903
ENSG00000245532	283131	lncRNA	11	NEAT1	0.87721157	0.25076796	3.4981007	0.00046858	0.02033903
ENSG00000140332	7090	protein_coding	15	TLE3	0.84305776	0.24137163	3.49277896	0.00047802	0.0205986
ENSG00000281162		lncRNA	2	LINC01127	1.13132398	0.32394115	3.49237503	0.00047875	0.0205986
ENSG00000188677	29780	protein_coding	22	PARVB	1.01758941	0.29165216	3.48905154	0.00048474	0.02076574
ENSG00000197321	6840	protein_coding	10	SVIL	0.86470542	0.24809942	3.48531826	0.00049155	0.0209665
ENSG00000111058	79611	protein_coding	12	ACSS3	0.9062457	0.26021012	3.48274576	0.0004963	0.02107775
ENSG00000163625	23001	protein_coding	4	WDFY3	0.96161459	0.27625815	3.48085512	0.00049982	0.02113599
ENSG00000012124	933	protein_coding	19	CD22	-0.98098697	0.28243328	-3.47334053	0.00051402	0.0212329
ENSG00000102921	9683	protein_coding	16	N4BP1	0.79629844	0.22922669	3.47384689	0.00051305	0.0212329
ENSG00000103313	4210	protein_coding	16	MEFV	0.8686267	0.2501567	3.47233038	0.00051596	0.0212329
ENSG00000111817	29940	protein_coding	6	DSE	0.84480037	0.24300864	3.47642113	0.00050815	0.0212329
ENSG00000136869	7099	protein_coding	9	TLR4	0.90608481	0.26048673	3.47842983	0.00050436	0.0212329
ENSG00000165233	84270	protein_coding	9	CARD19	0.79963063	0.23038901	3.47078465	0.00051894	0.0212329
ENSG00000169252	154	protein_coding	5	ADRB2	-0.88291048	0.25439898	-3.47057399	0.00051935	0.0212329
ENSG00000181788	6478	protein_coding	3	SIAH2	1.02591491	0.29543519	3.47255487	0.00051553	0.0212329
ENSG00000132825	5509	protein_coding	20	PPP1R3D	0.8774143	0.25321904	3.46504079	0.00053015	0.02149621
ENSG00000183621	220929	protein_coding	10	ZNF438	0.94881156	0.27374935	3.46598659	0.00052829	0.02149621
ENSG00000205056		lncRNA	12	LINC02397	-1.1077489	0.31985761	-3.46325633	0.00053368	0.0215506
ENSG00000086730	7462	protein_coding	7	LAT2	0.71481275	0.20657489	3.46030803	0.00053956	0.02169903

ENSG00000088053	51206	protein_coding	19	GP6	1.29224534	0.37459805	3.44968513	0.00056124	0.02221751
ENSG00000095303	5742	protein_coding	9	PTGS1	0.96061909	0.27843828	3.45002517	0.00056053	0.02221751
ENSG00000177324	139105	protein_coding	X	BEND2	1.10868242	0.32139681	3.44957508	0.00056147	0.02221751
ENSG00000182173	283989	protein_coding	17	TSEN54	-0.76346006	0.22123142	-3.45095679	0.0005586	0.02221751
ENSG00000290038					1.44263678	0.41835812	3.44832979	0.00056406	0.02223092
ENSG00000036448	9172	protein_coding	8	MYOM2	-1.20666853	0.35027262	-3.44494105	0.00057118	0.02242181
ENSG00000035403	7414	protein_coding	10	VCL	0.87861117	0.25529826	3.44150864	0.00057848	0.02261811
ENSG00000151726	2180	protein_coding	4	ACSL1	1.03560053	0.30126325	3.43752693	0.00058705	0.02286256
ENSG00000140022	85439	protein_coding	14	STON2	1.11642253	0.32500191	3.43512609	0.00059228	0.02297526
ENSG00000084070	64744	protein_coding	1	SMAP2	0.87854045	0.25587706	3.43344752	0.00059596	0.02302733
ENSG00000133318	10313	protein_coding	11	RTN3	0.79833328	0.23283509	3.4287498	0.00060637	0.02315364
ENSG00000168461	11031	protein_coding	18	RAB31	0.77804782	0.2269856	3.4277409	0.00060863	0.02315364
ENSG00000182511	2242	protein_coding	15	FES	0.96609175	0.28175771	3.42880321	0.00060625	0.02315364
ENSG00000198502	3127	protein_coding	6	HLA-DRB5	-1.39620478	0.40731244	-3.42784714	0.00060839	0.02315364
ENSG00000101096	4773	protein_coding	20	NFATC2	-0.72219915	0.2109178	-3.42407866	0.00061689	0.02320761
ENSG00000180354	222166	protein_coding	7	MTURN	0.85964287	0.25097248	3.42524749	0.00061424	0.02320761
ENSG00000238005	101927851	lncRNA	1	LNCATV	0.89006573	0.2599506	3.42398029	0.00061711	0.02320761
ENSG00000101265	9770	protein_coding	20	RASSF2	0.71931625	0.21016496	3.42262693	0.00062019	0.02323477
ENSG00000289351		lncRNA	3		1.04322033	0.30494046	3.42106233	0.00062377	0.02328034
ENSG00000101307	10326	protein_coding	20	SIRPB1	0.81717354	0.23908335	3.41794414	0.00063096	0.02345983
ENSG00000132694	9826	protein_coding	1	ARHGEF11	0.90533367	0.26544693	3.4106014	0.0006482	0.02383094
ENSG00000154917	51560	protein_coding	3	RAB6B	1.21171273	0.35511153	3.41220331	0.0006444	0.02383094
ENSG00000198363	444	protein_coding	8	ASPH	1.02133253	0.29945553	3.41063176	0.00064813	0.02383094
ENSG00000161040	222235	protein_coding	7	FBXL13	1.04373816	0.30631252	3.40742901	0.00065578	0.02402006
ENSG00000091106	58484	protein_coding	2	NLRC4	0.87292031	0.2562909	3.40597469	0.00065928	0.02405894
ENSG00000113719	57222	protein_coding	5	ERGIC1	0.86802691	0.2549354	3.40488971	0.00066191	0.0240656
ENSG00000141526	9123	protein_coding	17	SLC16A3	0.79825962	0.23458005	3.40293053	0.00066667	0.02414971
ENSG00000087074	23645	protein_coding	19	PPP1R15A	0.72461344	0.21328229	3.39743842	0.0006802	0.02447255
ENSG00000099308	23031	protein_coding	19	MAST3	0.82578013	0.24306979	3.39729644	0.00068055	0.02447255
ENSG00000166333	3611	protein_coding	11	ILK	0.86047626	0.2535798	3.39331552	0.00069052	0.02465108
ENSG00000179820	91663	protein_coding	19	MYADM	0.81297613	0.23956007	3.39362124	0.00068975	0.02465108

ENSG00000085733	2017	protein_coding	11	CTTN	1.02332269	0.30182705	3.39042731	0.00069784	0.02472868
ENSG00000157551	3772	protein_coding	21	KCNJ15	0.91650542	0.27045419	3.38876406	0.00070208	0.02472868
ENSG00000186517	257106	protein_coding	1	ARHGAP30	0.63741709	0.18803525	3.38988078	0.00069923	0.02472868
ENSG00000210176		mitochondrial	MT	MT-TH	1.11918934	0.33028946	3.38851055	0.00070273	0.02472868
ENSG00000001561	22875	protein_coding	6	ENPP4	-0.9088727	0.26872774	-3.38213201	0.00071926	0.0247343
ENSG00000008130	65220	protein_coding	1	NADK	0.84024364	0.24829952	3.38399218	0.0007144	0.0247343
ENSG00000110931	10645	protein_coding	12	CAMKK2	0.83755158	0.24750888	3.38392532	0.00071457	0.0247343
ENSG00000131626	8500	protein_coding	11	PPFIA1	0.75974334	0.22440456	3.38559674	0.00071024	0.0247343
ENSG00000138735	8654	protein_coding	4	PDE5A	1.07739215	0.31806959	3.38728438	0.00070588	0.0247343
ENSG00000162444	116362	protein_coding	1	RBP7	0.84345823	0.24942059	3.38167044	0.00072047	0.0247343
ENSG00000225889		lncRNA	2		0.84148183	0.2487542	3.38278444	0.00071755	0.0247343
ENSG00000143376	81609	protein_coding	1	SNX27	0.76712509	0.22700284	3.37936345	0.00072654	0.02485621
ENSG00000163464	3577	protein_coding	2	CXCR1	0.83954031	0.24859039	3.37720347	0.00073227	0.02491329
ENSG00000198948					1.03609874	0.30682588	3.37682965	0.00073326	0.02491329
ENSG00000132334	5791	protein_coding	10	PTPRE	0.73542378	0.21798567	3.37372543	0.00074158	0.02510934
ENSG00000120694	10808	protein_coding	13	HSPH1	-0.80034573	0.23743506	-3.37079842	0.00074951	0.0252292
ENSG00000168016	9881	protein_coding	3	TRANK1	0.74559999	0.22121166	3.37052754	0.00075024	0.0252292
ENSG00000172270	682	protein_coding	19	BSG	0.94080019	0.27926985	3.36878543	0.000755	0.02530283
ENSG00000143797	129642	protein_coding	2	MBOAT2	0.89074452	0.26472263	3.36482195	0.00076593	0.02554449
ENSG00000196547	4122	protein_coding	15	MAN2A2	0.83398196	0.24789212	3.36429399	0.0007674	0.02554449
ENSG00000070718	10947	protein_coding	8	AP3M2	-0.77239421	0.22990465	-3.35962852	0.00078047	0.02556075
ENSG00000122862	5552	protein_coding	10	SRGN	0.74114068	0.2203887	3.36287968	0.00077134	0.02556075
ENSG00000143815	3930	protein_coding	1	LBR	0.70956516	0.21126964	3.3585761	0.00078345	0.02556075
ENSG00000145819	23092	protein_coding	5	ARHGAP26	0.85244966	0.25379448	3.35881869	0.00078276	0.02556075
ENSG00000196782	55534	protein_coding	4	MAML3	0.88515815	0.26340768	3.36041138	0.00077826	0.02556075
ENSG00000213085	25790	protein_coding	1	CFAP45	1.02978409	0.30659962	3.35872596	0.00078303	0.02556075
ENSG00000159593	8883	protein_coding	16	NAE1	-0.79943506	0.23845454	-3.35256794	0.00080066	0.02603585
ENSG00000185339	6948	protein_coding	22	TCN2	1.10188572	0.32897265	3.34947515	0.00080965	0.02624166
ENSG00000105339	22898	protein_coding	8	DENND3	0.8073496	0.24195513	3.33677409	0.00084757	0.0266813
ENSG00000138095	10128	protein_coding	2	LRPPRC	-0.71894886	0.21523813	-3.34024864	0.00083703	0.0266813
ENSG00000138449	30061	protein_coding	2	SLC40A1	0.85528085	0.25609211	3.3397391	0.00083857	0.0266813

ENSG00000164330	1879	protein_coding	5	EBF1	-1.02849157	0.30798776	-3.33939106	0.00083962	0.0266813
ENSG00000178726	7056	protein_coding	20	THBD	0.93863978	0.2813043	3.33674169	0.00084767	0.0266813
ENSG00000179583	4261	protein_coding	16	CIITA	-0.78166829	0.23423938	-3.33704892	0.00084673	0.0266813
ENSG00000180509	3753	protein_coding	21	KCNE1	1.05871359	0.31726518	3.33699897	0.00084688	0.0266813
ENSG00000188305	374872	protein_coding	19	PEAK3	1.01498054	0.30434217	3.33499807	0.000853	0.0266813
ENSG00000197208	6583	protein_coding	5	SLC22A4	0.99854032	0.29919609	3.33741105	0.00084563	0.0266813
ENSG00000253729	5591	protein_coding	8	PRKDC	0.81401443	0.24380108	3.33884675	0.00084127	0.0266813
ENSG00000280832		lncRNA	11	GSEC	1.07740423	0.32305458	3.33505326	0.00085283	0.0266813
ENSG00000138411	57520	protein_coding	2	HECW2	0.97226129	0.29227662	3.3265106	0.00087941	0.02724778
ENSG00000143226	2212	protein_coding	1	FCGR2A	0.82743757	0.24862745	3.32802175	0.00087465	0.02724778
ENSG00000180573	8334	protein_coding	6	H2AC6	0.83649055	0.25141618	3.32711499	0.0008775	0.02724778
ENSG00000073849	6480	protein_coding	3	ST6GAL1	-0.71985693	0.21691528	-3.31860864	0.00090467	0.02794271
ENSG00000020633	864	protein_coding	1	RUNX3	-0.70301846	0.21206236	-3.31514967	0.00091594	0.02807234
ENSG00000170525	5209	protein_coding	10	PFKFB3	1.02205072	0.30833883	3.31469998	0.00091742	0.02807234
ENSG00000171236	116844	protein_coding	19	LRG1	0.99722438	0.30071619	3.31616458	0.00091262	0.02807234
ENSG00000155926	6503	protein_coding	8	SLA	0.8073249	0.24370084	3.31277024	0.00092377	0.02817922
ENSG00000178927	79415	protein_coding	17	CYBC1	0.81848785	0.24719495	3.31110266	0.00092929	0.0282602
ENSG00000112335	8724	protein_coding	6	SNX3	0.84726683	0.25615654	3.30761352	0.00094095	0.02826566
ENSG00000130775	9473	protein_coding	1	THEMIS2	0.70307298	0.21255474	3.30772658	0.00094057	0.02826566
ENSG00000166091	116173	protein_coding	14	CMTM5	1.07846899	0.32602237	3.3079601	0.00093978	0.02826566
ENSG00000238113					0.89692914	0.27112839	3.30813434	0.0009392	0.02826566
ENSG00000179818		lncRNA	2	PCBP1-AS1	0.76989842	0.2333515	3.29930776	0.00096924	0.02902699
ENSG00000051523	1535	protein_coding	16	CYBA	0.83373228	0.2528145	3.29780244	0.00097445	0.02909462
ENSG00000160211	2539	protein_coding	X	G6PD	0.92629774	0.2809965	3.29647436	0.00097907	0.02914421
ENSG00000151693	8853	protein_coding	2	ASAP2	1.10866588	0.33656798	3.29403249	0.00098761	0.02922201
ENSG00000284526	122455342	protein_coding	17		1.00952731	0.30643403	3.29443598	0.00098619	0.02922201
ENSG00000099785	51257	protein_coding	19	MARCHF2	0.98398838	0.29945896	3.28588719	0.00101662	0.02994505
ENSG00000134815	9704	protein_coding	19	DHX34	0.84112898	0.2560803	3.28462973	0.00102116	0.02994505
ENSG00000187554	7100	protein_coding	1	TLR5	1.05606005	0.32148703	3.28492274	0.0010201	0.02994505
ENSG00000176928	51301	protein_coding	5	GCNT4	-1.00717791	0.3067534	-3.28334712	0.00102582	0.02999238
ENSG00000096006	10321	protein_coding	6	CRISP3	1.2395388	0.37814755	3.27792362	0.00104574	0.03044264

ENSG00000161921	58191	protein_coding	17	CXCL16	0.78668293	0.24002718	3.27747438	0.0010474	0.03044264
ENSG00000198695	4541	protein_coding	MT	MT-ND6	0.98906248	0.301939	3.27570299	0.00105399	0.03054411
ENSG00000168792	116236	protein_coding	17	ABHD15	-0.82211847	0.25113475	-3.27361494	0.00106181	0.03068045
ENSG00000101493	9658	protein_coding	18	ZNF516	0.80319802	0.24560874	3.27023386	0.00107459	0.0308421
ENSG00000115271	25801	protein_coding	2	GCA	0.86579559	0.26477408	3.26994083	0.0010757	0.0308421
ENSG00000171777	115727	protein_coding	19	RASGRP4	0.84990352	0.25993697	3.26965237	0.0010768	0.0308421
ENSG00000136040	10154	protein_coding	12	PLXNC1	0.8213842	0.25161541	3.26444306	0.0010968	0.0309728
ENSG00000148484	6251	protein_coding	10	RSU1	0.81677585	0.25023315	3.26405935	0.00109828	0.0309728
ENSG00000180739	53637	protein_coding	19	S1PR5	-0.89274544	0.27336149	-3.26580545	0.00109153	0.0309728
ENSG00000183696	7378	protein_coding	7	UPP1	0.9198057	0.28184126	3.26355939	0.00110022	0.0309728
ENSG00000213625	54741	protein_coding	1	LEPROT	0.70031094	0.21446519	3.26538277	0.00109316	0.0309728
ENSG00000214456	440503	protein_coding	19	PLIN5	1.04051734	0.31878977	3.26396092	0.00109866	0.0309728
ENSG00000099331	4650	protein_coding	19	MYO9B	0.71280052	0.21858692	3.26094769	0.00111041	0.03109301
ENSG00000150681	64407	protein_coding	1	RGS18	0.81286128	0.24929686	3.26061578	0.00111171	0.03109301
ENSG00000160410	92799	protein_coding	19	SHKBP1	0.74152062	0.22747555	3.25978159	0.00111498	0.03109301
ENSG00000175003	6580	protein_coding	6	SLC22A1	0.94774038	0.29078576	3.25923929	0.00111711	0.03109301
ENSG00000142669	83442	protein_coding	1	SH3BGRL3	0.80257779	0.24667448	3.25359064	0.00113956	0.03111394
ENSG00000171051	2357	protein_coding	19	FPR1	0.78258317	0.24053659	3.2534891	0.00113997	0.03111394
ENSG00000177663	23765	protein_coding	22	IL17RA	0.79318119	0.24377668	3.25372054	0.00113904	0.03111394
ENSG00000181847	201633	protein_coding	3	TIGIT	-0.89558733	0.2751749	-3.25461128	0.00113548	0.03111394
ENSG00000256235	85027	protein_coding	5	SMIM3	0.92103113	0.28300611	3.2544567	0.00113609	0.03111394
ENSG00000257878		lncRNA	12		0.96677632	0.29688417	3.25640914	0.00112831	0.03111394
ENSG00000260859		lncRNA	16		0.98141125	0.3015192	3.25488809	0.00113437	0.03111394
ENSG00000133943	80017	protein_coding	14	DGLUCY	0.67504477	0.20772577	3.24969193	0.0011553	0.03144525
ENSG00000187037	353345	protein_coding	7	GPR141	1.00887577	0.31054624	3.24871356	0.00115928	0.03146667
ENSG00000110079	51338	protein_coding	11	MS4A4A	0.97635634	0.30068039	3.24715664	0.00116564	0.0315524
ENSG00000185219	353274	protein_coding	3	ZNF445	0.88376	0.2728022	3.23956335	0.00119713	0.03231592
ENSG00000126821	81537	protein_coding	14	SGPP1	-0.76281329	0.23559792	-3.23777597	0.00120465	0.03234183
ENSG00000274173		lncRNA	20	LINC02967	1.34679719	0.41586964	3.23850811	0.00120157	0.03234183
ENSG00000169180	23214	protein_coding	16	XPO6	0.82520421	0.25519358	3.23364013	0.00122223	0.03252802
ENSG00000169891	9185	protein_coding	X	REPS2	0.8863714	0.27416016	3.23304233	0.00122479	0.03252802

ENSG00000213347	83463	protein_coding	5	MXD3	1.00848516	0.31189587	3.23340341	0.00122325	0.03252802
ENSG00000230492		lncRNA	20		1.06176668	0.32823793	3.23474707	0.00121751	0.03252802
ENSG00000163565	3428	protein_coding	1	IFI16	0.84081879	0.26016515	3.23186554	0.00122985	0.03257447
ENSG00000168404	197259	protein_coding	16	MLKL	0.80765589	0.25022691	3.22769399	0.00124792	0.03287646
ENSG00000225195		lncRNA	12		0.86703174	0.26861918	3.22773573	0.00124774	0.03287646
ENSG00000100284	10043	protein_coding	22	TOM1	0.78761619	0.24459827	3.22003994	0.00128173	0.03361634
ENSG00000225936	105378500	lncRNA	10	SLC18A2-AS1	1.04416479	0.32429563	3.21979299	0.00128283	0.03361634
ENSG00000176463	28232	protein_coding	15	SLCO3A1	0.70081356	0.21786156	3.21678394	0.00129636	0.03388078
ENSG00000171476	84525	protein_coding	4	HOPX	-0.93310743	0.29032924	-3.21396301	0.00130917	0.03412488
ENSG00000076662	3385	protein_coding	19	ICAM3	0.66309634	0.20647926	3.21144283	0.0013207	0.03433076
ENSG00000079385	634	protein_coding	19	CEACAM1	0.88656284	0.27612593	3.21071928	0.00132403	0.03433076
ENSG00000073792	10644	protein_coding	3	IGF2BP2	0.98166541	0.30597931	3.20827384	0.00133534	0.03444276
ENSG00000165030	4783	protein_coding	9	NFIL3	0.84820922	0.26435541	3.20859417	0.00133386	0.03444276
ENSG00000078618	4898	protein_coding	1	NRDC	0.72380691	0.22601266	3.20250608	0.00136237	0.03504823
ENSG00000167996	2495	protein_coding	11	FTH1	0.7976522	0.24915698	3.20140413	0.0013676	0.03509094
ENSG00000073150	56666	protein_coding	22	PANX2	0.85401905	0.26707531	3.19767129	0.00138542	0.03545598
ENSG00000082781	3693	protein_coding	3	ITGB5	1.00513944	0.31443179	3.19668518	0.00139017	0.03548524
ENSG00000123689	50486	protein_coding	1	G0S2	1.08006571	0.33803485	3.19513128	0.00139767	0.03558467
ENSG00000075151	8672	protein_coding	1	EIF4G3	0.73916187	0.23141033	3.19416107	0.00140238	0.03561246
ENSG00000014216	823	protein_coding	11	CAPN1	0.7825669	0.24509868	3.19286465	0.00140869	0.03568077
ENSG00000022567	57210	protein_coding	8	SLC45A4	0.69899785	0.21911752	3.19005922	0.00142244	0.03593658
ENSG00000153071	1601	protein_coding	5	DAB2	0.83098921	0.26061326	3.18859145	0.00142968	0.03602715
ENSG00000127947	5782	protein_coding	7	PTPN12	0.67455722	0.21164442	3.1872194	0.00143648	0.03610616
ENSG00000160712	3570	protein_coding	1	IL6R	0.72102648	0.22662164	3.18163127	0.00146448	0.03671637
ENSG00000054523	23095	protein_coding	1	KIF1B	0.8430897	0.26540377	3.17663047	0.00148997	0.0371662
ENSG00000092929	201294	protein_coding	17	UNC13D	0.75069496	0.23626978	3.17727878	0.00148664	0.0371662
ENSG00000138678	84803	protein_coding	4	GPAT3	0.72363456	0.22796075	3.17438231	0.00150156	0.03736073
ENSG00000009694	10178	protein_coding	X	TENM1	0.99740858	0.31457579	3.1706463	0.001521	0.03768769
ENSG00000026025	7431	protein_coding	10	VIM	0.65777794	0.20767662	3.16731822	0.00153852	0.03768769
ENSG00000035862	7077	protein_coding	17	TIMP2	0.78029482	0.2464328	3.16635946	0.0015436	0.03768769
ENSG00000100485	6655	protein_coding	14	SOS2	0.73478782	0.23197894	3.16747633	0.00153768	0.03768769

ENSG00000112195	79865	protein_coding	6	TREML2	0.88057536	0.2782578	3.16460266	0.00155295	0.03768769
ENSG00000154134	64221	protein_coding	11	ROBO3	-1.02030398	0.32240255	-3.16468951	0.00155249	0.03768769
ENSG00000173597	27284	protein_coding	4	SULT1B1	0.89295131	0.28195414	3.16700906	0.00154016	0.03768769
ENSG00000175489	126364	protein_coding	19	LRRC25	0.73108713	0.23084227	3.16704189	0.00153998	0.03768769
ENSG00000185697	4603	protein_coding	8	MYBL1	-0.87585795	0.27630691	-3.16987352	0.00152505	0.03768769
ENSG00000248188		pseudogene	4	PFDN1P2	0.97819704	0.30909275	3.16473629	0.00155224	0.03768769
ENSG00000163545	81788	protein_coding	1	NUAK2	0.74467935	0.23540791	3.16335732	0.00155961	0.0377563
ENSG00000173706	57493	protein_coding	3	HEG1	-0.86155215	0.27245994	-3.16212411	0.00156623	0.03782364
ENSG00000267174		lncRNA	19	TSPAN16-AS1	0.81586934	0.25808375	3.16125804	0.00157089	0.03784353
ENSG00000104918	56729	protein_coding	19	RETN	1.01511316	0.32120951	3.16028367	0.00157616	0.03787772
ENSG00000113368	4001	protein_coding	5	LMNB1	0.85972041	0.27224467	3.15789619	0.00158912	0.03809638
ENSG00000148926	133	protein_coding	11	ADM	0.90874369	0.28800502	3.15530508	0.0016033	0.03825324
ENSG00000237484	105372751	lncRNA	21	LINC01684	-0.88606279	0.28085754	-3.15484779	0.00160582	0.03825324
ENSG00000259379		pseudogene	15	MTND5P32	0.91810876	0.29104024	3.15457666	0.00160731	0.03825324
ENSG00000145332	57563	protein_coding	4	KLHL8	0.78451404	0.24875834	3.15371953	0.00161204	0.03827331
ENSG00000289039		lncRNA	10		1.03539208	0.32882511	3.14876221	0.00163964	0.03883493
ENSG00000154146	4900	protein_coding	11	NRGN	0.93786044	0.29803653	3.14679692	0.0016507	0.03900313
ENSG00000115604	8809	protein_coding	2	IL18R1	1.20094581	0.38198566	3.1439552	0.00166681	0.03910256
ENSG00000140443	3480	protein_coding	15	IGF1R	0.73515587	0.23374158	3.14516518	0.00165993	0.03910256
ENSG00000141480	409	protein_coding	17	ARRB2	0.73989118	0.23531887	3.14420682	0.00166538	0.03910256
ENSG00000196247	51427	protein_coding	7	ZNF107	0.86416535	0.27494622	3.1430341	0.00167206	0.03913264
ENSG00000121316	79887	protein_coding	12	PLBD1	0.79904163	0.2543197	3.14187868	0.00167868	0.0391429
ENSG00000185245	2811	protein_coding	17	GP1BA	1.07139087	0.34103678	3.14156981	0.00168045	0.0391429
ENSG00000105369	973	protein_coding	19	CD79A	-0.88670233	0.28232414	-3.14072445	0.00168531	0.03916347
ENSG00000006194	10127	protein_coding	16	ZNF263	-0.7784041	0.24814375	-3.13690799	0.0017074	0.03958349
ENSG00000185201	10581	protein_coding	11	IFITM2	0.82424699	0.26292906	3.13486451	0.00171934	0.03976669
ENSG00000274275		lncRNA	18		-0.95079174	0.30354887	-3.1322526	0.00173471	0.04002823
ENSG00000069493	29121	protein_coding	12	CLEC2D	-0.63622515	0.20329484	-3.12956867	0.00175063	0.04030134
ENSG00000104892	147700	protein_coding	19	KLC3	1.05144278	0.33604844	3.12884291	0.00175496	0.04030683
ENSG00000147454	51312	protein_coding	8	SLC25A37	0.8249165	0.26374951	3.12765134	0.00176209	0.04037647
ENSG00000130779	6249	protein_coding	12	CLIP1	0.67025106	0.21447673	3.12505266	0.00177773	0.04056063

ENSG00000248323		lncRNA	5	LUCAT1	0.73744357	0.23598582	3.12494864	0.00177836	0.04056063
ENSG00000159958	115650	protein_coding	22	TNFRSF13C	-0.90998586	0.29141255	-3.12267218	0.00179217	0.04074672
ENSG00000197852	55924	protein_coding	1	INKA2	0.76876649	0.24622255	3.12224236	0.00179479	0.04074672
ENSG00000180667	55432	protein_coding	1	YOD1	0.89215051	0.2861338	3.11794874	0.00182114	0.04115536
ENSG00000255801		lncRNA	12		0.97160034	0.31158847	3.11821666	0.00181949	0.04115536
ENSG00000271605	284021	protein_coding	17	MILR1	1.0207022	0.3274347	3.11726947	0.00182535	0.04115592
ENSG00000130449	57688	protein_coding	5	ZSWIM6	0.69680819	0.22373206	3.11447628	0.00184272	0.04145273
ENSG00000157107	115548	protein_coding	5	FCHO2	0.75624821	0.24295042	3.11276773	0.00185342	0.04150393
ENSG00000167995	7439	protein_coding	11	BEST1	0.85534226	0.27478226	3.11280012	0.00185321	0.04150393
ENSG00000150687	11098	protein_coding	11	PRSS23	-0.99196298	0.31883169	-3.11124338	0.00186301	0.04162419
ENSG00000103507	10295	protein_coding	16	BCKDK	0.84912885	0.27311101	3.10909779	0.0018766	0.0418328
ENSG00000108960	23531	protein_coding	17	MMD	0.88973309	0.28636371	3.10700365	0.00188994	0.04203518
ENSG00000168256	28511	protein_coding	17	NKIRAS2	0.72047519	0.23291689	3.0932716	0.00197963	0.04393081
ENSG00000149564	90952	protein_coding	11	ESAM	0.9907883	0.32090987	3.08743477	0.00201892	0.04470208
ENSG00000103056	55512	protein_coding	16	SMPD3	-1.06425723	0.3447865	-3.08671371	0.00202382	0.04471018
ENSG00000133246	84106	protein_coding	19	PRAM1	0.74227325	0.24054438	3.08580579	0.00203001	0.04474659
ENSG00000162881	165140	protein_coding	2	OXER1	0.84911414	0.27566668	3.08022046	0.00206847	0.04539126
ENSG00000279491		protein_coding	11		0.87149495	0.28293242	3.08022304	0.00206846	0.04539126
ENSG00000135842	116496	protein_coding	1	NIBAN1	0.86472711	0.28107878	3.07645818	0.00209476	0.04586587
ENSG00000166439	254225	protein_coding	11	RNF169	0.64875927	0.21095714	3.07531312	0.00210282	0.04594025
ENSG00000135655	9958	protein_coding	12	USP15	0.64288157	0.20915141	3.07376162	0.00211378	0.04607766
ENSG00000100345	4627	protein_coding	22	MYH9	0.68729677	0.22373727	3.07189214	0.00212707	0.04626485
ENSG00000198585	131870	protein_coding	3	NUDT16	0.83441742	0.27174015	3.07064453	0.00213597	0.04635626
ENSG00000119048	7320	protein_coding	5	UBE2B	0.66989077	0.21841843	3.06700662	0.00216214	0.04671836
ENSG00000170542	5272	protein_coding	6	SERPINB9	-0.7143908	0.23291009	-3.06723848	0.00216046	0.04671836
ENSG00000181163	4869	protein_coding	5	NPM1	-0.6817695	0.22237469	-3.0658593	0.00217045	0.04679537
ENSG00000100453	3002	protein_coding	14	GZMB	-0.88505116	0.28881251	-3.06444886	0.00218071	0.04691393
ENSG00000118564	26234	protein_coding	4	FBXL5	0.66820678	0.21810158	3.06374119	0.00218588	0.04691749
ENSG00000126262	2867	protein_coding	19	FFAR2	0.70686461	0.23076602	3.06312255	0.0021904	0.04691749
ENSG00000118520	383	protein_coding	6	ARG1	1.28265498	0.41919943	3.05977269	0.00221505	0.04724003
ENSG00000198900	7150	protein_coding	20	TOP1	0.69562016	0.22734289	3.05978412	0.00221497	0.04724003

ENSG00000101856	10857	protein_coding	X	PGRMC1	0.8008923	0.26189896	3.05802014	0.00222805	0.04731236
ENSG00000131873	22856	protein_coding	15	CHSY1	0.8655679	0.28300676	3.05847082	0.0022247	0.04731236
ENSG00000104763	427	protein_coding	8	ASAH1	0.6449331	0.21109608	3.05516378	0.00224938	0.0476428
ENSG00000169826	55454	protein_coding	10	CSGALNACT2	0.77076772	0.25232652	3.05464408	0.00225328	0.0476428
ENSG00000198189	51170	protein_coding	4	HSD17B11	0.61407822	0.20108492	3.05382538	0.00225944	0.0476707
ENSG00000117868	57488	protein_coding	7	ESYT2	-0.65389181	0.21418583	-3.05291822	0.00226628	0.04771288
ENSG00000132436	63979	protein_coding	7	FIGNL1	-0.94952816	0.31113021	-3.05186746	0.00227422	0.04777812
ENSG00000049239	9563	protein_coding	1	H6PD	0.76317958	0.25039153	3.04794481	0.00230412	0.04829269
ENSG00000160796	23218	protein_coding	3	NBEAL2	0.75586458	0.24814192	3.04609787	0.00231832	0.04829269
ENSG00000170909	126014	protein_coding	19	OSCAR	0.72418553	0.2377136	3.04646228	0.00231552	0.04829269
ENSG00000171159	79095	protein_coding	9	BBLN	0.85194272	0.27960838	3.04691416	0.00231204	0.04829269
ENSG00000182541	3985	protein_coding	22	LIMK2	0.80145568	0.26319324	3.04512264	0.00232585	0.04834735
ENSG00000107485	2625	protein_coding	10	GATA3	-0.65808325	0.2162497	-3.04316373	0.00234105	0.048428
ENSG00000170871	9778	protein_coding	4	KIAA0232	0.66399234	0.21822306	3.04272314	0.00234448	0.048428
ENSG00000171611	171558	protein_coding	6	PTCRA	0.96367523	0.3166528	3.04331817	0.00233985	0.048428
ENSG00000101425	671	protein_coding	20	BPI	1.06744335	0.35102624	3.04092177	0.00235855	0.04851525
ENSG00000148175	2040	protein_coding	9	STOM	0.80698099	0.26536826	3.04098529	0.00235805	0.04851525
ENSG00000169896	3684	protein_coding	16	ITGAM	0.7243476	0.23842854	3.0380071	0.00238148	0.04888491
ENSG00000019995	54764	protein_coding	10	ZRANB1	0.73727114	0.24287386	3.03561341	0.00240047	0.04905829
ENSG00000121858	8743	protein_coding	3	TNFSF10	0.68406248	0.22547187	3.03391498	0.00241402	0.04905829
ENSG00000128266	2781	protein_coding	22	GNAZ	0.90966312	0.2997458	3.03478184	0.0024071	0.04905829
ENSG00000139187	10219	protein_coding	12	KLRG1	-0.77683246	0.25594404	-3.03516527	0.00240404	0.04905829
ENSG00000160683	643	protein_coding	11	CXCR5	-0.85380241	0.28142863	-3.03381497	0.00241482	0.04905829
ENSG00000132471	23558	protein_coding	17	WBP2	0.77333297	0.25506725	3.03187877	0.00243037	0.04918285
ENSG00000188042	10123	protein_coding	2	ARL4C	-0.66637573	0.21983976	-3.03118847	0.00243593	0.04918285
ENSG00000205133	286144	protein_coding	8	TRIQK	0.87439308	0.28844292	3.03142499	0.00243402	0.04918285
ENSG00000197461	5154	protein_coding	7	PDGFA	0.95032626	0.31362159	3.0301685	0.00244417	0.04924834
ENSG00000139631	51380	protein_coding	12	CSAD	0.66940813	0.22109415	3.02770616	0.00246418	0.04934862
ENSG00000276168	6029	short_ncRNA	14	RN7SL1	0.9373869	0.30960104	3.02772526	0.00246402	0.04934862
ENSG00000291113		lncRNA	5		-0.9423453	0.31113791	-3.02870609	0.00245603	0.04934862
ENSG00000005339	1387	protein_coding	16	CREBBP	0.72816561	0.24080226	3.02391515	0.00249526	0.04942224

ENSG00000134755	1824	protein_coding	18	DSC2	0.8777402	0.29004542	3.02621635	0.00247635	0.04942224
ENSG00000139890	161253	protein_coding	14	REM2	0.92025889	0.30435968	3.02358999	0.00249795	0.04942224
ENSG00000142687	79932	protein_coding	1	KIAA0319L	0.76653942	0.25349541	3.02387886	0.00249556	0.04942224
ENSG00000173200	165631	protein_coding	3	PARP15	-0.84625327	0.27983327	-3.02413383	0.00249346	0.04942224
ENSG00000184678	8349	protein_coding	1	H2BC21	0.8359493	0.27632908	3.02519486	0.00248473	0.04942224
ENSG00000161944	433	protein_coding	17	ASGR2	0.80048032	0.26489086	3.02192499	0.00251173	0.0495953
ENSG00000119535	1441	protein_coding	1	CSF3R	0.7269597	0.24065336	3.02077522	0.00252128	0.04968443
ENSG00000158470	9334	protein_coding	20	B4GALT5	0.80654532	0.26718004	3.01873344	0.00253834	0.0498212
ENSG00000196954	837	protein_coding	11	CASP4	0.76791977	0.25438432	3.01873865	0.00253829	0.0498212
ENSG00000136231	10643	protein_coding	7	IGF2BP3	0.95844021	0.31760171	3.01774257	0.00254665	0.049885
ENSG00000100697	23405	protein_coding	14	DICER1	0.6785395	0.22494148	3.0165157	0.00255698	0.04998794
