# Supplementary material for: Same, Same, but Different—Whole Blood Transcriptomes of ANCA-Associated Vasculitis and Giant Cell Arteritis
Source: Int J Mol Sci. 2026 Sep 24;27(19):8513. doi: 10.3390/ijms27198513 (PMC13664966; doi:10.3390/ijms27198513)
Supplement: Supplementary file 1 [file ijms-27-08513-s001.zip › Supplementary Table S4 AAV vs HC GO pathways.pdf]

Supplementary Table S4: List of significantly up- or downregulated pathways between ANCA-associated vasculitis (AAV) and healthy controls (HC) resulting from overrepresentation analysis (ORA) of differentially expressed genes (DEG)

ID	Description	p.adjust	Count
GO:0006959	humoral immune response	8.1299181742787e-06	11
GO:0009617	response to bacterium	1.34567051526988e-05	18
GO:0002227	innate immune response in mucosa	4.72573430574184e-05	5
GO:0042742	defense response to bacterium	7.44959940252582e-05	11
GO:0019731	antibacterial humoral response	7.44959940252582e-05	6
GO:0032103	positive regulation of response to external stimulus	7.44959940252582e-05	17
GO:0061844	antimicrobial humoral immune response mediated by antimicrobial peptide	8.00360061289915e-05	6
GO:0019730	antimicrobial humoral response	8.00360061289915e-05	7
GO:0002385	mucosal immune response	0.000101103495593303	5
GO:0002251	organ or tissue specific immune response	0.000122591095059249	5
GO:0050829	defense response to Gram-negative bacterium	0.000171624986950416	6
GO:0002237	response to molecule of bacterial origin	0.000209774994488365	12
GO:0031349	positive regulation of defense response	0.000358197408043389	14
GO:0001818	negative regulation of cytokine production	0.000358197408043389	11
GO:0002764	immune response-regulating signaling pathway	0.00040544683536967	15
GO:0002274	myeloid leukocyte activation	0.000522850868754314	10
GO:0032496	response to lipopolysaccharide	0.000564288173169078	11
GO:0002253	activation of immune response	0.000564288173169078	15
GO:0002768	immune response-regulating cell surface receptor signaling pathway	0.000807415547771233	12
GO:0002831	regulation of response to biotic stimulus	0.00107190094948726	14
GO:0051607	defense response to virus	0.0018391602976973	11
GO:0140546	defense response to symbiont	0.0018391602976973	11
GO:0002250	adaptive immune response	0.00195689317396103	13
GO:0050729	positive regulation of inflammatory response	0.00241208003394991	7
GO:0009615	response to virus	0.00305279865155099	12
GO:0045088	regulation of innate immune response	0.00340709293325384	12
GO:0031640	killing of cells of another organism	0.00340709293325384	5
GO:0141061	disruption of cell in another organism	0.00340709293325384	5

GO:0042116	macrophage activation	0.00364926853191734	6
GO:0045071	negative regulation of viral genome replication	0.00395566460117003	5
GO:0141060	disruption of anatomical structure in another organism	0.00425269627445483	5
GO:0050832	defense response to fungus	0.00820000908645137	4
GO:0002757	immune response-activating signaling pathway	0.00987497321004762	12
GO:0002694	regulation of leukocyte activation	0.0103667098481253	13
GO:0002833	positive regulation of response to biotic stimulus	0.0122890181839787	10
GO:0086091	regulation of heart rate by cardiac conduction	0.0138339536279483	3
GO:0001906	cell killing	0.0158564989012688	7
GO:0043030	regulation of macrophage activation	0.0181466479330561	4
GO:0001819	positive regulation of cytokine production	0.018438460500512	11
GO:0050865	regulation of cell activation	0.018438460500512	13
GO:0002438	acute inflammatory response to antigenic stimulus	0.018438460500512	3
GO:0071222	cellular response to lipopolysaccharide	0.0187920444076129	7
GO:0009620	response to fungus	0.0196917536033264	4
GO:0086002	cardiac muscle cell action potential involved in contraction	0.020507480615851	3
GO:0006935	chemotaxis	0.020507480615851	9
GO:0042330	taxis	0.020507480615851	9
GO:0042110	T cell activation	0.020507480615851	12
GO:0019079	viral genome replication	0.020507480615851	6
GO:0045069	regulation of viral genome replication	0.020507480615851	5
GO:0044092	negative regulation of molecular function	0.020507480615851	13
GO:0071219	cellular response to molecule of bacterial origin	0.020507480615851	7
GO:0071624	positive regulation of granulocyte chemotaxis	0.020507480615851	3
GO:0090023	positive regulation of neutrophil chemotaxis	0.020507480615851	3
GO:0048525	negative regulation of viral process	0.0210695025240889	5
GO:0002832	negative regulation of response to biotic stimulus	0.0222602306572814	6
GO:0045089	positive regulation of innate immune response	0.0236057689369578	9
GO:1903556	negative regulation of tumor necrosis factor superfamily cytokine production	0.0257953106529114	4
GO:0002252	immune effector process	0.0314017284068106	12
GO:0050921	positive regulation of chemotaxis	0.0325621850818015	5

GO:0002920	regulation of humoral immune response	0.0332840430899937	3
GO:0050920	regulation of chemotaxis	0.0339258326709642	6
GO:0038093	Fc receptor signaling pathway	0.0381309189429295	4
GO:0071216	cellular response to biotic stimulus	0.0389276675927298	7
GO:0050830	defense response to Gram-positive bacterium	0.0397371604731658	4
GO:0002230	positive regulation of defense response to virus by host	0.0436322875875723	3
GO:0090022	regulation of neutrophil chemotaxis	0.0436322875875723	3
GO:1902624	positive regulation of neutrophil migration	0.0436322875875723	3
GO:0002460	adaptive immune response based on somatic recombination of immune receptors built from immunoglo	0.0454637743195621	8
GO:0002526	acute inflammatory response	0.0454637743195621	4
GO:0050727	regulation of inflammatory response	0.0469703786858855	8
GO:0007159	leukocyte cell-cell adhesion	0.0470489443514574	9
GO:0004888	transmembrane signaling receptor activity	0.00186993381688998	13
GO:0038023	signaling receptor activity	0.00186993381688998	15
GO:0060089	molecular transducer activity	0.00186993381688998	15
GO:0001530	lipopolysaccharide binding	0.00397408844734425	4
GO:0019955	cytokine binding	0.04269688306035	5
GO:0004252	serine-type endopeptidase activity	0.04269688306035	4
GO:0004930	G protein-coupled receptor activity	0.04269688306035	6
GO:0035580	specific granule lumen	3.21962806903138e-10	11
GO:0042581	specific granule	2.81454124651233e-09	14
GO:0034774	secretory granule lumen	3.34383489071049e-09	17
GO:0031983	vesicle lumen	3.34383489071049e-09	17
GO:0060205	cytoplasmic vesicle lumen	3.34383489071049e-09	17
GO:0005775	vacuolar lumen	4.05621833479545e-05	9
GO:0009986	cell surface	4.42109613014083e-05	16
GO:0009897	external side of plasma membrane	0.000447781776612031	10
GO:0005766	primary lysosome	0.000484737216632161	8
GO:0042582	azurophil granule	0.000484737216632161	8
GO:0031012	extracellular matrix	0.000756051962999521	8
GO:0035578	azurophil granule lumen	0.000756051962999521	6

GO:0030312	external encapsulating structure	0.000756051962999521	8
GO:1904724	tertiary granule lumen	0.00102047693423981	5
GO:0062023	collagen-containing extracellular matrix	0.00128669705610393	7
GO:0098552	side of membrane	0.0028191961517353	12
GO:0070820	tertiary granule	0.00317242914698209	7
GO:0045121	membrane raft	0.0103941791629716	7
GO:0098857	membrane microdomain	0.0103941791629716	7
