# Supplementary material for: Same, Same, but Different—Whole Blood Transcriptomes of ANCA-Associated Vasculitis and Giant Cell Arteritis
Source: Int J Mol Sci. 2026 Sep 24;27(19):8513. doi: 10.3390/ijms27198513 (PMC13664966; doi:10.3390/ijms27198513)
Supplement: Supplementary file 1 [file ijms-27-08513-s001.zip › Supplementary Table S5 GCA vs HC GO pathways.pdf]

Supplementary Table S5: List of significantly up- or downregulated pathways between giant cell arteritis (GCA) and healthy controls (HC) resulting from overrepresentation analysis (ORA) of differentially expressed genes (DEG)

ID	Description	p.adjust	Count
GO:0007596	blood coagulation	2.99371930985001e-08	28
GO:0050817	coagulation	2.99371930985001e-08	28
GO:0007599	hemostasis	4.48400920272967e-08	28
GO:0042060	wound healing	1.07627322474909e-06	35
GO:0050878	regulation of body fluid levels	1.74703174572122e-06	31
GO:0050865	regulation of cell activation	1.74703174572122e-06	51
GO:0009611	response to wounding	2.98072572629346e-06	39
GO:0006897	endocytosis	6.1255682713887e-06	51
GO:0042742	defense response to bacterium	7.61147793689831e-06	25
GO:0050900	leukocyte migration	1.73705323392934e-05	32
GO:0002274	myeloid leukocyte activation	1.73705323392934e-05	26
GO:0030168	platelet activation	2.15895608015823e-05	18
GO:0006954	inflammatory response	2.15895608015823e-05	51
GO:0006959	humoral immune response	2.15895608015823e-05	19
GO:0050832	defense response to fungus	2.76431291644662e-05	10
GO:0002252	immune effector process	2.93873473656626e-05	46
GO:0006935	chemotaxis	5.00683568751686e-05	30
GO:0042330	taxis	5.00683568751686e-05	30
GO:0009620	response to fungus	7.40294264741048e-05	11
GO:0002694	regulation of leukocyte activation	8.90267356216006e-05	44
GO:0098609	cell-cell adhesion	9.25990699562372e-05	47
GO:0050778	positive regulation of immune response	0.000127704676235372	51
GO:0002366	leukocyte activation involved in immune response	0.000157202758624242	29
GO:0001817	regulation of cytokine production	0.000159302244117959	51
GO:0030595	leukocyte chemotaxis	0.000176683126267295	21
GO:0001818	negative regulation of cytokine production	0.000176683126267295	26
GO:0002263	cell activation involved in immune response	0.000179922049975564	29
GO:0019730	antimicrobial humoral response	0.000247283734072123	11

GO:0002768	immune response-regulating cell surface receptor signaling pathway	0.000320531661954124	31
GO:0006909	phagocytosis	0.000347402178410749	23
GO:0009617	response to bacterium	0.000354612608847989	39
GO:0061844	antimicrobial humoral immune response mediated by antimicrobial peptide	0.000354612608847989	9
GO:0002444	myeloid leukocyte mediated immunity	0.000354612608847989	15
GO:0030195	negative regulation of blood coagulation	0.000405809850183386	7
GO:1900047	negative regulation of hemostasis	0.000405809850183386	7
GO:0097530	granulocyte migration	0.000437953438361209	16
GO:0030193	regulation of blood coagulation	0.000606950533482762	9
GO:1900046	regulation of hemostasis	0.000606950533482762	9
GO:0050819	negative regulation of coagulation	0.000606950533482762	7
GO:0071621	granulocyte chemotaxis	0.000606950533482762	14
GO:0002443	leukocyte mediated immunity	0.000618627403986706	31
GO:0060326	cell chemotaxis	0.000764808864473819	22
GO:0007229	integrin-mediated signaling pathway	0.000975144433307685	13
GO:0050818	regulation of coagulation	0.000995603087085616	9
GO:0002429	immune response-activating cell surface receptor signaling pathway	0.0010341836949421	28
GO:0002697	regulation of immune effector process	0.00114592600445829	29
GO:0002698	negative regulation of immune effector process	0.00121016046795657	14
GO:0002253	activation of immune response	0.00121016046795657	39
GO:0010543	regulation of platelet activation	0.00162527671973434	8
GO:0071345	cellular response to cytokine stimulus	0.00222351552129232	46
GO:0043299	leukocyte degranulation	0.00246137552614921	12
GO:0002703	regulation of leukocyte mediated immunity	0.00253225669048069	22
GO:0006898	receptor-mediated endocytosis	0.00256740725707203	20
GO:0002275	myeloid cell activation involved in immune response	0.00260059107458159	13
GO:1990266	neutrophil migration	0.00349482386903209	13
GO:0097529	myeloid leukocyte migration	0.00358971361372614	18
GO:1903035	negative regulation of response to wounding	0.00384022407251202	9
GO:0032103	positive regulation of response to external stimulus	0.00411079735404423	37
GO:0007186	G protein-coupled receptor signaling pathway	0.00429991945704337	28

GO:0019221	cytokine-mediated signaling pathway	0.00441248827327911	30
GO:0002764	immune response-regulating signaling pathway	0.00484843013133892	36
GO:1903034	regulation of response to wounding	0.00484843013133892	13
GO:0002521	leukocyte differentiation	0.00484843013133892	40
GO:0032102	negative regulation of response to external stimulus	0.00528992762864013	27
GO:0002886	regulation of myeloid leukocyte mediated immunity	0.00572445670483758	10
GO:0050867	positive regulation of cell activation	0.00572445670483758	29
GO:0043300	regulation of leukocyte degranulation	0.00635617243015272	9
GO:0006936	muscle contraction	0.00635617243015272	17
GO:0030593	neutrophil chemotaxis	0.00635617243015272	11
GO:0002283	neutrophil activation involved in immune response	0.00670280633688435	6
GO:0032695	negative regulation of interleukin-12 production	0.00670280633688435	6
GO:0001906	cell killing	0.00675301483191888	17
GO:0002701	negative regulation of production of molecular mediator of immune response	0.00698858086235977	7
GO:0002757	immune response-activating signaling pathway	0.00757084093991742	34
GO:0001819	positive regulation of cytokine production	0.00834514302539388	32
GO:0061045	negative regulation of wound healing	0.00891570918051	7
GO:0043312	neutrophil degranulation	0.00937116154783859	5
GO:0031589	cell-substrate adhesion	0.0101541788084329	20
GO:0003012	muscle system process	0.010897270198585	20
GO:0061041	regulation of wound healing	0.0114444323709128	10
GO:0050866	negative regulation of cell activation	0.0116535074796488	16
GO:0035743	CD4-positive, alpha-beta T cell cytokine production	0.0136997497851685	5
GO:0032692	negative regulation of interleukin-1 production	0.0157413759636927	6
GO:0002448	mast cell mediated immunity	0.0158785419607423	8
GO:0002707	negative regulation of lymphocyte mediated immunity	0.0158785419607423	8
GO:0002704	negative regulation of leukocyte mediated immunity	0.0158785419607423	9
GO:0033674	positive regulation of kinase activity	0.0163435005385472	21
GO:0034109	homotypic cell-cell adhesion	0.0167570718914198	10
GO:0030155	regulation of cell adhesion	0.0170850045155397	39
GO:0070527	platelet aggregation	0.0180229175465139	9

GO:0034612	response to tumor necrosis factor	0.0180527926212919	18
GO:0042116	macrophage activation	0.0182938033574476	11
GO:0050853	B cell receptor signaling pathway	0.018436516350203	10
GO:0002683	negative regulation of immune system process	0.0195930350861459	29
GO:0007188	adenylate cyclase-modulating G protein-coupled receptor signaling pathway	0.0200280269422065	11
GO:0045824	negative regulation of innate immune response	0.0200280269422065	11
GO:0002285	lymphocyte activation involved in immune response	0.0231376762118217	18
GO:0010951	negative regulation of endopeptidase activity	0.0231376762118217	10
GO:0045860	positive regulation of protein kinase activity	0.0231376762118217	19
GO:0030029	actin filament-based process	0.0231376762118217	36
GO:0031640	killing of cells of another organism	0.0231376762118217	8
GO:0141061	disruption of cell in another organism	0.0231376762118217	8
GO:0002251	organ or tissue specific immune response	0.0231376762118217	5
GO:0045342	MHC class II biosynthetic process	0.0231376762118217	5
GO:0002250	adaptive immune response	0.0253103070985171	30
GO:0042119	neutrophil activation	0.0267629500659064	7
GO:0002696	positive regulation of leukocyte activation	0.0267659984101066	26
GO:0032640	tumor necrosis factor production	0.0267659984101066	15
GO:0032680	regulation of tumor necrosis factor production	0.0267659984101066	15
GO:0002446	neutrophil mediated immunity	0.0271274999440717	6
GO:1903131	mononuclear cell differentiation	0.027319060624574	31
GO:0031349	positive regulation of defense response	0.027872675928568	29
GO:0032612	interleukin-1 production	0.027872675928568	11
GO:0032652	regulation of interleukin-1 production	0.027872675928568	11
GO:0030099	myeloid cell differentiation	0.027872675928568	28
GO:0034755	iron ion transmembrane transport	0.0286828263577183	5
GO:0045859	regulation of protein kinase activity	0.0286828263577183	28
GO:0141060	disruption of anatomical structure in another organism	0.0286828263577183	8
GO:0071356	cellular response to tumor necrosis factor	0.0286828263577183	16
GO:0070098	chemokine-mediated signaling pathway	0.0289445039441283	7
GO:0051346	negative regulation of hydrolase activity	0.031711783754906	14

GO:0010466	negative regulation of peptidase activity	0.032229430621364	10
GO:0071706	tumor necrosis factor superfamily cytokine production	0.0335154600261226	15
GO:1903555	regulation of tumor necrosis factor superfamily cytokine production	0.0335154600261226	15
GO:0051249	regulation of lymphocyte activation	0.034167121900887	31
GO:0050729	positive regulation of inflammatory response	0.0349217207736303	12
GO:0050764	regulation of phagocytosis	0.0349994220356761	10
GO:0002699	positive regulation of immune effector process	0.0349994220356761	19
GO:0042445	hormone metabolic process	0.0349994220356761	11
GO:0042113	B cell activation	0.0349994220356761	21
GO:0002695	negative regulation of leukocyte activation	0.0349994220356761	14
GO:0043549	regulation of kinase activity	0.0349994220356761	30
GO:0045576	mast cell activation	0.0355805467869203	8
GO:0042100	B cell proliferation	0.0373514289520808	10
GO:0072359	circulatory system development	0.0373514289520808	41
GO:0014909	smooth muscle cell migration	0.0373514289520808	7
GO:0030036	actin cytoskeleton organization	0.0382190999026644	33
GO:0106106	cold-induced thermogenesis	0.0391823765450446	12
GO:0007160	cell-matrix adhesion	0.0395841138873013	14
GO:0031638	zymogen activation	0.0425448268559932	6
GO:0071674	mononuclear cell migration	0.0425448268559932	14
GO:0002888	positive regulation of myeloid leukocyte mediated immunity	0.0425448268559932	5
GO:0010810	regulation of cell-substrate adhesion	0.0457193127927854	14
GO:0031347	regulation of defense response	0.0487629159144869	40
GO:0038023	signaling receptor activity	5.81657898855329e-16	65
GO:0060089	molecular transducer activity	5.81657898855329e-16	65
GO:0004888	transmembrane signaling receptor activity	2.31829085463458e-15	52
GO:0140375	immune receptor activity	1.12902390255052e-06	19
GO:0005509	calcium ion binding	2.46826050022738e-06	32
GO:0019838	growth factor binding	6.12173682000188e-05	13
GO:0030246	carbohydrate binding	0.000202020868957471	19
GO:0008307	structural constituent of muscle	0.000841230698394565	6

GO:0005125	cytokine activity	0.00110450332935959	11
GO:0048018	receptor ligand activity	0.00138590453448505	15
GO:0019955	cytokine binding	0.00138590453448505	13
GO:0001530	lipopolysaccharide binding	0.00166638314849028	7
GO:0030546	signaling receptor activator activity	0.00166638314849028	15
GO:0019865	immunoglobulin binding	0.00228170041759474	6
GO:0008009	chemokine activity	0.00249629417727049	5
GO:0038024	cargo receptor activity	0.00249629417727049	8
GO:0030545	signaling receptor regulator activity	0.00314154484554088	15
GO:0004896	cytokine receptor activity	0.00316865116621781	10
GO:0004930	G protein-coupled receptor activity	0.00371593577126219	14
GO:0005539	glycosaminoglycan binding	0.00371593577126219	11
GO:0061135	endopeptidase regulator activity	0.00371593577126219	11
GO:0004866	endopeptidase inhibitor activity	0.00655280023560432	9
GO:0030414	peptidase inhibitor activity	0.00724291508895053	9
GO:0001786	phosphatidylserine binding	0.00724291508895053	7
GO:0072341	modified amino acid binding	0.00821068361233334	8
GO:0061134	peptidase regulator activity	0.00928943267935027	13
GO:0008289	lipid binding	0.00997292964035234	38
GO:0005126	cytokine receptor binding	0.0127583723392667	14
GO:0005201	extracellular matrix structural constituent	0.017419061306318	5
GO:0005178	integrin binding	0.017419061306318	10
GO:0038187	pattern recognition receptor activity	0.0186891161633272	6
GO:0003779	actin binding	0.0211220864519184	23
GO:0005537	mannose binding	0.025638386486797	4
GO:0042379	chemokine receptor binding	0.025638386486797	5
GO:1901618	organic hydroxy compound transmembrane transporter activity	0.025638386486797	5
GO:0008201	heparin binding	0.025638386486797	7
GO:0098632	cell-cell adhesion mediator activity	0.0323605645536643	5
GO:0070851	growth factor receptor binding	0.0362750619111903	9
GO:0046982	protein heterodimerization activity	0.0464371114086669	17

GO:0009986	cell surface	7.71703854310214e-13	59
GO:0070820	tertiary granule	1.76168859478965e-11	32
GO:0034774	secretory granule lumen	8.17071041322972e-10	40
GO:0031983	vesicle lumen	8.17071041322972e-10	40
GO:0060205	cytoplasmic vesicle lumen	8.17071041322972e-10	40
GO:0042581	specific granule	1.2901063809658e-08	28
GO:0030667	secretory granule membrane	1.2901063809658e-08	37
GO:0009897	external side of plasma membrane	1.2901063809658e-08	33
GO:0031091	platelet alpha granule	7.39437813128143e-08	16
GO:0098552	side of membrane	1.8437839292824e-07	46
GO:0031093	platelet alpha granule lumen	2.60920138526537e-07	13
GO:0035580	specific granule lumen	4.86942213408855e-07	15
GO:1904724	tertiary granule lumen	6.79892447437888e-06	13
GO:0030312	external encapsulating structure	7.35544441103943e-06	20
GO:0031012	extracellular matrix	7.35544441103943e-06	20
GO:0101003	ficolin-1-rich granule membrane	1.64448973274735e-05	13
GO:0070821	tertiary granule membrane	3.16322374192117e-05	14
GO:0101002	ficolin-1-rich granule	6.30058960613749e-05	24
GO:0062023	collagen-containing extracellular matrix	0.000346257442774115	15
GO:0098590	plasma membrane region	0.000346327903134496	47
GO:0001726	ruffle	0.00103608981778456	18
GO:0001725	stress fiber	0.00122751606361008	9
GO:0097517	contractile actin filament bundle	0.00122751606361008	9
GO:0043235	receptor complex	0.00122751606361008	20
GO:0030139	endocytic vesicle	0.00159271786857176	26
GO:0045121	membrane raft	0.00179799491745938	20
GO:0098857	membrane microdomain	0.0018934309874782	20
GO:0032432	actin filament bundle	0.00201309332458694	9
GO:0042641	actomyosin	0.00289191158134095	9
GO:0098802	plasma membrane signaling receptor complex	0.00453180459465774	13
GO:0005925	focal adhesion	0.00614637312606596	29

GO:0030055	cell-substrate junction	0.00665497963642	29
GO:0071682	endocytic vesicle lumen	0.0076990774581677	5
GO:0035579	specific granule membrane	0.00798393106145118	12
GO:0030016	myofibril	0.0126868565542229	13
GO:0070161	anchoring junction	0.0129996967732075	40
GO:0043292	contractile fiber	0.0145539630601365	13
GO:0031252	cell leading edge	0.0234750848282476	24
GO:0098797	plasma membrane protein complex	0.0234750848282476	22
GO:0099081	supramolecular polymer	0.0239025382573428	35
GO:0099512	supramolecular fiber	0.0239025382573428	35
GO:0044194	cytolytic granule	0.029941535266053	4
GO:0005775	vacuolar lumen	0.0353413096202181	13
GO:0002102	podosome	0.036885720572051	5
GO:0030666	endocytic vesicle membrane	0.036885720572051	14
GO:0044853	plasma membrane raft	0.0378105523254162	8
GO:0031528	microvillus membrane	0.0470626151379815	4
