# Supplementary material for: Same, Same, but Different—Whole Blood Transcriptomes of ANCA-Associated Vasculitis and Giant Cell Arteritis
Source: Int J Mol Sci. 2026 Sep 24;27(19):8513. doi: 10.3390/ijms27198513 (PMC13664966; doi:10.3390/ijms27198513)
Supplement: Supplementary file 1 [file ijms-27-08513-s001.zip › Supplementary Table S6A AAV vs HC GSEA KEGG pathways.pdf]

Supplementary Table S6A: List of signaling pathways between ANCA-associated vasculitis (AAV) and healthy controls (HC) resulting from gene set enrichment analysis using KEGG

ID	Description	setSize	enrichmentScore	p.adjust
hsa03010	Ribosome	166	-0.754840443118492	1.17333333333333e-08
hsa04613	Neutrophil extracellular trap formation	162	0.609110112656805	1.17333333333333e-08
hsa04142	Lysosome	128	0.606941657848981	1.17333333333333e-08
hsa04610	Complement and coagulation cascades	61	0.712596628099811	2.19635864166741e-08
hsa04144	Endocytosis	240	0.508314549633	2.19635864166741e-08
hsa04621	NOD-like receptor signaling pathway	160	0.563594216745875	8.4170200355096e-08
hsa04810	Regulation of actin cytoskeleton	198	0.512332576914685	2.47999708043231e-07
hsa05164	Influenza A	145	0.537754294682653	4.51485097913936e-06
hsa05171	Coronavirus disease - COVID-19	196	-0.445429358748149	5.61139255838155e-06
hsa04145	Phagosome	135	0.542695721474206	6.66897658511925e-06
hsa05160	Hepatitis C	134	0.538768411361865	1.17112357401702e-05
hsa05165	Human papillomavirus infection	292	0.438743732689677	1.92913564632708e-05
hsa04611	Platelet activation	113	0.552735593670681	2.27448235391958e-05
hsa04820	Cytoskeleton in muscle cells	196	0.481682094048875	2.27448235391958e-05
hsa04022	cGMP-PKG signaling pathway	143	0.507199191569555	4.97498187152244e-05
hsa04140	Autophagy - animal	160	0.503950555204561	5.26144656519619e-05
hsa03008	Ribosome biogenesis in eukaryotes	83	-0.545096931142851	6.3534720348371e-05
hsa04666	Fc gamma R-mediated phagocytosis	90	0.568236364191661	6.52960761518616e-05
hsa04148	Efferocytosis	151	0.502242048054654	9.07999798718141e-05
hsa05152	Tuberculosis	152	0.499265991077083	0.000122473010897342
hsa04380	Osteoclast differentiation	125	0.519708912227433	0.000184570071325348
hsa05150	Staphylococcus aureus infection	57	0.618241255233807	0.000184884051441239
hsa05135	Yersinia infection	134	0.501247462202594	0.000226500706883577
hsa05131	Shigellosis	226	0.443458337726141	0.000226500706883577
hsa05130	Pathogenic Escherichia coli infection	181	0.462815279281163	0.000226671077793413
hsa05417	Lipid and atherosclerosis	184	0.453570891012729	0.000360448125242621
hsa05132	Salmonella infection	235	0.436108435594009	0.000360448125242621
hsa05163	Human cytomegalovirus infection	198	0.443956234283925	0.00037249440822888

hsa05200	Pathways in cancer	464	0.376314178426536	0.000373729449329896
hsa05168	Herpes simplex virus 1 infection	160	0.47326524882974	0.000485112901012628
hsa04015	Rap1 signaling pathway	184	0.44719489853047	0.000535244998188765
hsa04935	Growth hormone synthesis, secretion and action	106	0.511007850169645	0.000654515033029587
hsa04062	Chemokine signaling pathway	174	0.459791223719866	0.000718964931185661
hsa04518	Integrin signaling	133	0.484928444974321	0.000858864847542538
hsa05207	Chemical carcinogenesis - receptor activation	156	0.460241854961405	0.000962017442578464
hsa05202	Transcriptional misregulation in cancer	169	0.448299305814679	0.00101133334601538
hsa04081	Hormone signaling	148	0.46778540814098	0.00107126375959165
hsa04152	AMPK signaling pathway	111	0.491831795540735	0.00119546634897768
hsa05161	Hepatitis B	146	0.468369248857406	0.00119546634897768
hsa04510	Focal adhesion	180	0.442691246142411	0.00119546634897768
hsa04270	Vascular smooth muscle contraction	104	0.499079230269166	0.00151738765694749
hsa05231	Choline metabolism in cancer	90	0.506575445710863	0.00154409004532304
hsa04217	Necroptosis	130	0.462613764473726	0.00175417352930419
hsa03040	Spliceosome	163	-0.386371933079089	0.00175417352930419
hsa05170	Human immunodeficiency virus 1 infection	187	0.430966776696364	0.0019953774985465
hsa05418	Fluid shear stress and atherosclerosis	124	0.473475385999677	0.00201445888356147
hsa05133	Pertussis	69	0.535248954812912	0.00204621301820971
hsa05212	Pancreatic cancer	75	0.528367236836405	0.00205242024093768
hsa05100	Bacterial invasion of epithelial cells	74	0.537003740147164	0.00224416885839272
hsa05167	Kaposi sarcoma-associated herpesvirus infection	171	0.434364083271292	0.00236646366802966
hsa05146	Amoebiasis	90	0.497379029072819	0.00242006511013852
hsa04068	FoxO signaling pathway	121	0.470858311496636	0.00291007198386184
hsa04620	Toll-like receptor signaling pathway	88	0.507403818474369	0.00353956542048651
hsa04668	TNF signaling pathway	112	0.471026193066029	0.00398609237966701
hsa04910	Insulin signaling pathway	124	0.456979773062714	0.00435419203731703
hsa00512	Mucin type O-glycan biosynthesis	34	0.63509304013816	0.00437332287392151
hsa04726	Serotonergic synapse	85	0.497143818249001	0.00451247466581124
hsa04082	Neuroactive ligand signaling	130	0.443138707187391	0.00451247466581124
hsa05203	Viral carcinogenesis	191	0.415351594436995	0.00508899032465941

hsa05169	Epstein-Barr virus infection	182	0.417203374656438	0.0058076792091141
hsa05034	Alcoholism	155	0.430063685434531	0.00696872299799399
hsa03030	DNA replication	36	-0.576506519715307	0.00702490192380875
hsa05211	Renal cell carcinoma	66	0.515285575070543	0.00785515469764991
hsa05340	Primary immunodeficiency	35	-0.584746746477557	0.0078984135465971
hsa05140	Leishmaniasis	70	0.502789934862797	0.0078984135465971
hsa04722	Neurotrophin signaling pathway	110	0.442981663046111	0.0078984135465971
hsa05221	Acute myeloid leukemia	64	0.521358797090489	0.00816169093722156
hsa04721	Synaptic vesicle cycle	64	0.521293062501713	0.00816169093722156
hsa00564	Glycerophospholipid metabolism	89	0.469679133641343	0.009454671107409
hsa04978	Mineral absorption	47	0.556177268845557	0.00956949090375487
hsa04920	Adipocytokine signaling pathway	61	0.517991493240254	0.00956949090375487
hsa04912	GnRH signaling pathway	77	0.493475411463038	0.00956949090375487
hsa04926	Relaxin signaling pathway	117	0.4497543478393	0.00956949090375487
hsa04625	C-type lectin receptor signaling pathway	98	0.456584301962024	0.00956949090375487
hsa05162	Measles	121	0.442733622816065	0.00956949090375487
hsa04936	Alcoholic liver disease	112	0.447424017026237	0.00956949090375487
hsa04921	Oxytocin signaling pathway	127	0.435514533175955	0.00956949090375487
hsa04930	Type II diabetes mellitus	40	0.577383680234182	0.00975609944105128
hsa04137	Mitophagy - animal	100	0.453125724405463	0.0107320611214651
hsa05134	Legionellosis	54	0.528834580729965	0.0114525644204068
hsa05205	Proteoglycans in cancer	182	0.404272823487074	0.0116225327149669
hsa04010	MAPK signaling pathway	260	0.375367227927403	0.0118025384061699
hsa03320	PPAR signaling pathway	59	0.517956255196708	0.011874033152621
hsa00970	Aminoacyl-tRNA biosynthesis	44	-0.514994698223897	0.0121524934253552
hsa04150	mTOR signaling pathway	142	0.420664956472968	0.0128173583471105
hsa04066	HIF-1 signaling pathway	99	0.459223534442659	0.0128481575133924
hsa01521	EGFR tyrosine kinase inhibitor resistance	74	0.4854397350008	0.0130444858657135
hsa04928	Parathyroid hormone synthesis, secretion and action	102	0.449280335979399	0.0137286075739624
hsa05223	Non-small cell lung cancer	67	0.482699747451497	0.0142983962877886
hsa04211	Longevity regulating pathway	82	0.464851726563516	0.0147610076536832

hsa05220	Chronic myeloid leukemia	75	0.479477838188258	0.0148824824616546
hsa04670	Leukocyte transendothelial migration	100	0.446098119458679	0.01496371695407
hsa04814	Motor proteins	169	0.398607588730003	0.01496371695407
hsa05322	Systemic lupus erythematosus	108	0.449914767631123	0.0155525296626919
hsa03430	Mismatch repair	23	-0.635722153327347	0.0156944557378027
hsa04917	Prolactin signaling pathway	63	0.479852107670183	0.0159229453132858
hsa04915	Estrogen signaling pathway	106	0.438815175036652	0.015976430010508
hsa04623	Cytosolic DNA-sensing pathway	65	0.486529560048055	0.0161331556190768
hsa04961	Endocrine and other factor-regulated calcium reabsorption	48	0.516650442874703	0.0163609181490717
hsa00600	Sphingolipid metabolism	51	0.516611801908919	0.0163689855094668
hsa04020	Calcium signaling pathway	192	0.382375627702337	0.0163723314075313
hsa03410	Base excision repair	44	-0.503201472778949	0.0167387964329065
hsa04933	AGE-RAGE signaling pathway in diabetic complications	93	0.439576696846726	0.0168739874638354
hsa04725	Cholinergic synapse	93	0.43889723298597	0.0168739874638354
hsa05332	Graft-versus-host disease	36	-0.533486133973854	0.0171450493588746
hsa04216	Ferroptosis	39	0.564634633858577	0.0171450493588746
hsa04540	Gap junction	73	0.476272111762614	0.0171450493588746
hsa04151	PI3K-Akt signaling pathway	294	0.357238655330216	0.0174811635061808
hsa01522	Endocrine resistance	91	0.441465057521981	0.0175747682840728
hsa04919	Thyroid hormone signaling pathway	114	0.424294883890507	0.0175747682840728
hsa04931	Insulin resistance	99	0.446236394088763	0.0179896898949666
hsa04330	Notch signaling pathway	57	0.494404885198376	0.0197871522384707
hsa04664	Fc epsilon RI signaling pathway	60	0.48256608373508	0.0209690821524871
hsa04927	Cortisol synthesis and secretion	50	0.513072317024967	0.0219140574822521
hsa04630	JAK-STAT signaling pathway	125	0.418443497530301	0.0232389973920463
hsa04950	Maturity onset diabetes of the young	12	0.741924637119967	0.0237439742252033
hsa04971	Gastric acid secretion	63	0.462751232196691	0.0237439742252033
hsa04014	Ras signaling pathway	196	0.371195975454032	0.0237439742252033
hsa04979	Cholesterol metabolism	39	0.550227527944388	0.0270215126470782
hsa00982	Drug metabolism - cytochrome P450	37	0.540974922267738	0.0270215126470782
hsa04730	Long-term depression	51	0.498440051896107	0.0270215126470782

hsa05218	Melanoma	59	0.485154821722677	0.0270215126470782
hsa05214	Glioma	67	0.462271771014848	0.0270215126470782
hsa04724	Glutamatergic synapse	96	0.431768585963684	0.0270215126470782
hsa04218	Cellular senescence	151	0.400233967725753	0.0270215126470782
hsa04261	Adrenergic signaling in cardiomyocytes	134	0.404641535176579	0.0277363163350599
hsa00531	Glycosaminoglycan degradation	18	0.66459415140537	0.0278191774551994
hsa04659	Th17 cell differentiation	101	-0.37214743054057	0.0280666650262878
hsa04071	Sphingolipid signaling pathway	120	0.409282839517166	0.0292446470709246
hsa04024	cAMP signaling pathway	177	0.389510577306732	0.0292940140090548
hsa04918	Thyroid hormone synthesis	62	0.466885548414125	0.0295604682483183
hsa00562	Inositol phosphate metabolism	75	0.452525981191148	0.0300400918079294
hsa04728	Dopaminergic synapse	114	0.407680785900516	0.0300400918079294
hsa05225	Hepatocellular carcinoma	150	0.389334809726809	0.0300400918079294
hsa04072	Phospholipase D signaling pathway	133	0.407660486697141	0.0319973874942079
hsa04973	Carbohydrate digestion and absorption	35	0.542170552354892	0.0326917596692736
hsa00480	Glutathione metabolism	47	0.496262165872284	0.0332872302954265
hsa04371	Apelin signaling pathway	122	0.402214586599112	0.0333632328870187
hsa04622	RIG-I-like receptor signaling pathway	55	0.488210828713161	0.0334954025872322
hsa01524	Platinum drug resistance	67	0.451709235632102	0.0344764335698044
hsa05144	Malaria	43	0.500423397473956	0.0351964747550733
hsa04924	Renin secretion	57	0.470300754700616	0.0359411647530069
hsa05142	Chagas disease	96	0.422430020787274	0.0359411647530069
hsa04530	Tight junction	150	0.382966474154539	0.0359411647530069
hsa04658	Th1 and Th2 cell differentiation	85	-0.383758977657964	0.0363117175967375
hsa04210	Apoptosis	128	0.392655020134189	0.0390712658384493
hsa04115	p53 signaling pathway	73	0.444932484571274	0.0405904875994935
hsa00983	Drug metabolism - other enzymes	58	0.457621430425157	0.0409435333429026
hsa04360	Axon guidance	162	0.378288008515326	0.0409435333429026
