# Supplementary material for: Same, Same, but Different—Whole Blood Transcriptomes of ANCA-Associated Vasculitis and Giant Cell Arteritis
Source: Int J Mol Sci. 2026 Sep 24;27(19):8513. doi: 10.3390/ijms27198513 (PMC13664966; doi:10.3390/ijms27198513)
Supplement: Supplementary file 1 [file ijms-27-08513-s001.zip › Supplementary Table S7A GCA vs HC GSEA Kegg pathways.pdf]

Supplementary Table S7A: List of signaling pathways between giant cell arteritis (GCA) and healthy controls (HC) resulting from gene set enrichment analysis using KEGG

ID	Description	setSize	enrichmentScore	pvalue	p.adjust
hsa03010	Ribosome	166	-0.687447717904526	1e-10	7.02e-09
hsa04613	Neutrophil extracellular trap formation	163	0.652115580597151	1e-10	7.02e-09
hsa04611	Platelet activation	113	0.641965051302859	1e-10	7.02e-09
hsa04810	Regulation of actin cytoskeleton	200	0.587998864302381	1e-10	7.02e-09
hsa04144	Endocytosis	240	0.552995866878453	1e-10	7.02e-09
hsa04610	Complement and coagulation cascades	62	0.726540900978715	1.70735322166333e-10	9.98801634673050e-09
hsa05130	Pathogenic Escherichia coli infection	181	0.555584845171165	3.56409696896809e-10	1.56374754513475e-08
hsa05131	Shigellosis	227	0.525748027602892	3.37691859044853e-10	1.56374754513475e-08
hsa04145	Phagosome	135	0.588175925747046	1.50171764164065e-09	5.85669880239853e-08
hsa03008	Ribosome biogenesis in eukaryotes	83	-0.614488272121521	1.83617540396661e-09	6.44497566792279e-08
hsa04062	Chemokine signaling pathway	174	0.545984665442209	3.10299664344403e-09	9.90138019862595e-08
hsa04820	Cytoskeleton in muscle cells	198	0.527240397373531	3.6430787678318e-09	1.0656005395908e-07
hsa04015	Rap1 signaling pathway	184	0.530706289282718	6.02828390718113e-09	1.62763665493891e-07
hsa05135	Yersinia infection	134	0.573459388143956	1.35189643040011e-08	3.38939747907456e-07
hsa04621	NOD-like receptor signaling pathway	160	0.550677086929061	2.75997947566336e-08	6.45835197305225e-07
hsa04510	Focal adhesion	181	0.526663311317418	3.04323288066292e-08	6.67609213195429e-07
hsa05152	Tuberculosis	152	0.53550638327284	1.01438341655471e-07	2.09440340712178e-06
hsa04148	Efferocytosis	151	0.53427463252098	1.13501168484648e-07	2.21327278545064e-06
hsa04666	Fc gamma R-mediated phagocytosis	90	0.598347403767417	1.91670720396637e-07	3.54086436101157e-06
hsa04140	Autophagy - animal	160	0.526229525973455	3.13427169784258e-07	5.23871126639402e-06
hsa05132	Salmonella infection	236	0.478791989805578	2.98660370120028e-07	5.23871126639402e-06
hsa04022	cGMP-PKG signaling pathway	143	0.532830388124757	5.97246269734266e-07	9.52879275803306e-06
hsa05163	Human cytomegalovirus infection	198	0.489445331085011	6.85686428045147e-07	1.04641711410368e-05
hsa05034	Alcoholism	156	0.510338768487198	9.55877354573571e-07	1.39797063106385e-05
hsa04270	Vascular smooth muscle contraction	104	0.572232295798245	1.04116894931641e-06	1.46180120484024e-05
hsa04380	Osteoclast differentiation	125	0.542692646681236	1.6972438753703e-06	2.23234973520317e-05
hsa05167	Kaposi sarcoma-associated herpesvirus infection	171	0.505632677475151	1.71719210400244e-06	2.23234973520317e-05
hsa04670	Leukocyte transendothelial migration	101	0.563747449641148	2.75338893130042e-06	3.45156969602303e-05

hsa05417	Lipid and atherosclerosis	184	0.478890805176743	3.10150470479349e-06	3.62876050460839e-05
hsa05200	Pathways in cancer	464	0.398003346417688	3.10084140431563e-06	3.62876050460839e-05
hsa05165	Human papillomavirus infection	292	0.43329584159077	4.37912165608589e-06	4.95829581060048e-05
hsa04142	Lysosome	128	0.514413512055687	6.17997630887139e-06	6.7786615137933e-05
hsa05418	Fluid shear stress and atherosclerosis	124	0.528174615161686	7.26711208313328e-06	7.72956467024176e-05
hsa04726	Serotonergic synapse	86	0.560518561856425	1.49352428252575e-05	0.000154184418578394
hsa04081	Hormone signaling	149	0.489714185106183	1.62751115225353e-05	0.000163216118411711
hsa04928	Parathyroid hormone synthesis, secretion and action	102	0.532334180065641	1.72913134591514e-05	0.000168590306226726
hsa04512	ECM-receptor interaction	74	0.571401926609476	1.92134625175864e-05	0.000178130098987936
hsa05231	Choline metabolism in cancer	90	0.54616973742858	1.9284740061372e-05	0.000178130098987936
hsa05205	Proteoglycans in cancer	182	0.467434812909873	2.25592637913497e-05	0.000203033374122147
hsa05133	Pertussis	69	0.594077927950325	2.39881965401503e-05	0.000210496424639819
hsa04540	Gap junction	73	0.567515472200544	2.906013797925e-05	0.000248783132456506
hsa05120	Epithelial cell signaling in Helicobacter pylori infection	63	0.594004186824666	3.1036425867968e-05	0.000259375844753733
hsa04721	Synaptic vesicle cycle	64	0.606063401323606	3.32774293247526e-05	0.000270526512109777
hsa05100	Bacterial invasion of epithelial cells	74	0.564374946210802	3.39121553641886e-05	0.000270526512109777
hsa05134	Legionellosis	54	0.607654197763259	3.65412424257694e-05	0.000285021690921002
hsa04066	HIF-1 signaling pathway	99	0.518158986928976	5.90789747485415e-05	0.000449358893138356
hsa04814	Motor proteins	170	0.46098308690215	6.01705640384694e-05	0.000449358893138356
hsa04910	Insulin signaling pathway	124	0.502016578936325	6.27132180088735e-05	0.000458590406689888
hsa05211	Renal cell carcinoma	66	0.584919105764344	7.30207549769597e-05	0.00050214087255316
hsa04926	Relaxin signaling pathway	117	0.502538417946137	7.4391240378246e-05	0.00050214087255316
hsa04517	IgSF CAM signaling	258	0.420398570737602	7.07403625350142e-05	0.00050214087255316
hsa04151	PI3K-Akt signaling pathway	294	0.410710890752996	7.28307962429599e-05	0.00050214087255316
hsa05146	Amoebiasis	90	0.525839604339554	7.71351795725255e-05	0.000503329655331291
hsa04935	Growth hormone synthesis, secretion and action	106	0.509795792304632	7.74353315894294e-05	0.000503329655331291
hsa04620	Toll-like receptor signaling pathway	88	0.534156078721006	8.85797379162484e-05	0.000554262952925192
hsa04668	TNF signaling pathway	112	0.503733122414543	9.00085137228945e-05	0.000554262952925192
hsa05202	Transcriptional misregulation in cancer	169	0.456925689771788	8.81967770868034e-05	0.000554262952925192
hsa04010	MAPK signaling pathway	260	0.417586635811447	9.50831028790545e-05	0.000575416708802554
hsa05150	Staphylococcus aureus infection	57	0.591774539733341	0.000132507239072173	0.000788305778209031

hsa05161	Hepatitis B	146	0.458424418115592	0.000171151002444473	0.00100123336430017
hsa04730	Long-term depression	51	0.605877635975638	0.00018605637806176	0.00105331917257545
hsa04068	FoxO signaling pathway	121	0.486520256548427	0.000183896090915878	0.00105331917257545
hsa05203	Viral carcinogenesis	192	0.439286864221743	0.00019848207525437	0.00110582870498864
hsa04371	Apelin signaling pathway	123	0.476249989856448	0.000219488348051028	0.00119019849903689
hsa03040	Spliceosome	163	-0.379843744620955	0.000220407129451276	0.00119019849903689
hsa04072	Phospholipase D signaling pathway	133	0.464748982086636	0.000254567496361761	0.00135383623065118
hsa04921	Oxytocin signaling pathway	128	0.471426181554434	0.000300335596071677	0.00157339991374863
hsa05221	Acute myeloid leukemia	64	0.56955764615369	0.00033240606376187	0.0017158018879473
hsa04630	JAK-STAT signaling pathway	126	0.465819692108155	0.000337337371398417	0.00171602054146151
hsa01521	EGFR tyrosine kinase inhibitor resistance	74	0.524407743060888	0.000393454094348174	0.00195200983156648
hsa04024	cAMP signaling pathway	177	0.433929397033259	0.000394850991570428	0.00195200983156648
hsa05414	Dilated cardiomyopathy	91	0.493520829182357	0.000456149337552763	0.00222372802056972
hsa04915	Estrogen signaling pathway	107	0.479497961442036	0.000463418374883913	0.00222821711759251
hsa05160	Hepatitis C	134	0.464855662862414	0.000505867888618735	0.00239945444466454
hsa05142	Chagas disease	96	0.497906616056824	0.000522797451122556	0.00244669207125356
hsa04530	Tight junction	152	0.440883895822507	0.000611587724548616	0.00282456962258637
hsa01522	Endocrine resistance	91	0.487088549035797	0.000623712162851327	0.00284315544364696
hsa03320	PPAR signaling pathway	59	0.550622097462681	0.000681324241048434	0.00306595908471795
hsa04722	Neurotrophin signaling pathway	110	0.476568986758903	0.000707603657746681	0.00314390992239348
hsa00970	Aminoacyl-tRNA biosynthesis	44	-0.537914420621462	0.000828762145419329	0.0036361939130273
hsa05144	Malaria	43	0.583257449072886	0.000853335839325598	0.0036526936536986
hsa04217	Necroptosis	130	0.46085170831883	0.000851066389614343	0.0036526936536986
hsa04664	Fc epsilon RI signaling pathway	60	0.540077010723258	0.00087976266347579	0.00372044210698798
hsa04360	Axon guidance	162	0.43398649875563	0.000906539991148147	0.00378804210586904
hsa04012	ErbB signaling pathway	75	0.516667403558415	0.00099234964188755	0.00409782028591212
hsa05207	Chemical carcinogenesis - receptor activation	156	0.431912944726821	0.00113163983993561	0.00461866957927208
hsa04020	Calcium signaling pathway	193	0.413142496891694	0.0011669538672238	0.00470805525742015
hsa05340	Primary immunodeficiency	35	-0.587357657552792	0.00123775636832905	0.00493696006003974
hsa05170	Human immunodeficiency virus 1 infection	187	0.413093582074202	0.00132254328914261	0.00521587297178716
hsa05140	Leishmaniasis	70	0.514539800197477	0.00144651117638398	0.00564139358789752

hsa04520	Adherens junction	89	0.481544382126124	0.00169224311193495	0.00652722343174908
hsa04625	C-type lectin receptor signaling pathway	98	0.468064121503917	0.00173529988809649	0.00662054631219422
hsa05410	Hypertrophic cardiomyopathy	86	0.48280575185922	0.00182753802528591	0.00682410475399313
hsa05164	Influenza A	145	0.433497252698955	0.00182177375241901	0.00682410475399313
hsa04919	Thyroid hormone signaling pathway	114	0.449391558648233	0.00187902895644191	0.00694251751274852
hsa04912	GnRH signaling pathway	77	0.511171885827145	0.00206306129885287	0.0075430678739308
hsa05215	Prostate cancer	98	0.464335685722904	0.00220969111315184	0.0079958925847041
hsa03030	DNA replication	36	-0.538301510928665	0.00228575782684938	0.00818674486963399
hsa04713	Circadian entrainment	83	0.498978648811475	0.00236659886151856	0.00839066869083854
hsa04060	Cytokine-cytokine receptor interaction	229	0.388583038365926	0.00286710929396235	0.0100635536218078
hsa04936	Alcoholic liver disease	112	0.449108088434278	0.0029033004154634	0.010089687582452
hsa05332	Graft-versus-host disease	36	-0.528114061702655	0.00298743429618318	0.0102802886074539
hsa00515	Mannose type O-glycan biosynthesis	23	-0.615738964344223	0.00356720336377883	0.0121561978707415
hsa05032	Morphine addiction	69	0.503657319801268	0.00380858811147158	0.0128539848762166
hsa04740	Olfactory transduction	111	0.440242019605452	0.00410682096002183	0.0137285157806444
hsa05213	Endometrial cancer	57	0.528053039441771	0.00434596507859822	0.0142572674383887
hsa05223	Non-small cell lung cancer	67	0.50252413050889	0.00434623252395326	0.0142572674383887
hsa00512	Mucin type O-glycan biosynthesis	34	0.590067225577239	0.00442430158317176	0.0143789801453082
hsa05220	Chronic myeloid leukemia	75	0.487395111496185	0.00447421839335232	0.0144078041840978
hsa04150	mTOR signaling pathway	142	0.426115879090925	0.00507954768667232	0.0162083748911089
hsa04971	Gastric acid secretion	63	0.496653573774034	0.00556385534392362	0.017593812844299
hsa05322	Systemic lupus erythematosus	110	0.438090538346373	0.0056451936465582	0.0176916336601958
hsa05212	Pancreatic cancer	75	0.484711402942922	0.00576988073036894	0.0179223728881371
hsa05214	Glioma	67	0.489681180548381	0.00693539487770877	0.0213537158076823
hsa04924	Renin secretion	58	0.495162279357776	0.00716997441033716	0.0218840088524204
hsa03020	RNA polymerase	34	-0.523071234880797	0.00725690437456072	0.0219583916850932
hsa04961	Endocrine and other factor-regulated calcium reabsorption	48	0.526157863511518	0.00766749997516757	0.0228279404907355
hsa04137	Mitophagy - animal	100	0.437913797975443	0.00767435036440681	0.0228279404907355
hsa04728	Dopaminergic synapse	115	0.420534139905752	0.00785335329841745	0.0231640925020548
hsa05218	Melanoma	59	0.500586868359657	0.00800216126024195	0.0234063216862077
hsa05206	MicroRNAs in cancer	167	0.398402697845085	0.0082044649835719	0.0237997290019317

hsa04082	Neuroactive ligand signaling	130	0.421709801272319	0.00881604532757142	0.0253641959834227
hsa04966	Collecting duct acid secretion	22	0.633975792728032	0.00901706951763849	0.0257316374039928
hsa05224	Breast cancer	125	0.422217105564627	0.0101113561134087	0.0286216612565037
hsa04978	Mineral absorption	48	0.520052305154136	0.0102419490444931	0.0287593929169365
hsa04330	Notch signaling pathway	57	0.506415659428386	0.0104709513436642	0.0291690787430646
hsa05145	Toxoplasmosis	106	0.41872964603348	0.0106578718243643	0.0294560079555267
hsa05219	Bladder cancer	40	0.556952304916007	0.0111021215173936	0.0304440988484778
hsa04933	AGE-RAGE signaling pathway in diabetic complications	93	0.430178588764691	0.0112816174562371	0.0304603671318401
hsa03013	Nucleocytoplasmic transport	104	-0.361776530353224	0.0111983923951055	0.0304603671318401
hsa05210	Colorectal cancer	85	0.44143573639298	0.0118654888930813	0.0315514136475118
hsa04261	Adrenergic signaling in cardiomyocytes	135	0.40832739573223	0.011836079797838	0.0315514136475118
hsa04972	Pancreatic secretion	72	0.457541730962715	0.011955975831789	0.0315529888493078
hsa04930	Type II diabetes mellitus	40	0.555410922469103	0.0121398106741543	0.0317990563181205
hsa00601	Glycosphingolipid biosynthesis - lacto and neolacto series	26	0.590449143372848	0.0124674002563389	0.0324152406664812
hsa04350	TGF-beta signaling pathway	102	0.417600346772944	0.0131711261174581	0.0339931269649102
hsa04672	Intestinal immune network for IgA production	44	-0.462053490951287	0.0135240637023556	0.0345165781650651
hsa04658	Th1 and Th2 cell differentiation	85	-0.374769614958358	0.0135706204751538	0.0345165781650651
hsa04917	Prolactin signaling pathway	63	0.474933384046131	0.0142225222516528	0.0359144266930227
hsa04725	Cholinergic synapse	93	0.423497943395359	0.0147371655079426	0.0369481792377704
hsa04920	Adipocytokine signaling pathway	61	0.485577419854027	0.0151760335927819	0.0377786368160741
hsa03430	Mismatch repair	23	-0.557213003974775	0.0159369593347767	0.0391179910944519
hsa05225	Hepatocellular carcinoma	150	0.387990496087233	0.0159097321262552	0.0391179910944519
hsa04979	Cholesterol metabolism	40	0.544919613432572	0.0167643799286642	0.0405813610686976
hsa04014	Ras signaling pathway	196	0.372841587622076	0.0166725506734553	0.0405813610686976
hsa04640	Hematopoietic cell lineage	88	0.425055502147019	0.0178589848144108	0.0429349566428642
hsa05022	Pathways of neurodegeneration - multiple diseases	428	0.332511315115639	0.0185471973914791	0.0442861652000623
hsa05330	Allograft rejection	33	-0.506915047665466	0.0190729401685132	0.0452337972915416
hsa04622	RIG-I-like receptor signaling pathway	55	0.481654082634782	0.0194423517135561	0.0458004392715314
hsa04657	IL-17 signaling pathway	77	0.463617849553343	0.0198713234001273	0.0464988967562978
hsa05320	Autoimmune thyroid disease	34	-0.474978805973479	0.0212282635302103	0.0490205295993672
hsa05412	Arrhythmogenic right ventricular cardiomyopathy	76	0.44866035105412	0.021149187164049	0.0490205295993672

hsa04071	Sphingolipid signaling pathway	120	0.406858280088986	0.0217866140261035	0.0497506389929421
hsa04931	Insulin resistance	99	0.407559191319156	0.021827915683513	0.0497506389929421
hsa05030	Cocaine addiction	43	0.49918331439205	0.0224410662948139	0.0498532548701246
hsa04720	Long-term potentiation	55	0.477468588109347	0.0222982619438873	0.0498532548701246
hsa04724	Glutamatergic synapse	96	0.415694092145592	0.0223707055549535	0.0498532548701246
hsa05168	Herpes simplex virus 1 infection	160	0.387405655426702	0.0222252087154937	0.0498532548701246
