# Supplementary material for: Same, Same, but Different—Whole Blood Transcriptomes of ANCA-Associated Vasculitis and Giant Cell Arteritis
Source: Int J Mol Sci. 2026 Sep 24;27(19):8513. doi: 10.3390/ijms27198513 (PMC13664966; doi:10.3390/ijms27198513)
Supplement: Supplementary file 1 [file ijms-27-08513-s001.zip › Supplementary Table S8A AAV vs GCA GSEA KEGG pathways.pdf]

Supplementary Table S8A: List of signaling pathways between ANCA-associated vasculitis (AAV) and giant cell arteritis (GCA) resulting from gene set enrichment analysis using KEGG

ID	Description	setSize	enrichmentScore	pvalue	p.adjust
hsa03010	Ribosome	166	-0.523688955270711	3.06497736064672e-09	1.07887203094765e-06
hsa05171	Coronavirus disease - COVID-19	196	-0.468429738864285	2.08037014789741e-07	3.66145146029944e-05
hsa05164	Influenza A	145	0.476710427100141	1.08527199207793e-06	0.000127338580403081
hsa05160	Hepatitis C	134	0.453224597017676	1.95681124050377e-05	0.00172199389164332
hsa05168	Herpes simplex virus 1 infection	160	0.431243060107518	3.46056585156897e-05	0.00203019863292046
hsa05169	Epstein-Barr virus infection	182	0.421227153738641	3.20820481507036e-05	0.00203019863292046
hsa04512	ECM-receptor interaction	73	-0.542865249397433	5.13061072407965e-05	0.00233420758429233
hsa04611	Platelet activation	113	-0.488176160004547	5.30501723702803e-05	0.00233420758429233
hsa05130	Pathogenic Escherichia coli infection	181	-0.430431561389663	7.86931690299239e-05	0.00285617799872041
hsa04510	Focal adhesion	180	-0.424418359949576	8.11414204181935e-05	0.00285617799872041
hsa04517	IgSF CAM signaling	257	-0.376924416465705	0.000174958305785668	0.00559866578514138
hsa04820	Cytoskeleton in muscle cells	196	-0.400828192421984	0.000275275970419961	0.00807476179898553
hsa04672	Intestinal immune network for IgA production	44	0.574224599723127	0.000367704193550702	0.00922838357839054
hsa04612	Antigen processing and presentation	70	0.508560784640616	0.00036997953857332	0.00922838357839054
hsa05131	Shigellosis	226	-0.38448299811482	0.000393254982033688	0.00922838357839054
hsa04520	Adherens junction	88	-0.454361150081149	0.000856910524585417	0.0188520315408792
hsa05310	Asthma	23	0.659776938961643	0.000957525317524565	0.0196413847204289
hsa04610	Complement and coagulation cascades	61	-0.515263313241424	0.00100665752370533	0.0196413847204289
hsa05162	Measles	121	0.41020284431118	0.00106018837979588	0.0196413847204289
hsa04613	Neutrophil extracellular trap formation	162	-0.400706465276285	0.00112849380354635	0.0198614909424158
hsa04814	Motor proteins	169	-0.39426077645689	0.00123955468177 4	0.0207772975230689
hsa04670	Leukocyte transendothelial migration	100	-0.437887774774038	0.00167524445159566	0.0268039112255306
hsa00650	Butanoate metabolism	21	0.668093279076299	0.0017608096190096	0.0268617978016624
hsa04142	Lysosome	128	0.397116100907191	0.00183148621374971	0.0268617978016624
hsa05330	Allograft rejection	33	0.574337493356289	0.00245558039135389	0.0327350904520602
hsa04623	Cytosolic DNA-sensing pathway	65	0.4768539761338	0.00251093023353871	0.0327350904520602
hsa04810	Regulation of actin cytoskeleton	198	-0.377415827797752	0.00234785640858348	0.0327350904520602
hsa04518	Integrin signaling	133	-0.409370632997831	0.00294760698117163	0.036898274188278

hsa04060	Cytokine-cytokine receptor interaction	229	-0.359532294066249	0.00303991463482972	0.036898274188278
hsa04015	Rap1 signaling pathway	184	-0.372906073493248	0.00316558760731619	0.03714289459251
hsa04144	Endocytosis	240	-0.358483058225955	0.00351963546927744	0.0399648930705052
hsa05332	Graft-versus-host disease	36	0.544636419169344	0.00380382229946051	0.0418420452940656
