# Supplementary material for: Same, Same, but Different—Whole Blood Transcriptomes of ANCA-Associated Vasculitis and Giant Cell Arteritis
Source: Int J Mol Sci. 2026 Sep 24;27(19):8513. doi: 10.3390/ijms27198513 (PMC13664966; doi:10.3390/ijms27198513)
Supplement: Supplementary file 1 [file ijms-27-08513-s001.zip › Supplementary Table S8B AAV vs GCA GSEA MSigDB pathways.pdf]

Supplementary Table S8B: List of signaling pathways between ANCA-associated vasculitis (AAV) and giant cell arteritis (GCA) resulting from gene set enrichment analysis using MSigDB

ID	setSize	enrichmentScore	pvalue	p.adjust
HALLMARK_INTERFERON_ALPHA_RESPONSE	97	0.708633687163736	1e-10	2.5e-09
HALLMARK_INTERFERON_GAMMA_RESPONSE	199	0.623120106008295	1e-10	2.5e-09
HALLMARK_EPITHELIAL_MESENCHYMAL_TRANSITION	182	-0.458939649382325	2.1522063973	3.5870106622161e-05
HALLMARK_COAGULATION	112	-0.490747322421092	2.1349742068	0.000266871775851346
HALLMARK_E2F_TARGETS	200	0.398572813595468	5.1280809167	0.000512808091679376
HALLMARK_G2M_CHECKPOINT	198	0.386671549183573	0.0001680352	0.00140029378494314
HALLMARK_MYOGENESIS	172	-0.413114829968668	0.0002664410	0.00190315071248357
HALLMARK_APICAL_JUNCTION	184	-0.402546752730488	0.0003408108	0.00213006779119852
HALLMARK_ANGIOGENESIS	33	-0.649800065462103	0.0005109886	0.00283882583758599
HALLMARK_INFLAMMATORY_RESPONSE	180	-0.376880183770411	0.0015384132	0.00769206637366794
HALLMARK_KRAS_SIGNALING_UP	180	-0.367435323873015	0.0032492836	0.0147694711402285
HALLMARK_HEME_METABOLISM	193	0.344617489150345	0.0045629892	0.0190124551739345
HALLMARK_MTORC1_SIGNALING	198	0.331763681649144	0.0085092570	0.0327279115986046
HALLMARK_P53_PATHWAY	193	-0.347459207717294	0.0098780874	0.0352788839284068
HALLMARK_UNFOLDED_PROTEIN_RESPONSE	111	0.369632960669713	0.0120590267	0.0401967559032523
HALLMARK_OXIDATIVE_PHOSPHORYLATION	200	0.324523197309174	0.0147265681	0.0460205256151724
